# hnRNPK S379 phosphorylation participates in migration regulation of triple negative MDA-MB-231 cells

**DOI:** 10.1038/s41598-019-44063-z

**Published:** 2019-05-20

**Authors:** Hsin-Yu Tsai, Shu-Ling Fu, Ling-Ming Tseng, Jen-Hwey Chiu, Chao-Hsiung Lin

**Affiliations:** 1 Department of Life Sciences and Institute of Genome Sciences, National Yang-Ming University, Taipei, Taiwan; 2 Institute of Traditional Medicine, National Yang-Ming University, Taipei, Taiwan; 3 Institute of Biopharmaceutical Sciences, National Yang-Ming University, Taipei, Taiwan; 4 Proteomics Research Center, National Yang-Ming University, Taipei, Taiwan; 5 Aging and Health Research Center, National Yang-Ming University, Taipei, Taiwan; 6 Comprehensive Breast Health Center, Taipei Veterans General Hospital, Taipei, Taiwan

**Keywords:** Breast cancer, Epithelial-mesenchymal transition, Phosphoproteins

## Abstract

We have previously identified a novel Aurora-A-mediated Serine 379 (S379) phosphorylation of a poly(C)-binding protein, hnRNPK, the overexpression of which is frequently observed in various cancers. It is known that the oncogenic Aurora-A kinase promotes the malignancy of cancer cells. This study aims to investigate the unexplored functions of hnRNPK S379 phosphorylation using MDA-MB-231 cells, a triple negative breast cancer cell that has amplification of the Aurora-A kinase gene. Accordingly, we established two cell lines in which the endogenous hnRNPK was replaced with either S379D or S379A hnRNPK respectively. Notably, we found that a phosphorylation-mimic S379D mutant of hnRNPK suppressed cell migration and, conversely, a phosphorylation-defective S379A mutant promoted migration. Moreover, Twist was downregulated upon hnRNPK S379 phosphorylation, whereas β-catenin and MMP12 were increased when there was loss of hnRNPK S379 phosphorylation in MDA-MB-231 cells. Furthermore, S379A hnRNPK increases stability of β-catenin in MDA-MB-231 cells. In conclusion, our results suggest that hnRNPK S379 phosphorylation regulates migration via the EMT signaling pathway.

## Introduction

Triple negative breast cancer (TNBC) cells are a subtype of breast cancer and these cells lack both progesterone receptors (PRs) and estrogen receptors (Ers), as well as exhibiting a low level of Her2 expression. In addition, TNBC patients typically have a poorer outcome than other breast cancer patients. TNBC accounts for about 40% of African American breast cancer patients and between 15% and 30% of Asian breast cancer patients^1^. It has been shown that TNBC cells are highly malignant and often metastasize into the lymph nodes, the lungs, the brain and bone; for example, up to 40% of TNBC patients are diagnosed with positive lymph metastasis^2–4^. Further extensive studies of the mechanisms behind TNBC and the treatment of TNBC metastasis are needed.

Aurora-A, an oncogenic kinase, is amplified in several cancer types^5–9^ and has been shown to promote cancer cell proliferation and metastasis^10–15^. In addition, an increased copy number and a higher mRNA level of Aurora-A have been observed in various breast cancers, which implies that Aurora-A is a putative promoter of breast cancer progression^16,17^. For example, Aurora-A-mediated phosphorylation of YAP, a transcription factor, has been shown to enhance the proliferation of TNBC cells^11^. On the other hand, inhibition of Aurora-A is known to suppress TNBC cell proliferation and prevents TNBC metastasis^13^.

Human heterogeneous nuclear ribonucleoprotein K (hnRNPK) is a member of the hnRNP family and has been frequently been observed to show abnormal expression in many human cancers. In addition, hnRNPK is known to be involved in anti-apoptosis^18,19^, angiogenesis, proliferation, and the metastasis of cancer cells^20–22^. It has been suggested that hnRNPK is likely to play an important role in cancer progression^23^; nevertheless, the regulatory roles of hnRNPK in cancer cells has remained ambiguous. Notably, diverse post-transcriptional modifications (PTMs) of hnRNPK, including ubiquitination^24^, SUMOylation^25^, methylation^26–28^ and phosphorylation^29–34^, have been identified and these changes have been shown to affect the functioning of the protein. Our laboratory has previously reported that hnRNPK S379 is phosphorylated by Aurora-A, both *in vitro* and *in vivo*^35^, but the functional role of hnRNPK S379 phosphorylation in cancer cells has remained unexplored. Both hnRNPK and Aurora-A have been shown to promote carcinogenesis; furthermore Aurora-A activity is known to play an important role in TNBC progression. Therefore, the aim of the present study was to investigate whether hnRNPK S379 phosphorylation is involved in Aurora-A-mediated cell proliferation/metastasis using MDA-MB-231 cells, a typical TBNC cell line.

The present study shows that migration ability was higher in MDA-MB-231 cells carrying a S379 phosphorylation-defective hnRNPK gene (MDA-MB-S379A cells) than in cells carrying either the wild-type hnRNPK (MDA-MB-WT cells) or cells carrying a S379 phosphorylation-mimic hnRNPK gene (MDA-MB-S379D cells), which implies that hnRNPK S379 phosphorylation negatively regulates cancer cell migration. In addition, we demonstrated that hnRNPK S379 phosphorylation undergoes crosstalk with the asymmetric dimethylation of arginine 296 and 299 within the protein-interactive region of hnRNPK. Furthermore, the migration ability of MDA-MB-231 cells seems to be independently influenced by both S379 phosphorylation and R296/R299 arginine methylation. In summary, our findings suggest a novel correlation and a relationship between hnRNPK S379 phosphorylation and TNBC metastasis.

## Materials and Methods

### Antibodies

Antibodies against Flag-M2, hnRNPK/J and β-actin were purchased from Sigma-Aldrich. Antibody against GAPDH was obtained from ENOGENE. Antibody against β-catenin was acquired from BD Biosciences. Antibody against Twist was purchased from GeneTex. Antibody against Aurora-A was obtained from Abcam as was anti-mono/dimethyl arginine antibody. Finally, a specific antibody was custom ordered from GeneTex that allowed detection of hnRNPK S379 phosphorylation.

### Chemicals and reagent kits

Protease Inhibitor Cocktail, Aurora-A Inhibitor-I (AAI), nocodazole, Phosphatase Inhibitor Cocktail and Flag-tagged beads were purchased from Sigma-Aldrich. 1,4-dithiothreitol (DTT) and isopropyl β-D-1-thiogalactopyranoside (IPTG) were obtained from Acros Organics. Insect cell-expressed Aurora-A was purchased from Invitrogen. The cDNA reverse transcription kits and TurboFect reagent were purchased from Thermo Fisher Scientific. Hygromycin B and the Midi plasmid extraction kits were obtained from Roche. PVDF membrane, iCRT3 and ECL were purchased from Merck Millipore. The NTA-beads were purchased from QUIAGEN. The protein G beads for immunoprecipitation were obtained from GE. RQ1 (RNase-free DNase) was purchased from Promega. The SYBR Green reagents (the KAPA SYBR FAST qPCR kit Master Mix (2X) ABI Prism) were obtained from KAPA.

### Plasmids

Transfection plasmid containing the Flag-tagged wild-type (WT) hnRNPK with shRNA resistance from a previous study was modified by site-directed mutation kit to create the hnRNPK mutants S379A and S379D^26^. These plasmids, namely WT hnRNPK, S379A hnRNPK and S379D hnRNPK, were then individually introduced into the pLKO AS3W.hyg lentivirus plasmid using Nhe1/Asc1 digestion. The new plasmids were transformed into *E. coli* DH5α and each plasmid was then purified from *E. coli* using a Plasmid Midi Kit (Roche); the purified plasmid was then packaged into virus. The lentivirus for hnRNPK knockdown was obtained from National RNAi Core Facility (Taipei, Taiwan). The resulting virus particles carrying the WT hnRNPK, S379A hnRNPK and S379D hnRNPK genes were used to individually infect the mammalian cell lines, MDA-MB-231 and U2OS. Alternatively, the WT, S379A or S379D hnRNPK segments of the plasmids were individually cloned into the EcoRI/XhoI sites of the pGEX-4t-1 and pET23a-Trx vectors to construct plasmids for *E. coli* BL21(DE3) transformation. The transformed *E. coli* strains were used to produce GST-tagged or His_6_/Trx-tagged recombinant proteins, respectively. PRMT1-pGEX-4t-1 plasmid was prepared as described in previous reports^26,35^ and then used to produce recombinant GST-PRMT1 for the *in vitro* methylation assay.

### Generation of pre-methylated hnRNPK

Recombinant GST-PRMT1 and *in vitro* methylation were performed as described in previous reports^11,12^. Briefly, GST-PRMT1 protein was incubated with the His_6_/Trx-tagged hnRNPK in the presence of [methyl-^3^H]-S-adenosyl methionine (SAM) in Tris-HCl (25 mM, pH8.0) at 30 °C for 16 h. This pre-methylated hnRNPK protein was then re-purified using NTA-beads by following manufacturer’s instructions. The protein was then stored at −80 °C for subsequent use in the *in vitro* kinase assay.

### *In vitro* kinase assay

The *in vitro* Aurora-A-mediated kinase assay was performed as described in a previous report^35^. Briefly, recombinant hnRNPK was incubated with commercial Aurora-A kinase (Invitrogen) and reaction buffer (50 mM Tris-HCl at pH 7.4, 50 mM NaCl and 10 mM MgCl_2_) on ice for 10 min. Subsequently, the reactions were placed at 37 °C for 16 h in the presence or absence (control) of 0.1 mM ATP. After stopping the kinase reaction using SDS sample buffer, the levels of *in vitro* phosphorylation mediated by Aurora-A were determined by Western blot analysis using a hnRNPK S379 phosphorylation-specific antibody.

### Generation of various stable cell lines

We established stable clones of, MDA-MB-231 and U2OS cells that steadily expressed Flag-S379A hnRNPK, Flag-S379D hnRNPK or Flag-WT hnRNPK by replacing the endogenous hnRNPK. To establish the cells carring the Flag-WT/shRNA resistant hnRNPK, the Flag-mutant S379A/shRNA resistance hnRNPK or the Flag-mutant S379D/shRNA resistance hnRNPK, 2 × 10^5^ MDA-MB-231 cells or 6 × 10^5^ U2OS cells were individually seeded into 6-well plates for 24 h of incubation before lentivirus infection. The lentivirus that harbored each of the mutant/shRNA-resistant hnRNPKs was added into the culture medium. After 24 h of infection, the infected-cells were selected using 100 μg/mL hygromycin B. After stable expression of each mutant/shRNA-resistant hnRNPK, the endogenous hnRNPK in each of the stable cell clones was knockdown by lentivirus that expressed sh56 shRNA against endogenous hnRNPK. These infected-cells were then selected by 3 ng/mL puromycin. After antibiotic selection, all the stable clone cells were cultured in DMEM medium supplemented with 10% FBS, 1%PSG, 50 μg/mL hygromycin B and 3 ng/mL puromycin and then used for the various experimental analyses.

### Transfection

Transient transfection was performed using TurboFect (Thermo), which was diluted in opti-MEM medium. The process is according to the manufacturer’s instruction.

### Immunoprecipitation

Cell lysates were lysed by three freeze-thaw cycles with lysis buffer (0.5% Triton-X-100/PBS containing protease inhibitor cocktail and phosphatase inhibitor). Protein G beads were first washed with cold PBS to remove the non-specific binding. To immunoprecipitate Flag-hnRNPKs from the various cell lysates, 1 μg Flag-M2 antibody were incubated with 2 mg of total cell lysates, 20 μl protein G beads and lysis buffer at 4 °C for 16 h. Subsequently, the beads were washed using cold PBS to remove the unbound proteins. The bound proteins were eluted from the beads using SDS-sample buffer and the resulting samples analyzed by SDS-PAGE and immunoblotting.

### Reverse-transcription

Total RNA was extracted from the various cell lysates using the TRIzol/Chloroform method in order to remove DNA. Next, 2 μg of total RNA from each lysate was incubated with RQ1 at 37 °C for 20 min and the RQ1 was then inactivated by heating at 70 °C for 10 min. Random primers were used for the RT-PCR and the RT-PCR procedure was the same as that described by the manufacturer (Reverse transcription kit (Thermo)). Finally, the cDNAs products were stored at −20 °C.

### Quantitative PCR (qPCR)

A total of 4 µl of 0.05X diluted cDNAs were mixed with SYBR Green reagent, forward primers and reverse primers using a 96-well PCR plate and the procedure was as described by the manufacturer of the SYBR Green reagent. The real-time PCR signals were measured using a StepOnePlus™ Real-Time PCR System (Applied Biosystems). GAPDH and β-actin were as the internal controls.

### Western blot analysis

The ratio of acrylamide and bis-acrylamide for the SDS-PAGE gel was 37:1, and the total acrylamide concentration was about 10% (w/v). Proteins were first separated by SDS-PAGE and then transferred to PVDF membranes. Next the membranes were incubated in blocking buffer (5% non-fat milk in TBS-T) for 1 h at room temperature before being incubated with the diluted primary antibody at 4 °C for 16 h. After incubation with the primary antibody, the membranes were washed three times with TBS-T buffer. Each membrane was subsequently incubated with HRP-conjugated secondary antibody at room temperature for 1 h. This was followed by treatment with the ECL Western blotting detecting reagents. Finally, the protein signals were detected using a LAS-3000 image system.

### Transwell migration assay

The 24-Transwells were purchased from Greiner bio-one. Initially, MDA-MB-231 or U2OS cells (3 × 10^4^/well) were suspended in serum-free DMEM medium and the cells were then individually dispensed into the upper chambers of the 24-Transwells. Each well in lower 24-well plate had 600 µl of 10%FBS DMEM medium added. The plates were then incubated for 20 h. At the end of the incubation, the migrated cells were fixed using 10% formaldehyde and then stained using crystal violate. The number of migrated cells in each Transwell cell was then analyzed by microscopy and ImageJ software.

### Wound healing assay

Various MDA-MB-231 and U2OS cell lines (5 × 10^5^/well) were seeded into a 6-well plates in the presence of 10% FBS DMEM medium for 24 h. The medium in each 6 well plate was then changed into 1% FBS DMEM medium before making a wound with a 10 µl plastic pipette tip. The cells that had migrated into the wound were then calculated by being photographed using a microscope. These measurements look place 24 h or 48 h after the scratch was created.

### MTT assay

A total of 5000 cells in DMEM medium were seeded into a 96-well plate and incubated with or without drug treatment for 24 h. MTT (3-(4,5-Dimethylthiazol-2-yl)-2,5-diphenyltetrazolium bromide) in DMEM medium was then added into each well to a final concentration of 0.5 mg/mL and further incubated at 37 °C for 4 h. Next, a solubilizing solution (12% SDS and 45% DMF in ddH_2_O at pH 4.7) was added into each well and then incubated at 37 °C for another 16 h to lyse cells. Finally, the absorbance at 550 nm for each well was measured to indicate cell viability.

### Statistical analysis

Data from three or more than three independent experiments are expressed as means ± standard deviation (s.d.). Statistical analyses were performed by GraphPad Prism using Student *t*-test. Statistical comparisons were considered significantly different if *p* values < 0.05.

## Results

### Aurora-A kinase is positively correlated with hnRNPK S379 phosphorylation and cell migration of MDA-MB-231 cells

**Figure 1:**
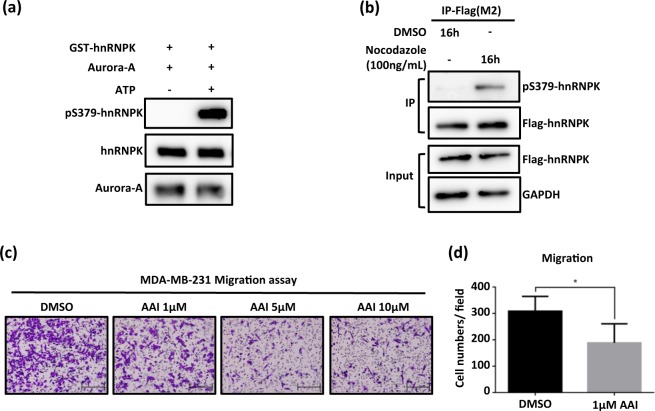
Aurora-A phosphorylates hnRNPK at Serine 379 and its kinase activity is correlated with breast cancer cell migration. (**a**) Detection of *in vitro* hnRNPK S379 phosphorylation mediated by Aurora-A kinase. Recombinant GST-hnRNPK proteins were incubated with a commercial Aurora-A kinase in the presence or absence of ATP at 37 °C for 16 h. Levels of hnRNPK S379 phosphorylation were determined by Western blot analysis using S379 phosphorylation-specific polyclonal antibodies. (**b**) Detection of *in vivo* hnRNPK S379 phosphorylation in HEK293 cells upon amplification of Aurora-A. HEK293 cells were transfected with Flag-hnRNPK and synchronized at G2/M phase using nocodazole to amplify Aurora-A. Subsequently, hnRNPK was immunoprecipitated using Flag antibody and the protein analyzed to measure its S379 phosphorylation level. (**c**) Aurora-A kinase activity is correlated with the migration of MDA-MB-231 cells. A total of 3 × 10^4^ MDA-MB-231 cells were seeded into Transwells for the migration assay in the presence of DMSO only, 1 μM, 5 μM and 10 μM of Aurora-A inhibitor (AAI). After 20 h, the migrated cells were fixed using 10% formaldehyde and stained with crystal violet for imaging analysis. (**d**) Quantification of suppression of migration of MDA-MB-231 cells upon inhibition of Aurora-A kinase. Counts of migrated cells per field in triplicate experiments were quantified in bar graphs with standard deviation. Data is shown as mean ± SD, and * indicates significant difference compared to wild type (*p < 0.05, n = 4). The full blot images are shown in Supplementary Fig. S5.

Aurora-A, a mitosis-regulating kinase with oncogenic activity, promotes proliferation and metastasis in several types of cancer cells^12,14,15^. Using the polyclonal antibody against the S379 phosphorylation of hnRNPK developed by us, we first demonstrated that such phosphorylation was obviously increased *in vitro* by Aurora-A kinase activity (Fig. 1a). In addition, we further showed *in vivo* that the S379 phosphorylation level of exogenous hnRNPK in HEK293 cells is strongly induced under G2/M phase arrest, which is when Aurora-A is activated^36^. Our findings indicated that the S379 phosphorylation level of hnRNPK is increased upon treatment with nocodazole, a cell-cycle inhibitor (Fig. 1b). Amplification of Aurora-A has been reported in breast cancer cells^37,38^, and therefore we next investigated whether Aurora-A activity is correlated with migration ability of TNBC cells, namely MDA-MB-231 cells. As shown in Fig. 1c,d and Supplementary Fig. S1, a commercial Aurora-A inhibitor, AAI^39^, when included in the migration assay, clearly suppressed migration of MDA-MB-231 cells when compared to the vehicle control.

### hnRNPK S379 phosphorylation regulates the migration of MDA-MB-231 cells in an Aurora-A-independent manner

**Figure 2:**
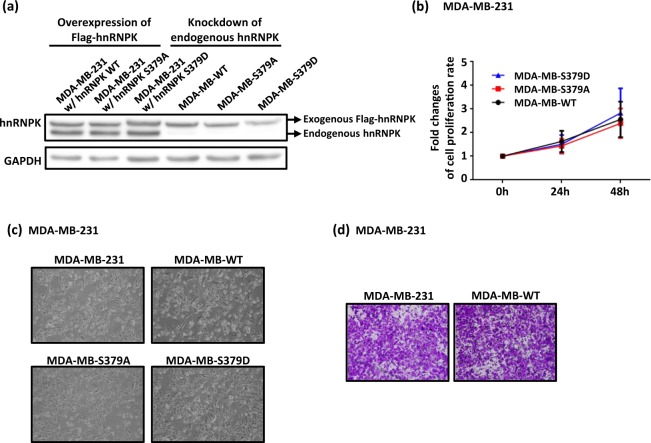
Creation of hnRNPK-engineered MDA-MB-231 cells by the replacement of endogenous hnRNPK with either S379A or S379D mutant hnRNPKs. (**a**) Establishment of MDA-MB-WT, MDA-MB-S379A and MDA-MB-S379D cell lines. MDA-MB-231 cells were first overexpressed with Flag-WT or Flag-S379A or Flag-S397D hnRNPKs respectively. Subsequent knockdown of endogenous hnRNPK in these hnRNPK-amplified cells was carried out. Expression of exogenous Flag-WT, Flag-S379A and Flag-S379D hnRNPKs in the successful knockdown clones are shown. (**b**) MDA-MB-WT, MDA-MB-S379A and MDA-MB-S379D cells exhibit similar growth rates. Comparison of proliferation among MDA-MB-WT, MDA-MB-S379A and MDA-MB-S379D cells was performed using the MTT assay at 0, 24 and 48 h after seeding. (**c**) MDA-MB-231, MDA-MB-WT, MDA-MB-S379A and MDA-MB-S379D cells exhibit similar morphology. (**d**) Parental MDA-MB-231 and MDA-MB-WT cells exhibit similar migration ability. A comparison of cell migration ability between parental MDA-MB-231 and MDA-MB-WT cells was performed. A total of 5 × 10^4^ parental MDA-MB-231 and MDA-MB-WT cells were individually seeded into Transwells for the migration assays. After 20 h, the migrated cells were fixed using 10% formaldehyde and stained with crystal violet for image analysis. The full blot images are shown in Supplementary Fig. S6.

To investigate the role of Aurora A-mediated hnRNPK S379 phosphorylation in TNBC, we intended to establish two stable clones of the MDA-MB-231 cell line in which the endogenous hnRNPK was replaced by either exogenously expressed S379A (phosphorylation-defective) or exogenously expressed S379D (phosphorylation-mimic). MDA-MB-231 cells were infected by a lentivirus which carried both shRNA-resistant and S379-mutated hnRNPKs, including the Flag-S379A hnRNPK or the Flag- S379D hnRNPK (Fig. 2a, left panel). Next, lentivirus-based shRNAs were applied to the hnRNPK mutant-expressed cells to knockdown the endogenous hnRNPK. The resulting cell lines were named as MDA-MB-WT, MDA-MB-S379A and MDA-MB-S379D cells, respectively (Fig. 2a, right panel). As shown in Fig. 2b,c, the proliferation rates and morphology among the MDA-MB-WT, MDA-MB-S379A and MDA-MB-S379D cells are very similar (Fig. 2b,c). In addition, there is no difference of migration ability between the parental MDA-MB-231 and MDA-MB-WT cells (Fig. 2d).

**Figure 3:**
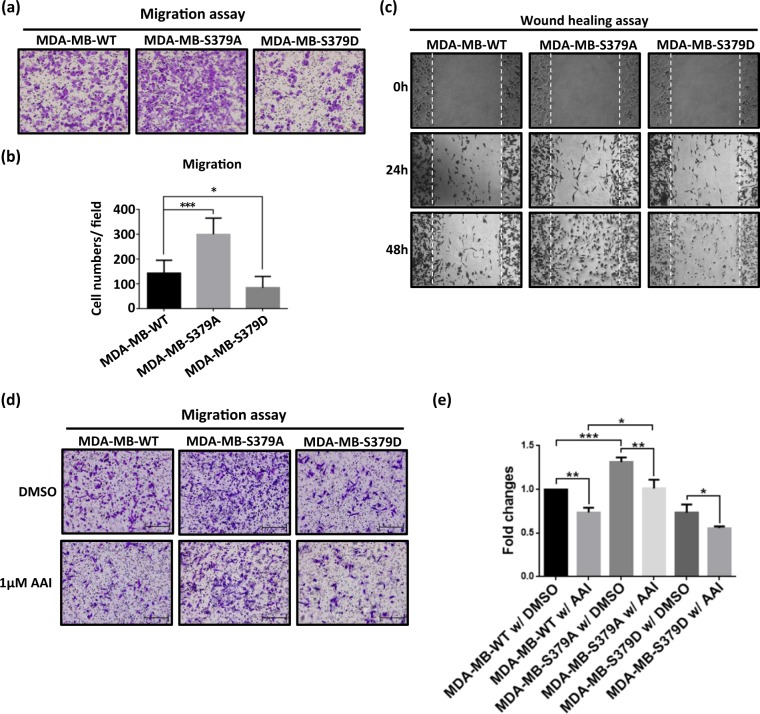
The S379A mutant hnRNPK promotes MDA-MB-231 cell migration in an Aurora-A-independent manner. (**a**) MDA-MB-S379A cells exhibit higher migration ability than the other cell lines. A comparison of cell migration ability among MDA-MB-WT, MDA-MB-S379A and MDA-MB-S379D cells was performed. A total of 3 × 10^4^ MDA-MB-WT, MDA-MB-S379A and MDA-MB-S379D cells were individually seeded into Transwells. After 20 h, the migrated cells were fixed using 10% formaldehyde and stained with crystal violet for image analysis. (**b**) Measurement of the migration abilities of MDA-MB-WT, MDA-MB-S379A and MDA-MB-S379D cells. Migrated cells in above migration assays were analyzed by ImageJ software. Bar graphs of the cell numbers per field for all three types of cells are reported. Data is shown as mean ± SD, and * indicates significant difference compared to wild type (*p < 0.05 and ***p < 0.001, n = 7). (**c**) MDA-MB-S379A cells show higher cell migration ability using a wound healing assay than either the control MDA-MB-WT cells or the MDA-MB-S379D cells. A total of 5 × 10^5^ MDA-MB-WT, MDA-MB-S379A and MDA-MB-S379D cells were independently seeded into a 6-well plate and incubated for 24 h. This was followed by scratching an area with fixed width on the plate using a 10 μL tip. The three types of cell were allowed to proliferate and migrate into the scratched area. The migrated cells were photographed using a microscope at 24 h or 48 h after the scratch was created. (**d**) Comparison of the cell migration abilities of MDA-MB-WT, MDA-MB-S379A and MDA-MB-S379D cells upon inhibition of Aurora-A. A total of 3 × 10^4^ MDA-MB-WT, MDA-MB-S379A and MDA-MB-S379D cells were independently seeded into Transwells for migration assays in the presence of DMSO (control) or of 1 μM of AAI. After 20 h, the migrated cells were fixed using 10% formaldehyde and stained with crystal violet for image analysis. (**e**) Measurement of the migration abilities of MDA-MB-WT, MDA-MB-S379A and MDA-MB-S379D cells on inhibition of Aurora-A. Bar graphs of the relative fold changes in migrated cells per field for MDA-MB-WT, MDA-MB-S379A and MDA-MB-S379D cells in the presence of DMSO (control) or AAI are reported. Data is shown as mean ± SD, and * indicates significant difference compared to wild type (*p < 0.05, n = 3).

While Aurora-A is known to promote cancer cell proliferation and metastasis^12,14,15^, hnRNPK has also been reported to support cancer cell metastasis via the regulation of the MMP family or via a TGF-β-related pathway in a diverse range of cancer types^22,40^. To clarify the influence of hnRNPK S379 phosphorylation on MDA-MB-231 cells, we compared the cell migration ability among the three cell lines. However, MDA-MB-S379A cells were found to have the highest migration ability by both migration assay (Fig. 3a,b) and wound healing assay (Fig. 3c). Notably, the MDA-MB-S379D cells exhibited the lowest migration ability, implying that Aurora-A-mediated hnRNPK S379 phosphorylation does not promote cell migration, but rather suppressed migration. We therefore hypothesized that hnRNPK S379 phosphorylation is able to regulate cell migration in an Aurora-A-independent manner. As the next step, we compared the cell migration ability of MDA-MB-WT, MDA-MB-S379A and MDA-MB-S379D cells under Aurora-A inhibition. As shown in Fig. 3d,e and Supplementary Fig. S1, MDA-MB-S379A cells still exhibited the highest migration ability even when there was suppression of Aurora-A activity. Taken together, these findings suggest that S379 phosphorylation-mimic hnRNPK (S379D) is unable to support Aurora-A-mediated promotion of migration and that a S379 phosphorylation-defective hnRNPK (S379A) conversely does promote MDA-MB-231 cell migration in an Aurora-A-independent manner.

Alternatively, to address the consequence upon coexistence of activated Aurora-A and phosphorylated hnRNPK S379, a constitutively active mutant of Aurora-A (T288D Aurora-A) was transfected into the MDA-MB-WT and MDA-MB-S379D cells to determine the total outcomes. As shown in Supplementary Fig. S2, migration of MDA-MB-S379D cells was increased upon overexpression of T288D Aurora-A, whereas such increase of migration ability is still lower than that of MDA-MB-WT cells upon overexpression of Aurora-A-T288D. This results showed that MDA-MB-S379D cells is still able to inhibit cell migration even under influence of constitutively active Aurora-A.

### hnRNPK S379 phosphorylation is inhibited by local arginine methylation in the KI region of hnRNPK

**Figure 4:**
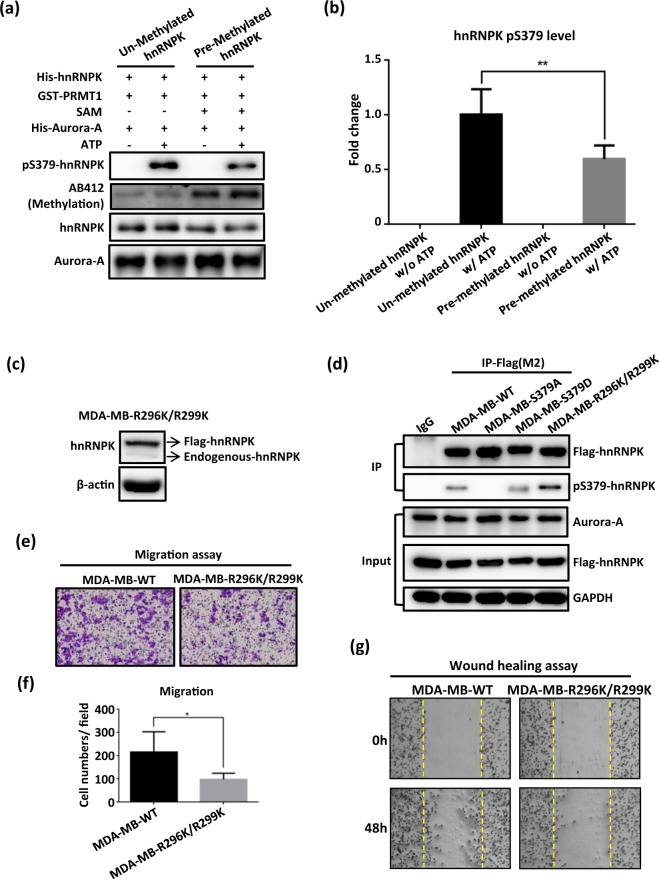
Local arginine methylation of hnRNPK inhibits S379 phosphorylation and also regulates migration of MDA-MB-231 cells. (**a**) PRMT1-mediated arginine methylation of hnRNPK inhibits the same proteins S379 phosphorylation. The His-hnRNPKs were incubated with GST-PRMT1 in the absence or presence of SAM at 30 °C for 16 h and this was followed by isolation of the resulting hnRNPKs using NTA-beads. These re-purified proteins (unmethylated and methylated hnRNPKs, respectively) were further incubated with Aurora-A in the absence or presence of ATP for 16 h as part of the *in vitro* kinase assay. The phosphorylation levels of hnRNPK S379 were detected by the use of the specific antibodies. (**b**) Quantification of *in vitro* S379 phosphorylation on hnRNPKs without or with pre-methylation. Data is shown as mean ± SD, and * indicates significant difference compared to control (**p < 0.01, n = 6). (**c**) Establishment of MDA-MB-R296K/R299K cell line. MDA-MB-231 cells were first overexpressed with an Flag-R296K/R299K hnRNPK mutant and this was followed by the knockdown of endogenous hnRNPK using shRNA. The stable clone of MDA-MB-231 cells expressing the Flag-R296K/R299K hnRNPK mutant was obtained and this was named MDA-MB-R296K/R299K. (**d**) Loss of hnRNPK Arg296/299 methylation in MDA-MB-R296K/R299K cells results in higher levels of *in vivo* S379 phosphorylation than in the control cells. Flag-tagged hnRNPKs in each stable clone were immunoprecipitated by Flag antibody and then the levels of hnRNPK S379 phosphorylation were measured using the specific antibodies. **(e**) Loss of hnRNPK Arg296/299 methylation produces lower levels of migration than by the control cells. Comparison of cell migration ability between MDA-MB-WT and MDA-MB-R296K/R299K cells was performed. (**f**) Measurement of the migration abilities of MDA-MB-WT and MDA-MB-R296K/R299K cells. Data is shown as mean ± SD, and *indicates significant difference compared to wild type (*p < 0.05, n = 5). (**g**) Determination of the migration abilities of MDA-MB-R296K/R299K and MDA-MB-WT cells in the wound healing assay. Each stable clone was independently seeded into 6-well plate for 24 h of incubation and this was followed by scratching an area on the plate using a 10 μL tip. The migrated cells were photographed at 48 h after the scratch using a microscope. The full blot images are shown in Supplementary Fig. S7.

While Aurora-A promotes the migration of MDA-MB-231 cells, the Aurora-A-induced S379 phosphorylation of hnRNPK in MDA-MB-231 cells does not promote cell migration, but rather suppresses cell migration. This suggests that hnRNPK S379 phosphorylation in MDA-MB-231 cells is not only mediated by Aurora-A, but is also co-regulated by other factors. It has been shown previously that different PTMs of the same protein may show crosstalk and cause interference with each other. Several studies have reported that arginine methylation may inhibit nearby local phosphorylation^19,41,42^. In particular, we have previously shown that R296/R299 dimethylation on hnRNPK is able to inhibit nearby S302 phosphorylation of hnRNPK, and that this subsequently inhibits cell apoptosis^26^. Thus, we next investigated whether PRMT1-mediated hnRNPK arginine methylation interferes with hnRNPK S379 phosphorylation. A recombinant hnRNPK was first pre-methylated by GST-PRMT1 using an *in vitro* methylation assay and, subsequently, the level of arginine methylation was examined by Western blotting using arginine methylation-specific antibodies. Both the pre-methylated and un-methylated hnRNPKs were then subjected to the *in vitro* kinase reaction. The measurement of the S379 phosphorylation levels was conducted by Western blotting using the S379 phosphorylation-specific polyclonal antibodies (Fig. 4a,b). The results showed that the level of S379 phosphorylation of the pre-methylated hnRNPK was lower than that of un-methylated hnRNPK, which suggests that PRMT-1-mediated arginine methylation of hnRNPK indeed does suppress the local S379 phosphorylation mediated by Aurora-A. We have previously shown that PRMT-1 preferentially and effectively methylated hnRNPK at R296 and R299, while there may also be moderate methylation of R256, R258, and R268 during this PRMT1-mediated methylation^26,27^. To determine whether crosstalk between PRMT1-mediated arginine methylation and Aurora-A-mediated phosphorylation does occur in MDA-MB-231 cells, we next established a stable clone of the MDA-MB-231 cell line, the endogenous hnRNPK of which was replaced by a R296K/R299K double-mutated hnRNPK; this was named MDA-MB-R296K/R299K (Fig. 4c). The hnRNPK present in MDA-MB-R296K/R299K or MDA-MB-WT cells was then separately immunoprecipitated using a Flag-M2 antibody, and the levels of phosphorylation at S379 compared. As shown in Fig. 4d, hnRNPK S379 phosphorylation in MDA-MB-R296K/R299K cells is significantly higher than in MDA-MB-WT cells. This suggests that hnRNPK S379 phosphorylation is negatively regulated by PRMT1-mediated R296/R299 dimethylation of hnRNPK. In addition, we further investigated whether S379 phosphorylation of hnRNPK affects its own methylation status, and our results showed that S379 phosphorylation of hnRNPK did not vice versa affect the methylation level of hnRNPK in MDA-MB-231 cells (Supplementary Fig. S3). Next we determined the migration ability of the MDA-MB-R296K/R299K cells. As shown in Fig. 4e–g, the migration ability of the MDA-MB-R296K/R299K cells was indeed found to be lower than that of MDA-MB-WT cells, and to be similar to that found in MDA-MB-S379D cells. Taken together, our findings support the idea that hnRNPK arginine methylation is involved in regulating the migration ability of MDA-MB-231 cells and this occurs by the limiting of its own S379 phosphorylation level.

### S379-hypophosphorylated hnRNPK seems to promote cell migration via the β-catenin-MMP pathway

**Figure 5:**
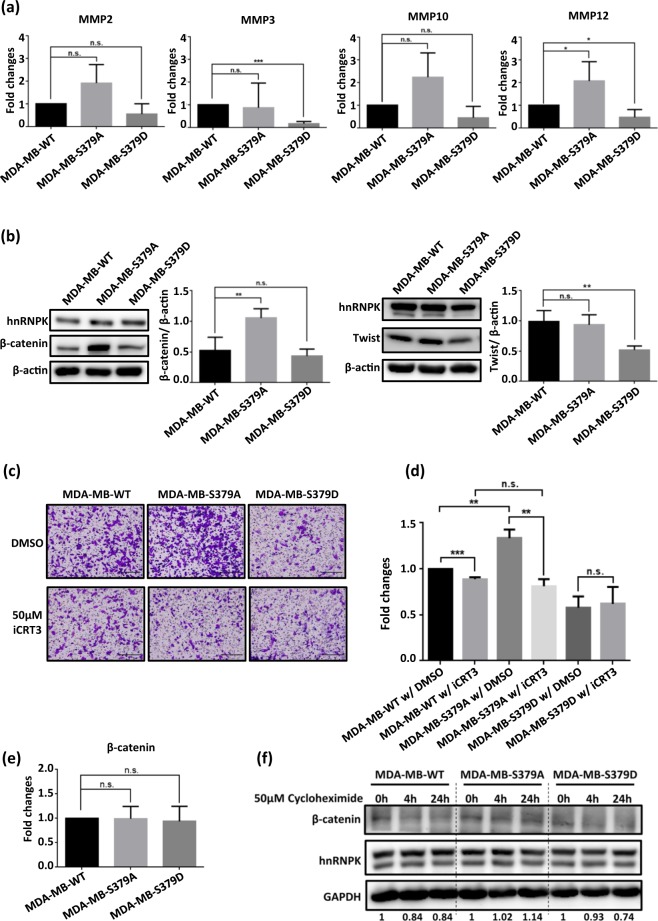
Comparison of the MMP and EMT markers between MDA-MB-WT, MDA-MB-S379A and MDA-MB-S379D cells. (**a**) In comparison to control cells, MDA-MB-S379A cells showed significantly higher transcript levels of MMP12, while MDA-MB-S379D cells showed significantly lower transcript levels of MMP3. The transcript levels of MMP members across each stable clone were compared using qPCR. Fold changes in these MMPs were calculated and are shown in the bar graphs with statistics. Data is shown as mean ± SD, and *indicates significant difference compared to wild type (*p < 0.05, ***p < 0.001) (n > 3). (**b**) MDA-MB-S379A cells exhibit significantly higher levels of β-catenin and MDA-MB-S379D cells exhibited significantly lower levels of Twist than the control cells. The protein levels of β-catenin and Twist across each stable clone were measured using Western blot analysis. Quantification of the Western blot results is presented in the bar graphs with statistics. Data is shown as mean ± SD, and * indicates significant difference compared to wild type (**p < 0.01, n = 4). (**c**) β-catenin activity is closely correlated with S379A hnRNPK-mediated cell migration. Cells were seeded into Transwells for migration assays in the presence of DMSO (control) or in the presence of 50 μM of the β-catenin inhibitor, iCRT3. After 20 h, the migrated cells were fixed and stained for image analysis. (**d**) Measurement of the migration abilities across MDA-MB-WT, MDA-MB-S379A and MDA-MB-S379D cells upon inhibition of β-catenin activity. Fold changes in migration suppression upon iCRT3 treatment were calculated and are shown as bar graphs with statistics. Data is shown as mean ± SD, and *indicates significant difference compared to wild type (***p < 0.001, n = 4). (**e**) The mRNA level of β-catenin is not affected by hnRNPK S379 phosphorylation. The transcript levels of β-catenin across each stable clone were measured using qPCR. Fold changes of β-catenin mRNA were calculated based on the value for the MDA-MB-WT cells and are presented as bar graphs with statistics. Data is shown as mean ± SD (n = 4). (**f**) MDA-MB-S379A cells show greater β-catenin stability than the control cells. Each stable clones were incubated with 50 μM cyclohexamide for indicated times and this was followed by measurement of their β-catenin levels. The full blot images are shown in Supplementary Fig. S8.

**Figure 6:**
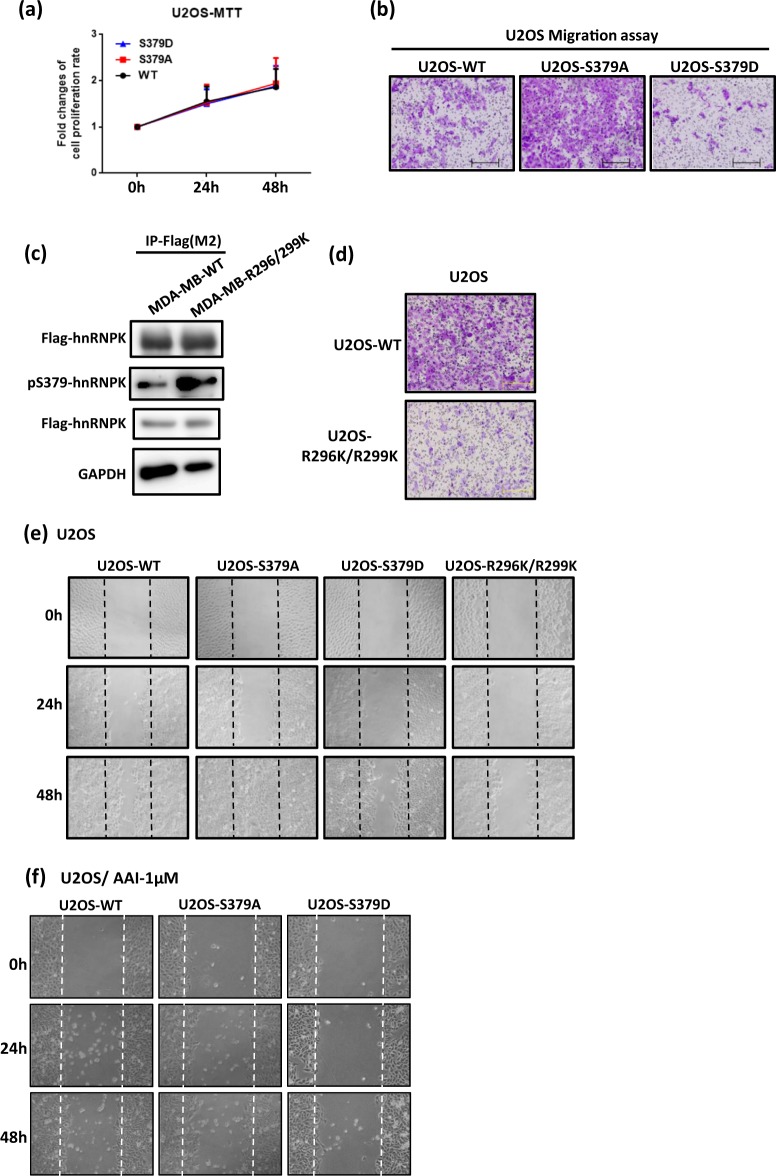
The S379A hnRNPK mutant exhibits similar migration-promoting ability in the bone tumor U2OS cell. (**a**) U2OS-WT, U2OS-S379A and U2OS-S379D cells exhibit similar growth rates. Comparison of proliferation among each stable clone was performed using the MTT assay at 0, 24 and 48 h after seeding. (**b**) U2OS-S379A cells exhibit higher migration ability than either U2OS-WT or U2OS-S379D cells by the Transwell migration assay. A total of 3 × 10^4^ of each stable clone were independently seeded into Transwells for the migration assay. After 20 h, the migrated cells were fixed and stained for image analysis. (**c**) Loss of hnRNPK Arg296/299 methylation in U2OS-R296K/R299K cells results in higher levels of *in vivo* S379 phosphorylation than in the control U2OS-WT cells. Flag-tagged hnRNPK in the U2OS-WT and U2OS-R296K/R299K cells were immunoprecipitated by anti-Flag antibody. The levels of hnRNPK S379 phosphorylation were measured using the specific antibodies. (**d**) U2OS-R296K/R299K cells exhibit a lower migration ability in the Transwell migration assay than do the control U2OS-WT cells. Cells were independently seeded into Transwells for the migration assay. After 20 h, the migrated cells were fixed and stained for image analysis. (**e**) U2OS-S379A cells exhibit higher cell migration ability in wound healing assay than either the control U2OS cells or the U2OS-S379D cells. Each stable clones were independently seeded into a 6-well plate for 24 h incubation and this was followed by scratching a fixed area on the plate using a 10 μL tip. The migration of cells into the wound area were photographed by microscope at 24 or 48 h after scratching. (**f**) U2OS-S379A cells exhibit higher cell migration ability in wound healing assay than either the control U2OS-WT cells or the U2OS-S379D cells upon inhibition of Aurora-A. Each stable clone was independently seeded into a 6-well plate for incubation in plate for 24 h. After 24 h incubation, the medium was changed into the buffer with either DMSO (control) or 1 μM of AAI, and followed by scratching a fixed area on the plate using a 10 μL tip. The migration of cells into the wound area were photographed by microscope at 24 or 48 h after scratching. The full blot images are shown in Supplementary Fig. S9.

We next investigated the potential migration mechanism that is regulated by hnRNPK S379 phosphorylation. It has been shown that hnRNPK promotes cell migration via the upregulation of transcription of members of the MMP family, including MMP2, MMP3, MMP10 and MMP12^21,22,43^. For example, hnRNPK is able to activate Erk to increase MMP3 and MMP10 levels in the immortalized NIH3T3 cells^21^. Alternatively, it has been shown to be able to directly bind to the promoter region of MMP12 and regulate its transcription in nasopharyngeal carcinoma^43^. We examined the mRNA levels of MMP2, MMP3, MMP10 and MMP12 across the MDA-MB-WT, MDA-MB-S379A and MDA-MB-S379D cell lines. As shown in Fig. 5a, the level of MMP3 was significantly decreased in the MDA-MB-S379D cells, which have a low migration rate, while the level of MMP12 was significantly increased in MDA-MB-S379A cells, which have a high migration rate. In spite of the fact that the levels of MMP2 and MMP10 were also increased in the MDA-MB-S379A cells, these changes were not statistically significant. In addition, we also observed that two EMT markers, β-catenin and Twist, showed differential expression when MDA-MB-WT, MDA-MB-S379A and MDA-MB-S379D cells were compared, as shown in Fig. 5b. β-catenin, an upstream regulator of MMP12, was more abundant in MDA-MB-S379A cells than in MDA-MB-WT cells. On the other hand, Twist, an upstream regulator of MMP3^44^, is less abundant in MDA-MB-S379D cells than in MDA-MB-WT cells (Fig. 5b). Furthermore, when we compared the cell migration ability among the MDA-MB-WT, MDA-MB-S379A and MDA-MB-S379D cell lines after treatment with a β-catenin inhibitor, iCRT3, it was found, as shown in Fig. 5c,d and Supplementary Fig. S4, that the migration ability of MDA-MB-S379A cells upon β-catenin inhibition was reduced to levels similar to those of MDA-MB-WT and MDA-MB-S379D cell. This implies that the S379A hnRNPK mutant promotes migration in a β-catenin-dependent manner. We next investigated how the S379A hnRNPK mutant induces high level of expression of β-catenin in MDA-MB-S379A cells. As shown in Fig. 5e, the RNA levels of β-catenin were similar when the MDA-MB-WT, MDA-MB-S379A and MDA-MB-S379D cell lines were compared. However, the MDA-MB-S379A cells were found to have the longest half-life of β-catenin across these three cell lines (Fig. 5f), which suggests that loss of hnRNPK S379 phosphorylation in S379A hnRNPK mutant promotes cell migration via an increase in the protein stability of β-catenin. In this context, it should be noted that the S379A and S379D mutations of hnRNPK in U2OS osteosarcoma cells have also been found to have the same influence on cell migration (Fig. 6a–f). Taking the above findings together, we propose that the S379site in hnRNPK is involved in regulating cancer cell migration and, based on the results obtained using the mutants S379A and S379D, this occurs via distinct pathways. According to our findings, a hypo-S379-phosphorylated hnRNPK in MDA-MB-231 cells is able to promote cell migration via the hnRNPK-β-catenin-MMP12 axis, while, conversely, a hyper-S379-phosphorylated hnRNPK is able to suppress cell migration via the hnRNPK-Twist-MMP3 axis.

## Discussion

TNBC is a subtype of breast cancer with high malignancy and strong metastatic ability; it causes higher levels of mortality among TNBC patients than breast cancer among non-TNBC patients^45^. Research into the metastasis mechanisms used by TNBC is needed in order to develop novel strategies to treat this disease. As shown by previous studies and by analysis of information from the CCLE (Cancer Cell Line Encyclopedia), amplification of Aurora-A kinase has been widely observed in breast cancers^16,17^. Aurora-A is known to promote proliferation of TNBC cells and metastasis by TNBC cells, and has been proposed as a putative therapeutic target for breast cancer^17,46^. Previously, we demonstrated that serine 379 phosphorylation of hnRNPK is mediated by Aurora-A kinase *in vitro* and *in vivo* (Fig. 1a,b)^35^. However, the cellular function(s) of such hnRNPK S379 phosphorylation remains unknown. Based on the role that Aurora-A plays in promoting breast cancer, we herein have investigated the cellular function(s) of hnRNPK S379 phosphorylation in MDA-MB-231 cells by establishing several stable clones in which the endogenous hnRNPK gene has been replaced by either a S379 phosphorylation-defective hnRNPK gene (S379A) or a S379 phosphorylation-mimic hnRNPK gene (S379D) (Fig. 2a). Our results indicate that cell migration by MDA-MB-231 cells is influenced by the degree of S379 phosphorylation, and, at the same time, the above mutations do not affect cell proliferation and cell morphology (Fig. 2b,c). Unexpectedly, Aurora-A-mediated S379 phosphorylation of hnRNPK did not parallel the migration-promoting role of Aurora-A, but rather it suppressed cell migration, as demonstrated by the greater migration potential of MDA-MB-S379A cells compared to either MDA-MB-WT cells or MDA-MB-S379D cells (Fig. 3a–c). In addition, the enhanced migration of MDA-MB-S379A cells persisted even when Aurora-A activity was suppressed (Fig. 3d,e). These results suggest the possibility that hnRNPK S379 phosphorylation is involved in the regulation of cancer cell migration in an Aurora-A-independent manner.

In addition to the above, we have also shown that S379 phosphorylation of hnRNPK is inhibited by R296/R299 dimethylation *in vitro* of the same protein (Fig. 4a,b), whereas loss of such methylation on hnRNPK did increase the S379 phosphorylation level of hnRNPK *in vivo* (Fig. 4d). Notably, MDA-MB-231 cells carrying either R296K/R299K methylation-defective or S379D phosphorylation-mimic hnRNPK showed lower cell migration when compared to MDA-MB-WT cells (Fig. 4c,e–g). These results suggest the possibility that there is crosstalk between arginine methylation and serine phosphorylation in hnRNPK and that this may be responsible for the hnRNPK-mediated regulation of migration in MDA-MB-231 cells, which we have shown is independent of the migration-promoting function of Aurora-A.

We have previously reported that arginine methylation of hnRNPK is able to switch between a pro-apoptotic role and an anti-apoptotic role^26^. Likewise, we herein have presented a similar phenomenon where by arginine methylation permits hnRNPK to play a similar dual role regarding the regulation of cancer cell migration. This suggests that there is a need to explore further whether the migration-promoting ability of hnRNPK and migration-diminishing ability of hnRNPK exert their distinct functions via different mechanism.

Next, two well-known EMT markers were examined to determine whether they are involved in the hnRNPK S379 phosphorylation-mediated suppression of cell migration. Notably, the S379A and S379D hnRNPKs were found to regulate cell migration by influencing the expression of different EMT markers. As Fig. 5f shows, the half-life of β-catenin protein is longer in MDA-MB-S379A cells than in either MDA-MB-WT or MDA-MB-S379D cells. Consequently, levels of β-catenin and its downstream protein MMP12 are both increased in MDA-MB-S379A cells (Fig. 5a,b). It has been reported that hnRNPK is able to induce MMP12 expression and activity in nasopharyngeal carcinoma via binding to the MMP12 promoter^43^ and our results suggest a modification-dependent process involving the transcriptional activity of hnRNPK. On the other hand, a decrease in the transcription of MMP12 was also observed in MDA-MB-S379D cells, but this was not associated with a significant change in the level of β-catenin. One possibility is that S379D hnRNPK inhibits MMP12 expression in a β-catenin-independent manner, and this will need further investigation in order to discover how suppression of MMP12 expression is brought about by S379D hnRNPK.

Twist, a transcription factor that is often overexpressed in breast cancer cells, has been shown to activate the transcription of various mesenchymal markers that have the ability to promote metastasis and, consequently, the level of Twist is often positively correlated with migration of breast cancer cells^47,48^. In our findings, the levels of Twist and its downstream MMP3 transcripts were found to be decreased in MDA-MB-S379D cells, whereas the changes in MMP2 and MMP10 transcripts levels were not significant in MDA-MB-S379D cells (Fig. 5a,b). This suggests that the S379D hnRNPK mutant may be able to inhibit cell migration via a decrease in the expression level of Twist. We thus hypothesize that the S379A and S379D hnRNPK mutants may influence cell migration via a range of distinct interactions with different protein target partners in order to regulate the levels of β-catenin and Twist.

It is well known that hnRNPK interacts with diverse protein and DNA/RNA targets via its KI region, as well as via the three KH domains. Furthermore, hnRNPK S379 phosphorylation is located near the KH3 domain; nevertheless, whether S379 phosphorylation is able to affect the interaction between hnRNPK and DNA targets or hnRNPK and RNA targets remains unknown. Recently, hnRNPK has been reported to interact with several lncRNAs and to regulate their functions in cancer. For example, hnRNPK has been reported to interact with treRNA to enhance breast cancer metastasis^49^. Furthermore, hnRNPK is known to interact with linc00460 to promote lung cancer cell metastasis^50^. Finally, hnRNPK interacts with the MYU lncRNA, a c-Myc target that is overexpressed in colon cancer, to increase CDK6 transcription, as well as to promote the G1-S cell cycle transition during colon cancer, both of which are driven by Wnt/c-Myc signaling^51^. Whether S379 phosphorylation regulates the interaction between hnRNPK and DNA/RNA thus also is in needs of further extensive study.

**Figure 7:**
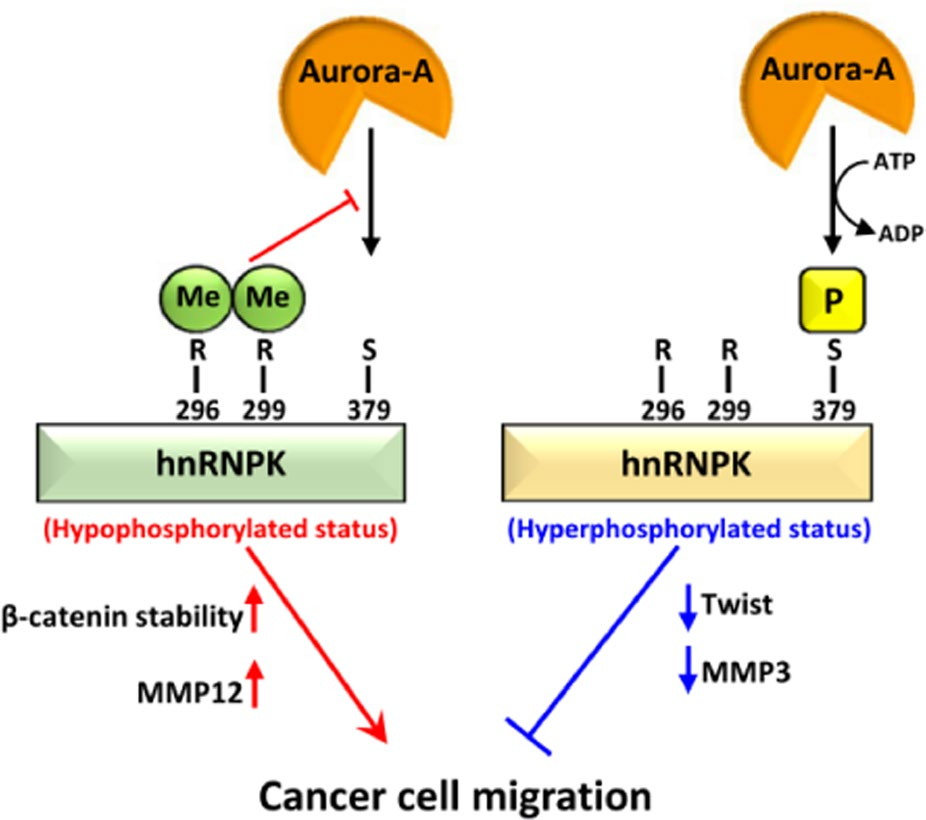
hnRNPK plays a dual role upon amplification of Aurora-A activity in MDA-MB-231 cells.

In summary, our study is the first to demonstrate the cellular function of Aurora-A-mediated hnRNPK S379 phosphorylation in TNBC cells. In addition, this phosphorylation was found to be negatively regulated by local arginine methylation of hnRNPK. Moreover, suppression of such phosphorylation by the R296/R299 dimethylation of hnRNPK in MDA-MB-231 cells is able to maintain hnRNPK in a hypo-S379-phosphorylated status that favors the promotion of migration by MDA-MB-231 cells via an Aurora-A-independent pathway. We therefore suggest that hnRNPK plays dual roles in relation to the amplification of Aurora-A in cells (Fig. 7). When hnRNPK is highly methylated and thus the protein is being maintained in a hypo-S379-phosphorylated state, it promotes cell migration via an increase in β-catenin and MMP12, both in the presence of and in the absence of Aurora-A activity. Alternatively, hyper-S379-phosphorylated hnRNPK, which is created by Aurora-A kinase in the absence of R296/R299 dimethylation, initiates an anti-migration mechanism by suppressing Twist and MMP3; this is in spite of any increase in Aurora-A activity. These mechanisms may increase the flexibility of cancer cells and allow these cells to adjust their degree of migration to their environment. Our present study, in terms of therapy, provides a possible anti-migration strategy that involves inhibiting the PMRT-mediated arginine methylation of hnRNPK or the targeting of the hnRNPK S379 phosphorylation-specific phosphatase.

## Supplementary information

SUPPLEMENTARY INFO
