# Supplementary material for: hnRNPK S379 phosphorylation participates in migration regulation of triple negative MDA-MB-231 cells
Source: Sci Rep. 2019 May 20;9:7611. doi: 10.1038/s41598-019-44063-z (PMC6527834; doi:10.1038/s41598-019-44063-z)
Supplement: Supplementary file 1 — SUPPLEMENTARY INFO [file 41598_2019_44063_MOESM1_ESM.pdf]

Supplementary Figures

hnRNPK S379 phosphorylation participates in migration regulation of triple negative MDA-MB-231 cells

Hsin-Yu Tsai[1], Shu-Ling Fu[2] and Chao-Hsiung Lin* [1,3,4,5]

[1]Department of Life Sciences and Institute of Genome Sciences, [2]Institute of Traditional Medicine, [3]Institute of Biopharmaceutical Sciences, and [4]Proteomics Research Center, and [5]Aging and Health Research Center, National Yang-Ming University, Taipei, Taiwan ROC

* Corresponding author

(a)

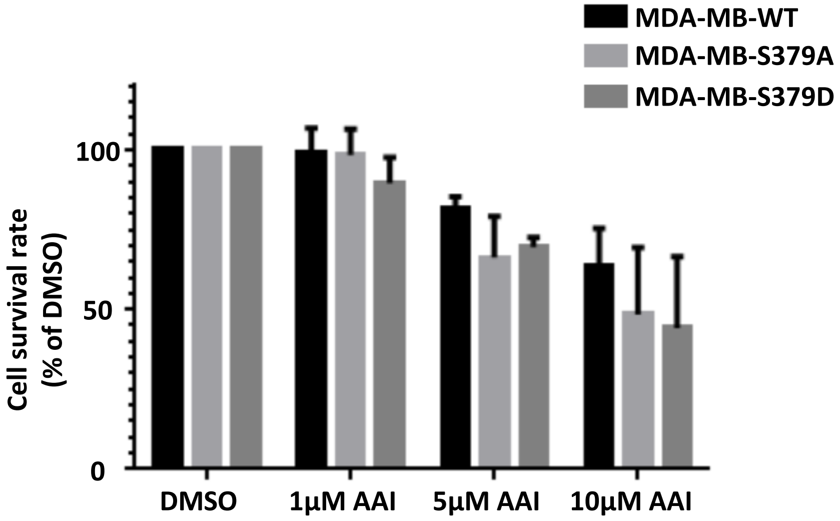

(b)

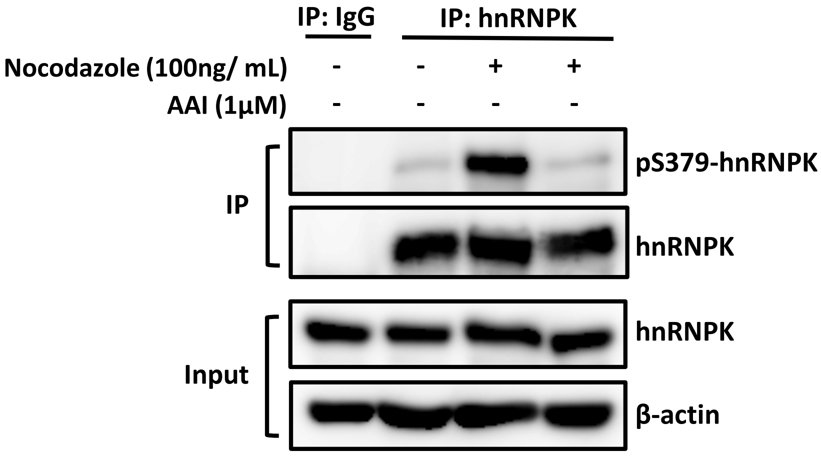

Supplementary Figure 1. Comparison of the cell survival rate and hnRNPK S379 phosphorylation across MDA-MB-WT, MDA-MB-S379A and MDA-MB-S379D cells upon inhibition of Aurora-A activity. (a) MDA-MB-WT, MDA-MB-S379A and MDA-MB-S379D cells exhibit similar cell survival rates after treatment with DMSO or 1, 5, 10μM of AAI treatment. MDA-MB-WT, MDA-MB-S379A and MDA-MB-S379D cells were respectively cultured in the present of DMSO only, or in the presence of 1μM, 5μM or 10μM of AAI for 20h. MTT assays were used to determine the cell survival rates of all three groups. **(b) S379 phosphorylation of hnRNPK is elevated by nocodazole treatment in MDA-MD-23 cells and this elevation is able to be suppressed by inhibition of Aurora-A activity.** MDA-MB-231 cells were cultured with DMSO or nocodazole in the presence or absence of AAI (1μM) for 20h. The hnRNPK in each sample was immunoprecipitated by anti-hnRNPK antibodies. The levels of hnRNPK S379 phosphorylation were then determined by Western blot analysis using the S379 phosphorylation-specific antibodies.

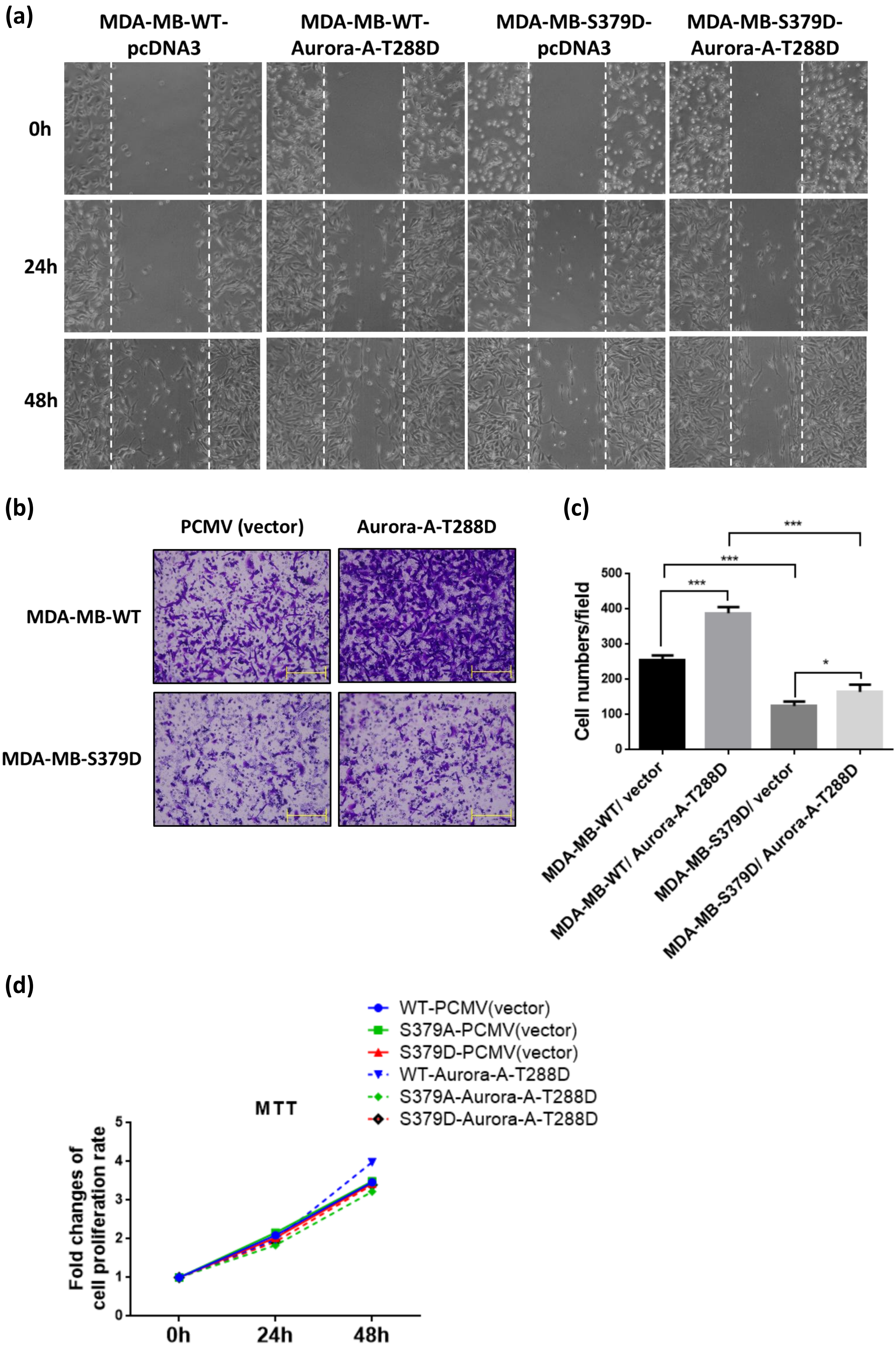
(a)
MDA-MB-WT-pcDNA3
MDA-MB-WT-Aurora-A-T288D
MDA-MB-S379D-pcDNA3
MDA-MB-S379D-Aurora-A-T288D
0h
24h
48h
(b)
PCMV (vector)
Aurora-A-T288D
MDA-MB-WT
MDA-MB-S379D
(c)
Cell numbers/field
500
400
300
200
100
0
***
***
***
*
MDA-MB-WT/ vector
MDA-MB-WT/ Aurora-A-T288D
MDA-MB-S379D/ vector
MDA-MB-S379D/ Aurora-A-T288D
(d)
WT-PCMV(vector)
S379A-PCMV(vector)
S379D-PCMV(vector)
WT-Aurora-A-T288D
S379A-Aurora-A-T288D
S379D-Aurora-A-T288D
MTT
Fold changes of cell proliferation rate
5
4
3
2
1
0
0h
24h
48h

Supplementary Figure 2. MDA-MB-S379D cells overexpressed with T288D Aurora-A exhibit elevated migration ability but not increased proliferation. (a) Comparison of wound healing ability of MDA-MB-WT and MDA-MB-S379D cells upon T288D Aurora-A-transfection. A total of $5x10^5$ T288D Aurora-A-transfected MDA-MB-WT and MDA-MB-S379D cells were independently seeded into a 6-well plate and incubated for 24h. This was followed by scratching an area with fixed width on the plate using a 10μL tip. The two types of cell were allowed to migrate into the scratched area. The migrated cells were photographed using a microscope at 24h or 48h after the scratch was created. **(b) Comparison of migration ability of MDA-MB-WT and MDA-MB-S379D cells upon T288D Aurora-A-transfection.** A total of 3 X 10^5 T288D Aurora-A-transfected MDA-MB-WT and MDA-MB-S379D cells were seeded into trans-wells for invasion assay for 20h. **(c)** The quantitative result for (b). **(d) Comparison of proliferation rates of MDA-MB-WT and MDA-MB-S379D cells upon T288D Aurora-A-transfection.** Measurement of proliferation rates was performed using the MTT assay at 0, 24 and 48h after seeding.

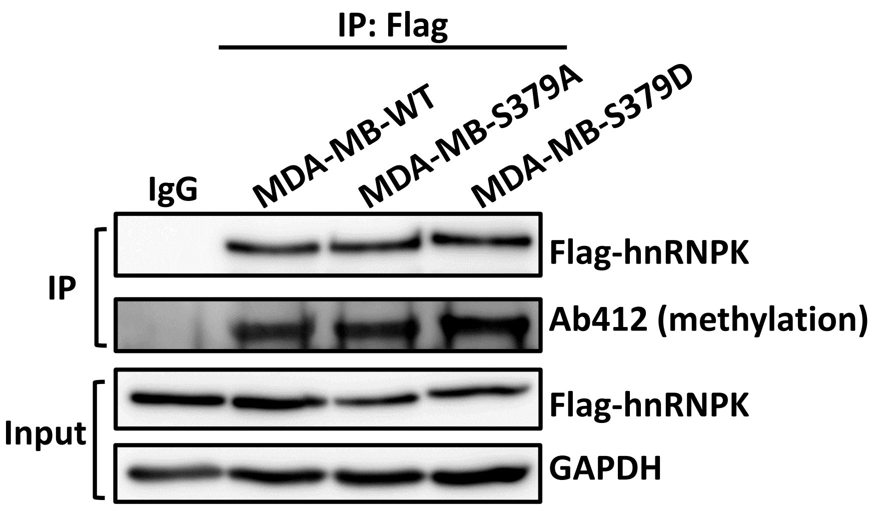

Supplementary Figure 3. S379 phosphorylation of hnRNPK does not affect the methylation level of hnRNPK in MDA-MB-231 cells. All cellular hnRNPKs from the MDA-MB-WT, MDA-MB-S379A and MDA-MB-S3979D cells was respectively precipitated for the detection of their arginine methylation levels using anti-arginine-methylation antibody (AB412). The methylation levels of hnRNPKs are nearly the same among all three cells.

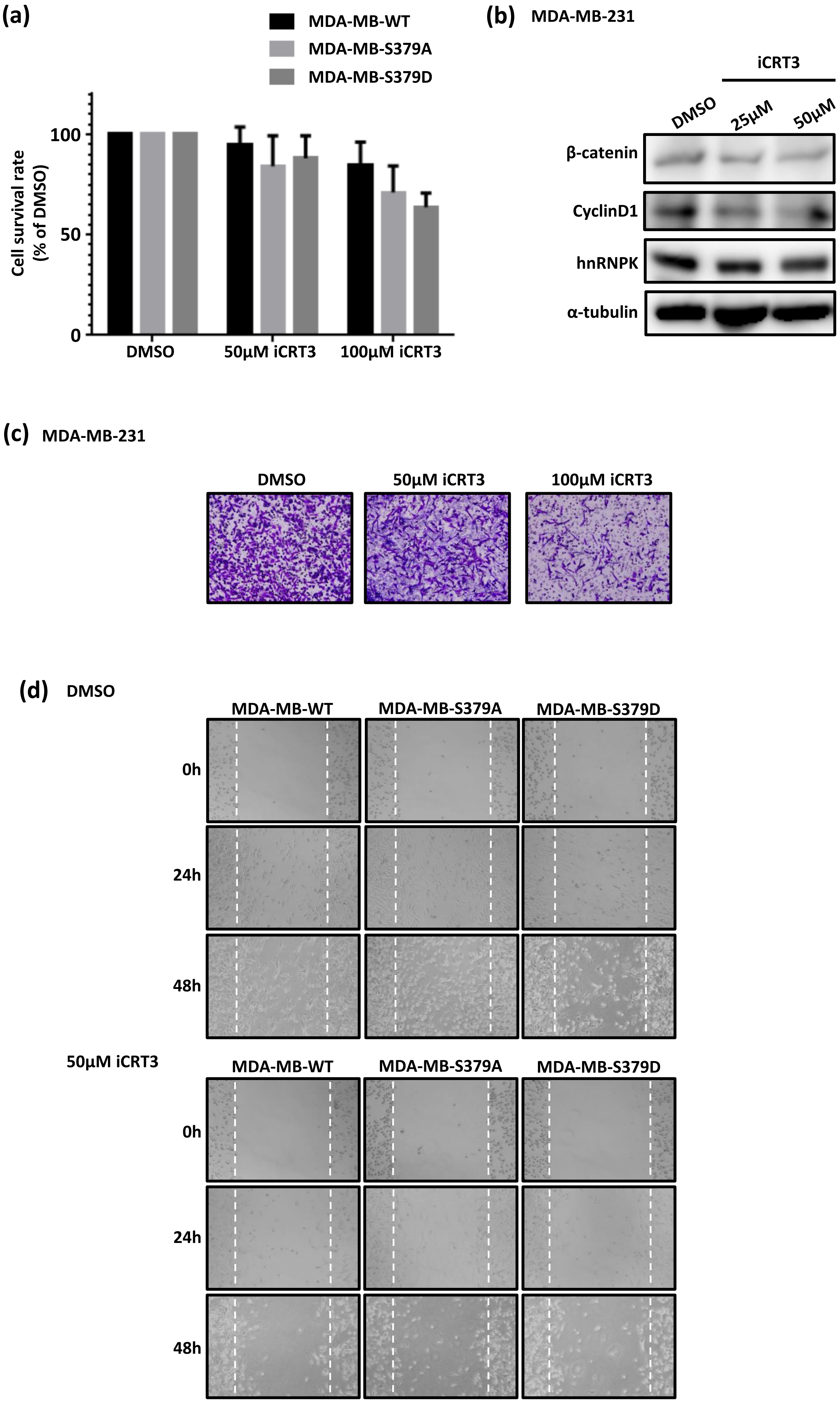
(a)
MDA-MB-WT
MDA-MB-S379A
MDA-MB-S379D
Cell survival rate
(% of DMSO)
100
50
0
DMSO
50µM iCRT3
100µM iCRT3
(b) MDA-MB-231
iCRT3
DMSO
25µM
50µM
β-catenin
CyclinD1
hnRNPK
α-tubulin
(c) MDA-MB-231
DMSO
50µM iCRT3
100µM iCRT3
(d) DMSO
MDA-MB-WT
MDA-MB-S379A
MDA-MB-S379D
0h
24h
48h
50µM iCRT3
MDA-MB-WT
MDA-MB-S379A
MDA-MB-S379D
0h
24h
48h

Supplementary Figure 4. Inhibition of β-catenin transcription activity suppresses the migration of MDA-MB-WT, MDA-MB-S379A and MDA-MB-S379D cells. (a) MDA-MB-WT, MDA-MB-S379A and MDA-MB-S379D cells exhibit similar survival rates upon iCRT3 treatment. The cell survival rates of MDA-MB-WT, MDA-MB-S379A and MDA-MB-S379D cells upon DMSO treatment (control) or iCRT3 treatment (50µM or 100µM) were measured using the MTT assay. Bar graphs of the relative fold changes in cell survival rates for the MDA-MB-WT, MDA-MB-S379A and MDA-MB-S379D cells upon DMSO (control) or iCRT3 are shown. **(b) Protein levels of Cyclin D1, a transcriptional target of β-catenin, are decreased upon iCRT3 treatment using MDA-MB-231 cells.** MDA-MB-231 cells were independently treated with DMSO, 25µM or 50µM iCRT3 for 20h. The protein levels of Cyclin D1 in all three groups were determined using Western blot analysis. **(c) Migration ability of MDA-MB-231 cells was suppressed by inhibition of β-catenin transcriptional activity.** A total of $3x10^4$ MDA-MB-231 cells were seeded into Transwells for the migration assay under DMSO (control) or 50µM/100µM of iCRT3. After 20h, the migrated cells were fixed using 10% formaldehyde and stained with crystal violet for image analysis. **(d) MDA-MB-WT, MDA-MB-S379A and MDA-MB-S379D cells, upon inhibition of β-catenin transcription activity, exhibit similar migration abilities using the wound healing assay.** A total of $5x10^5$ MDA-MB-WT, MDA-MB-S379A and MDA-MB-S379D cells were independently seeded into 6-well plates for 24h under DMSO (control) or 50µM iCRT3 treatment and this was followed by scratching out an area of ah fixed width on the plate using a 10µL tip. The three cell types were allowed to proliferate and migrated into the scratched area. The migrated cells were photographed at 48h after the scratch using a microscope.

For Figure 1a

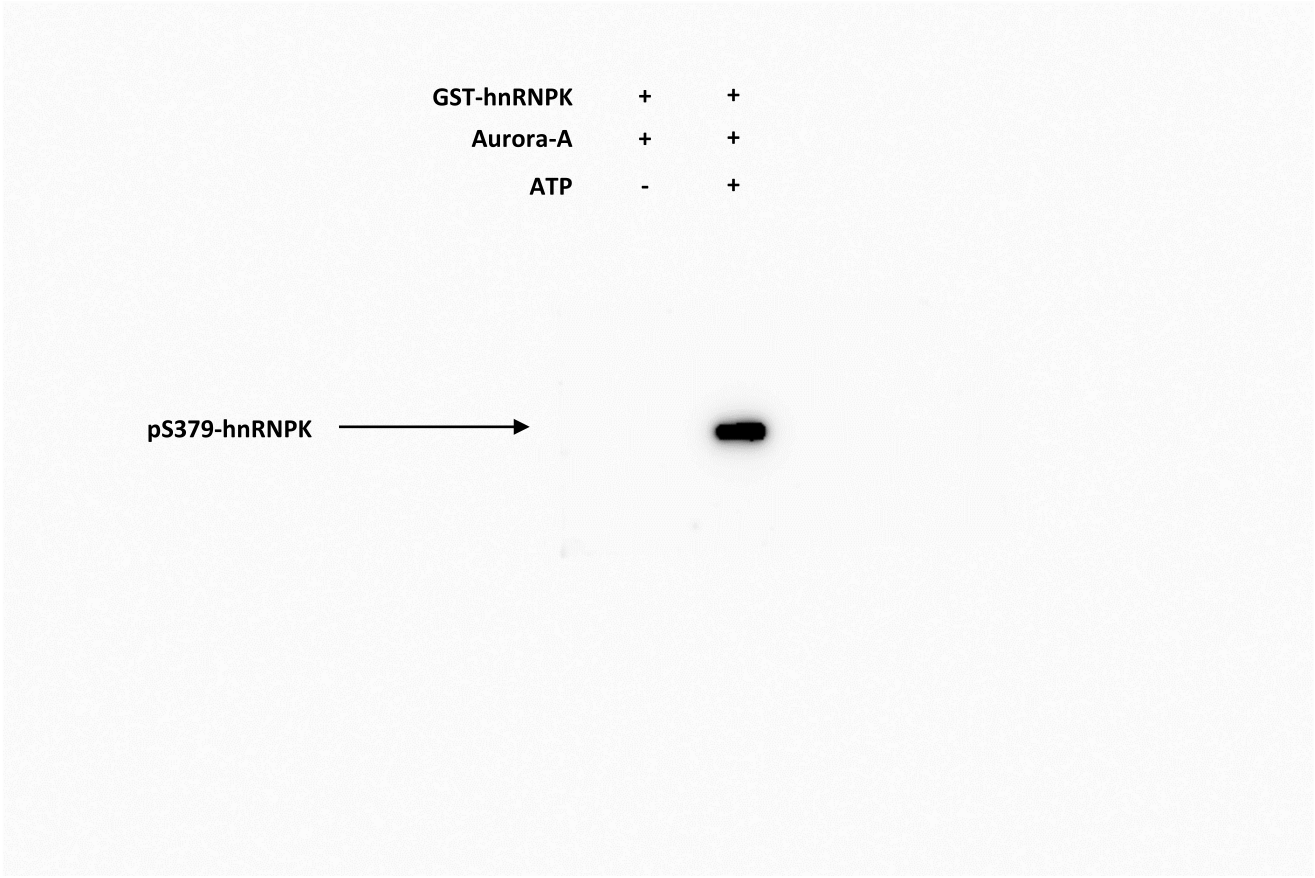

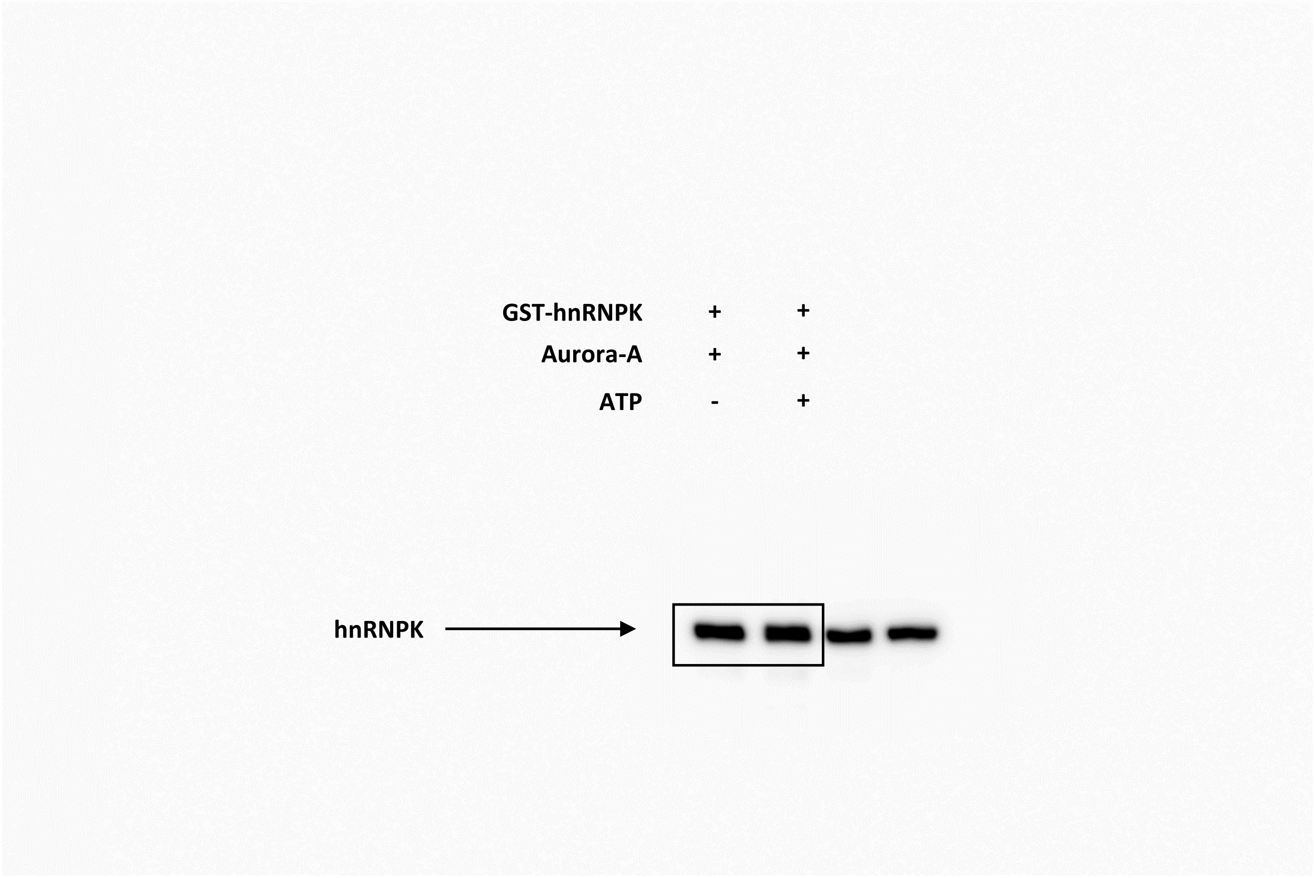

Supplementary figure 5. Raw images of the cropped blots presented in the main figure 1.

For Figure 1a

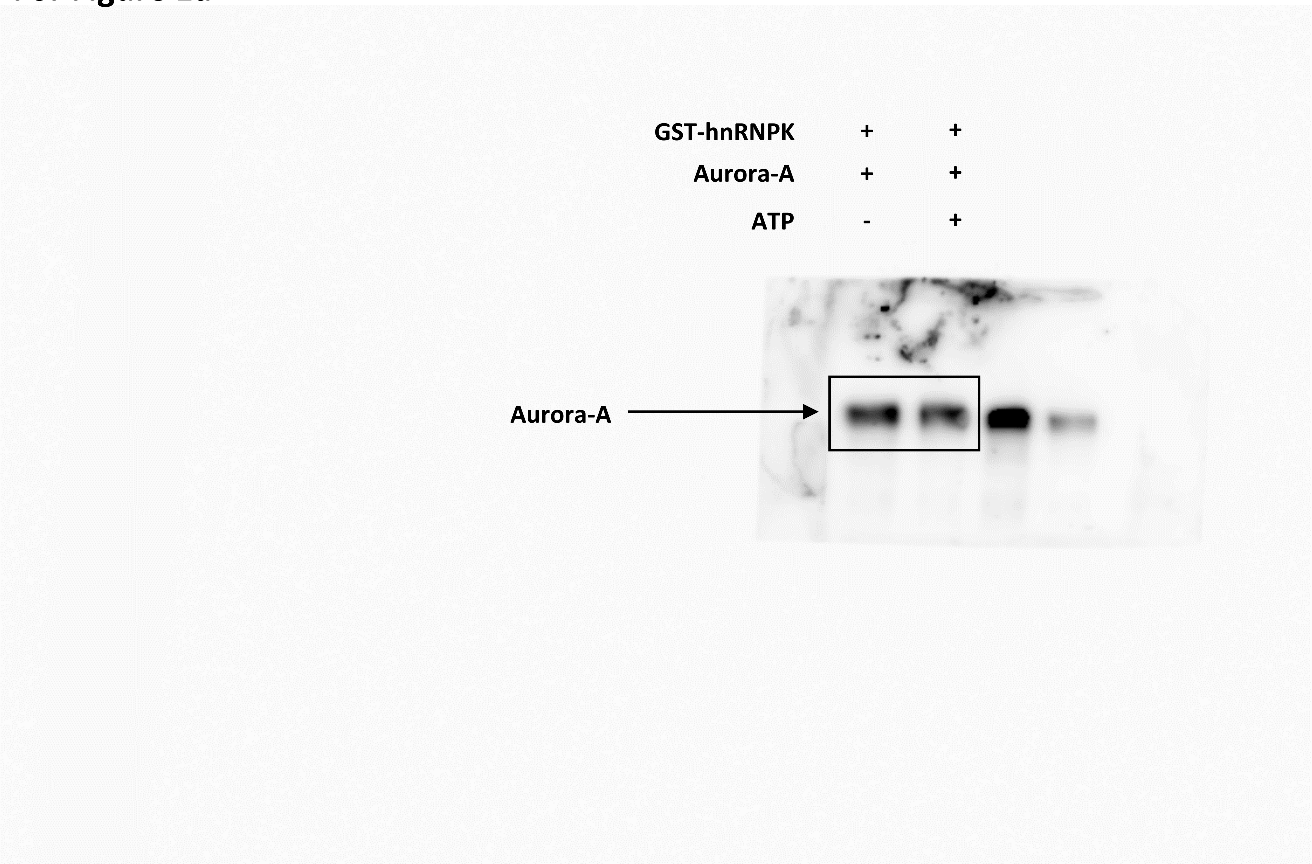

For Figure 1b

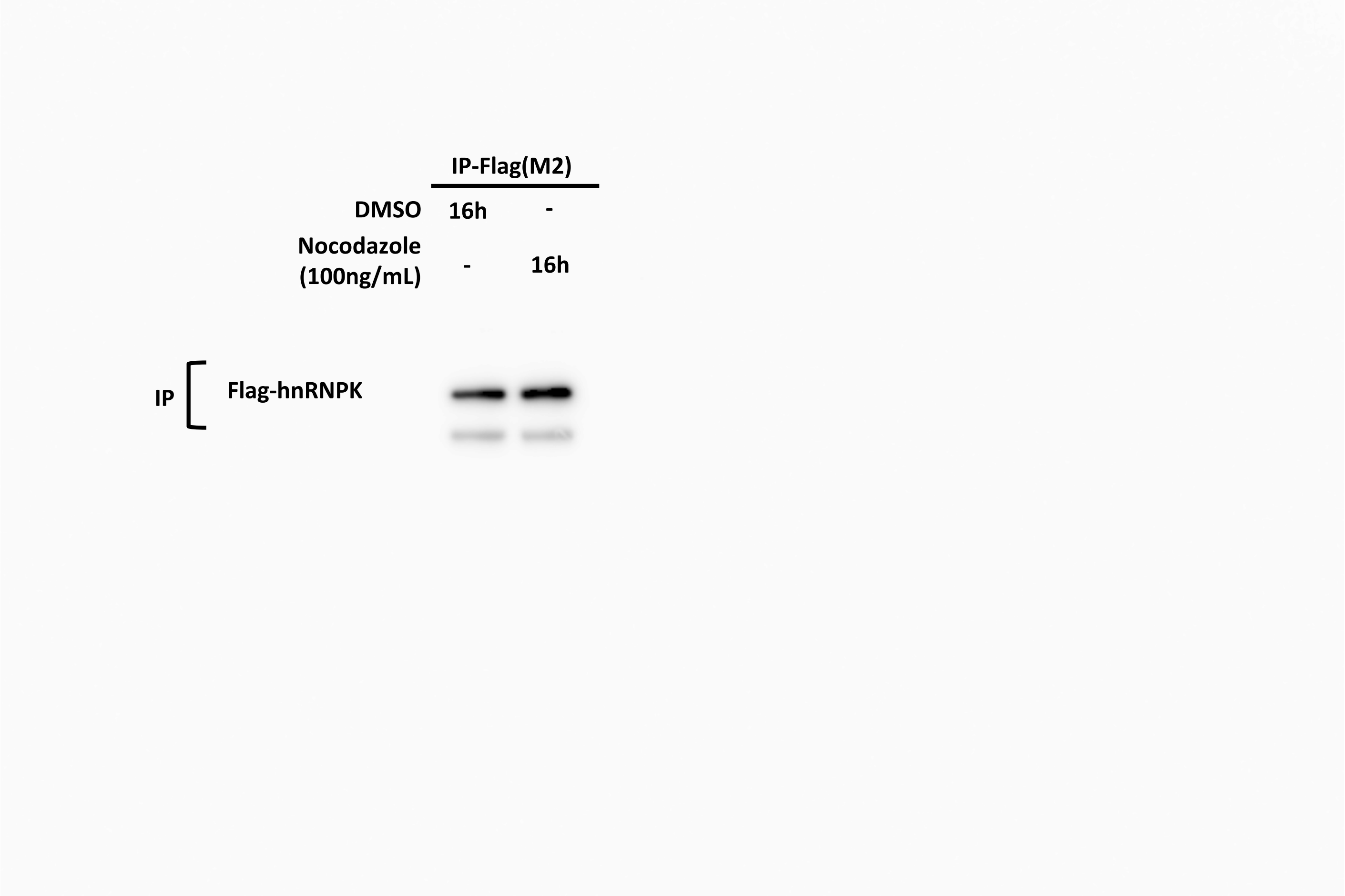

Supplementary figure 5. Continued.

For Figure 1b

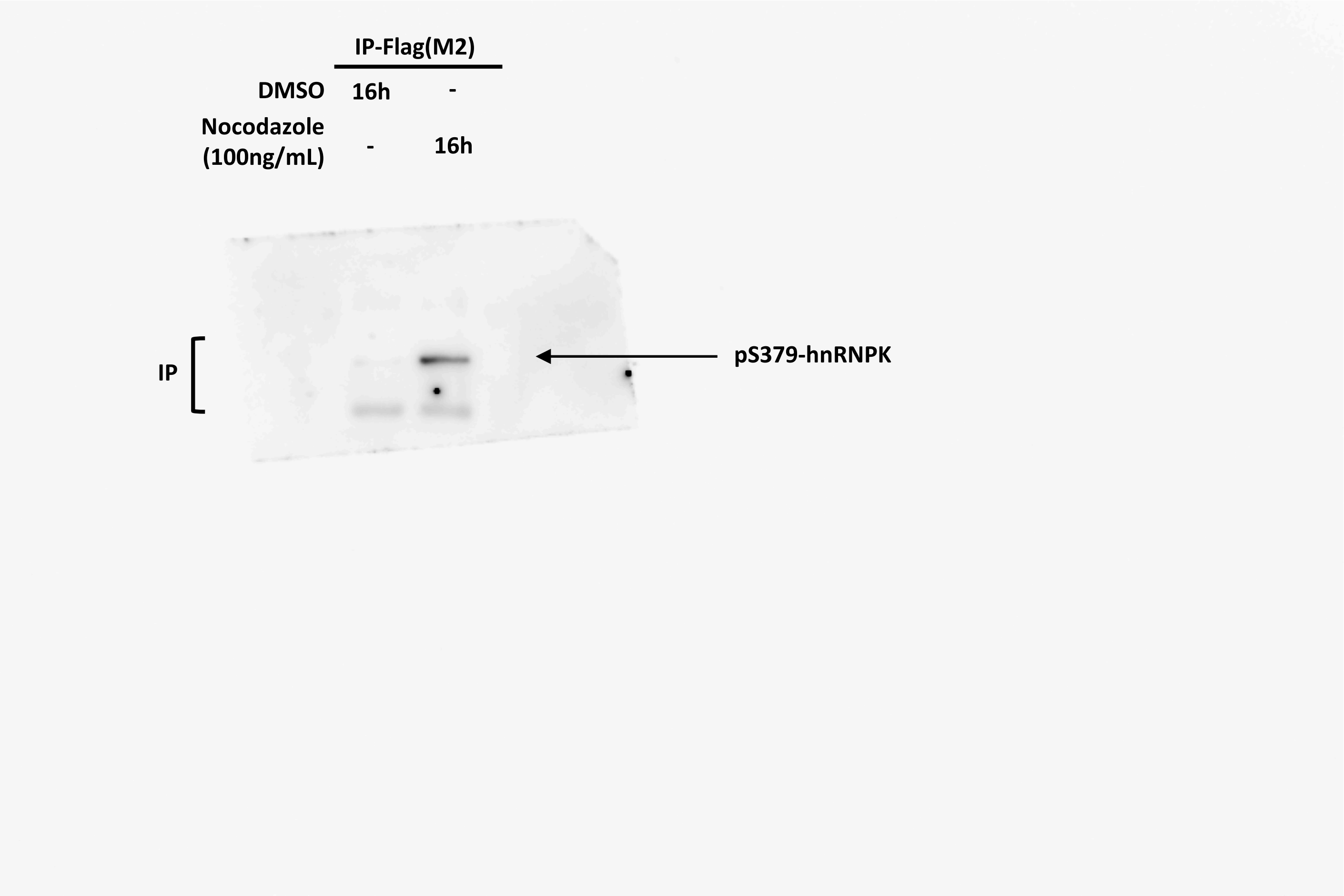

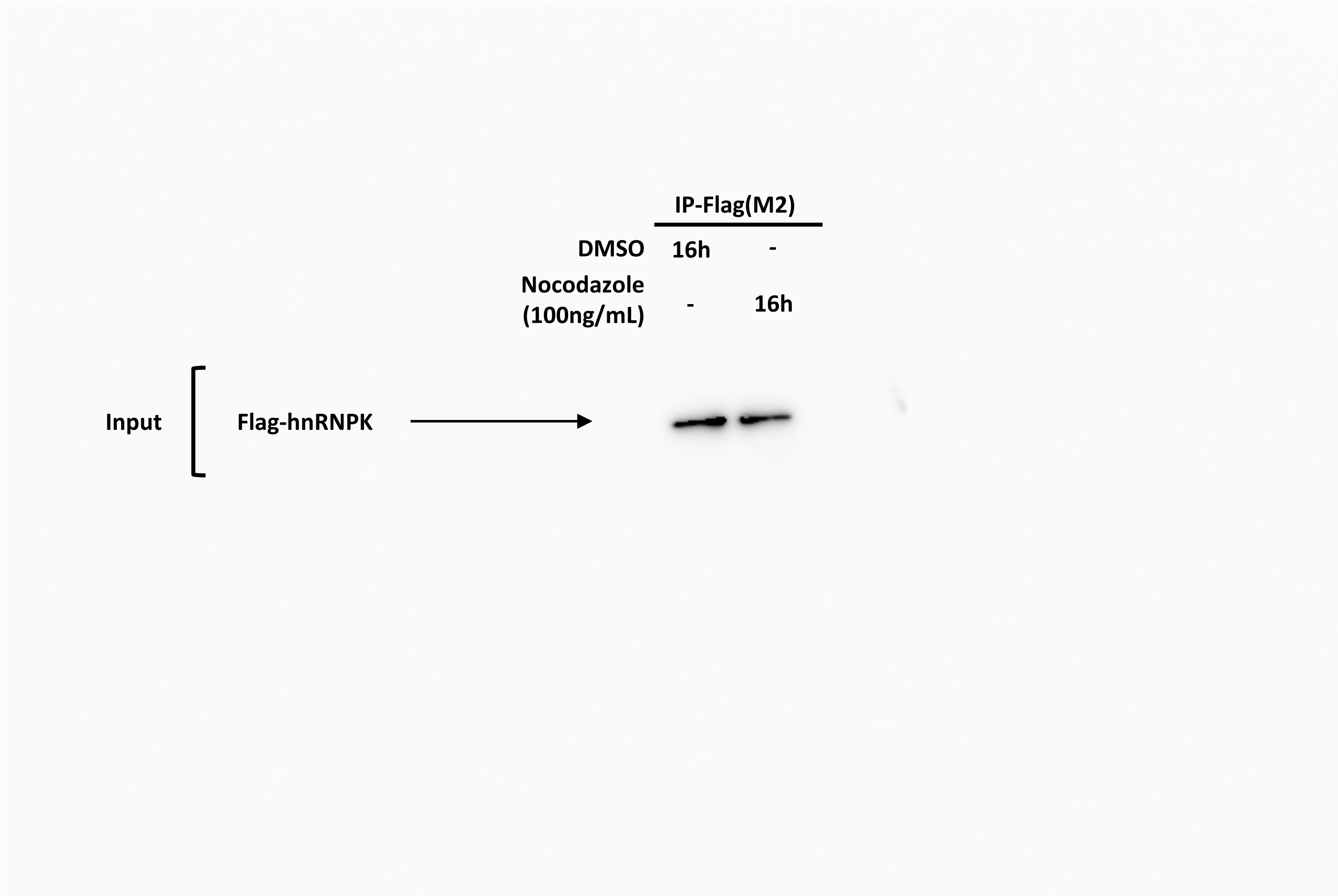

Supplementary figure 5. Continued.

For Figure 1b

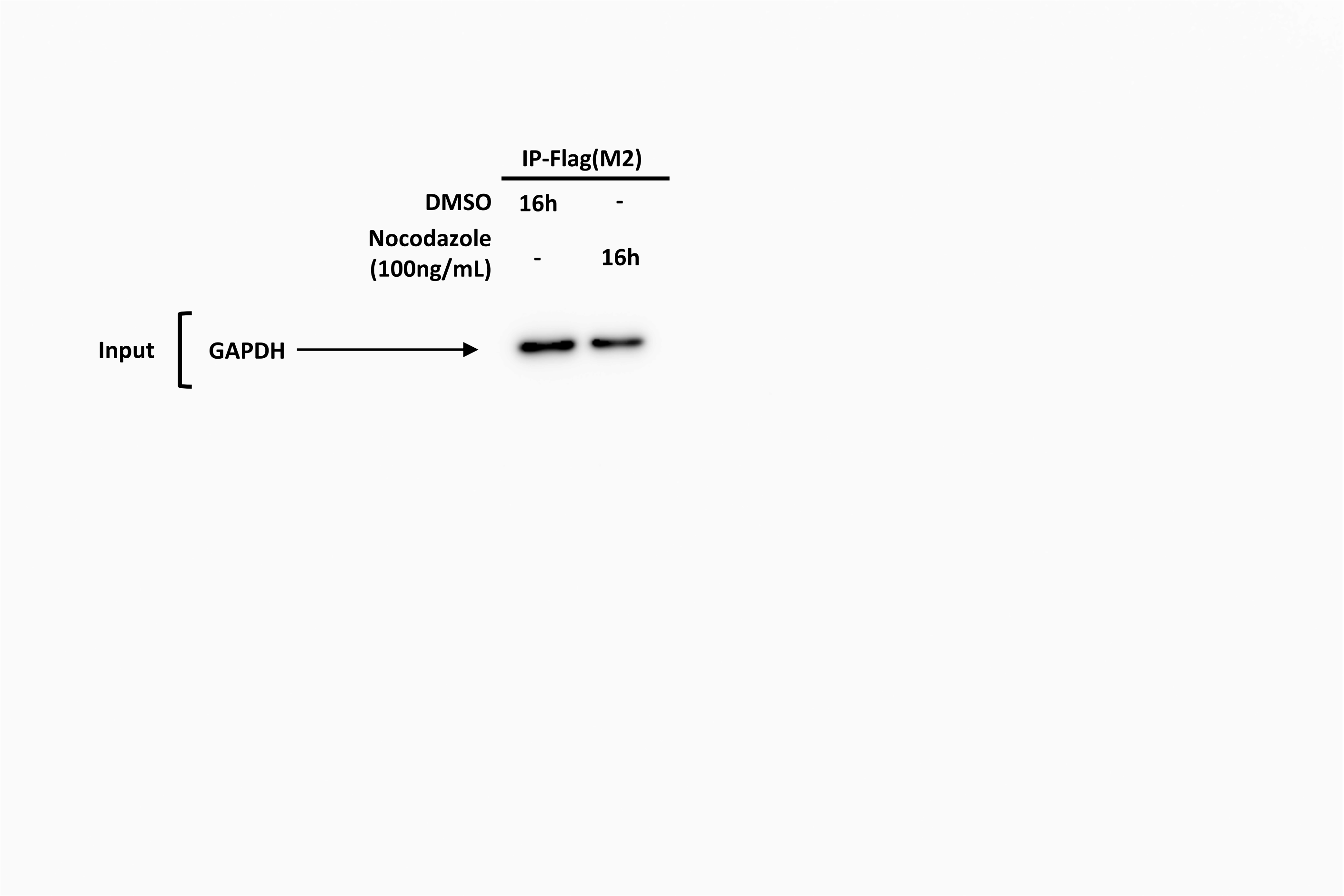

Supplementary figure 5. Continued.

For Figure 2a

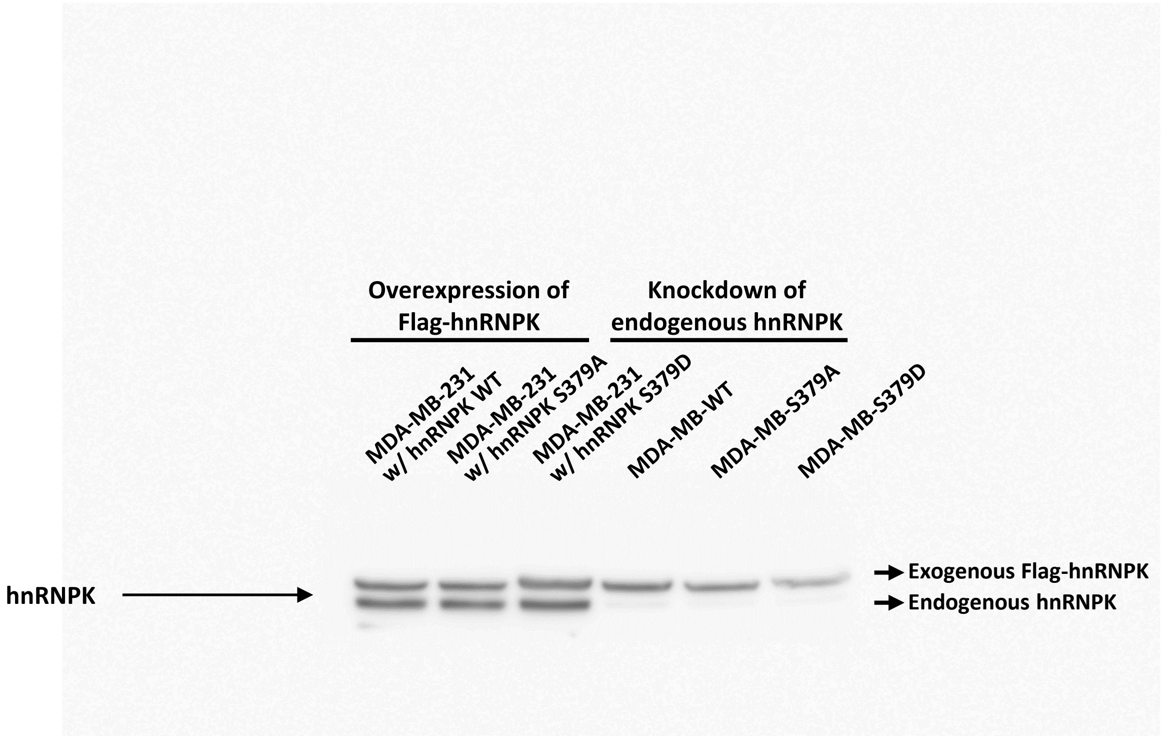

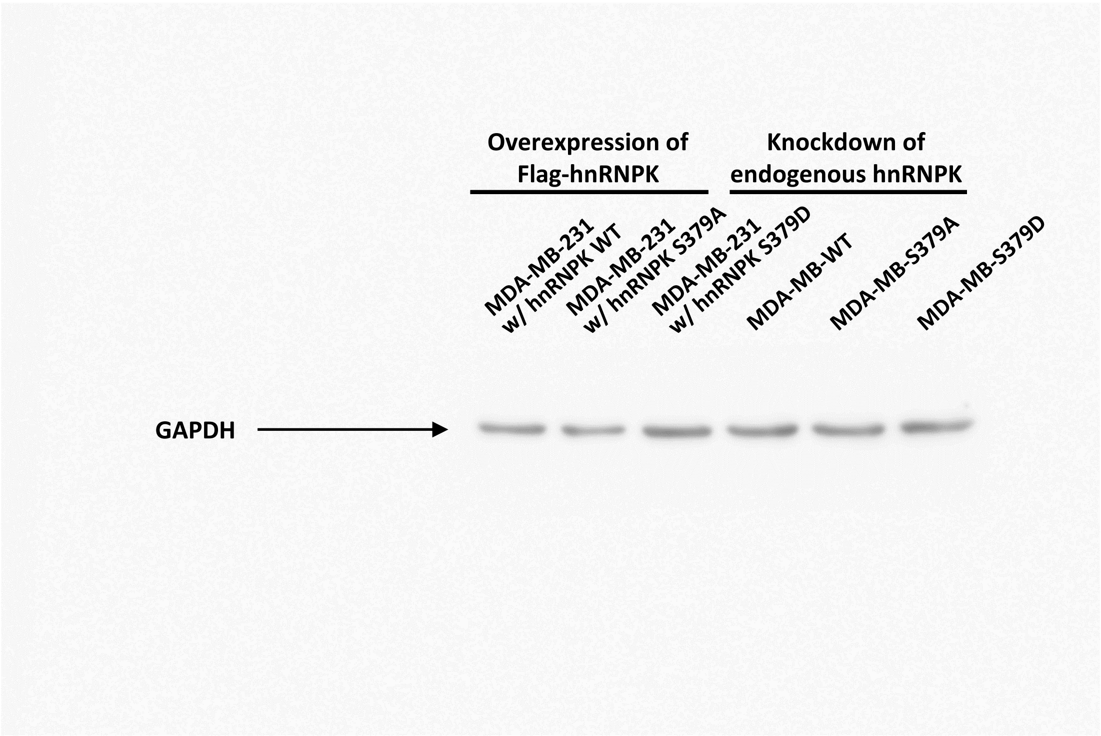

Supplementary figure 6. Raw images of the cropped blots presented in the main figure 2.

For Figure 4a

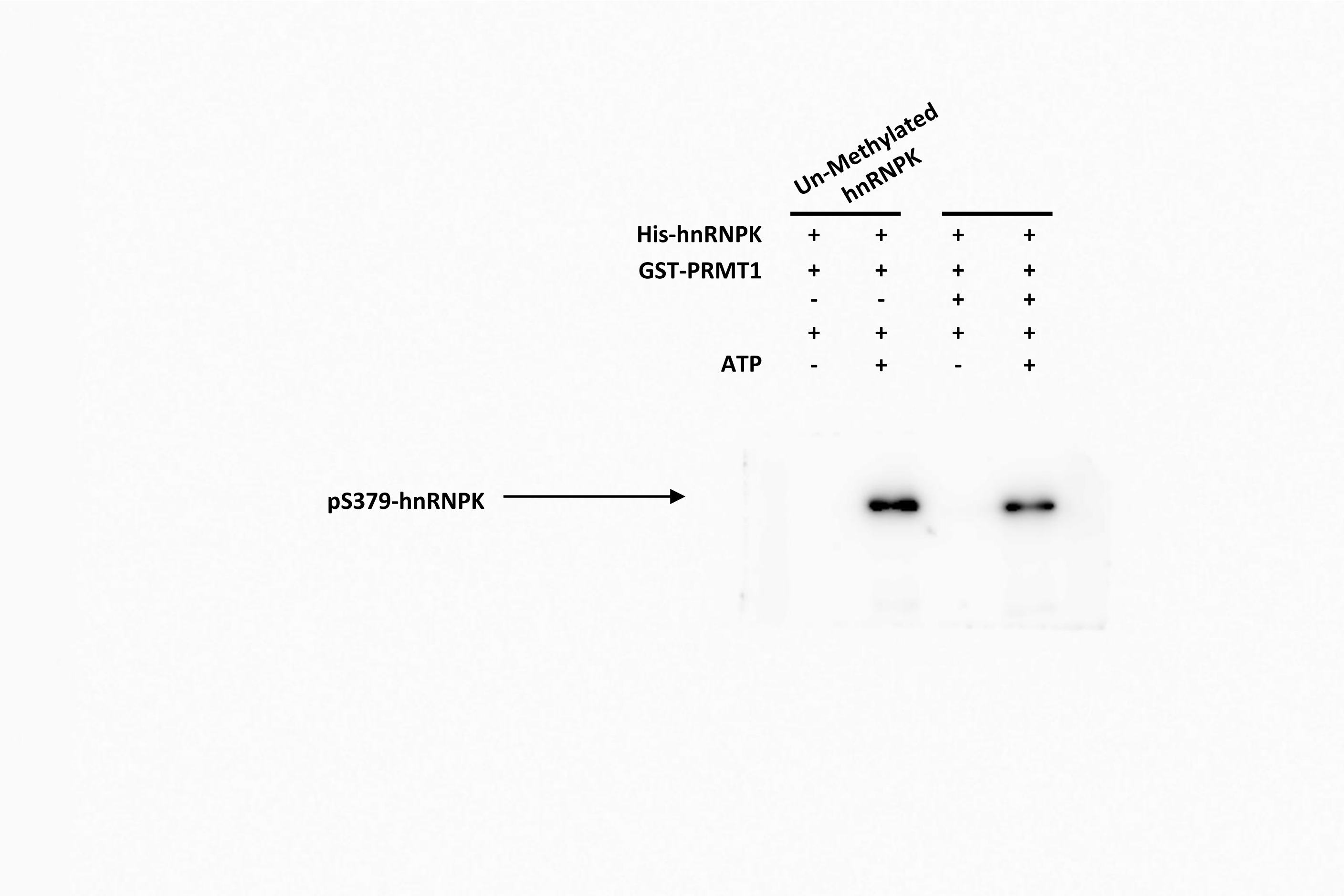

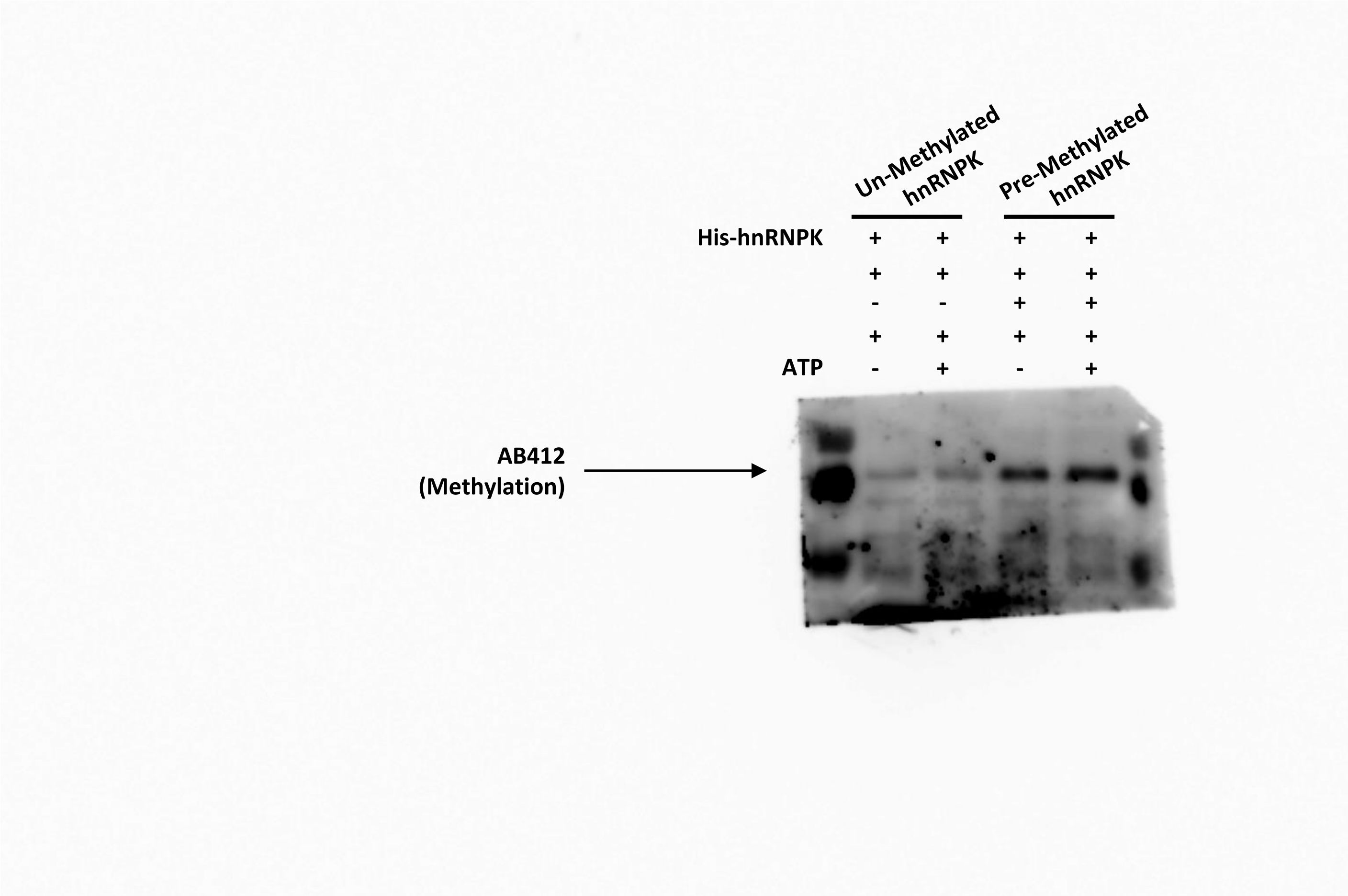

Supplementary figure 7. Raw images of the cropped blots presented in the main figure 4.

For Figure 4a

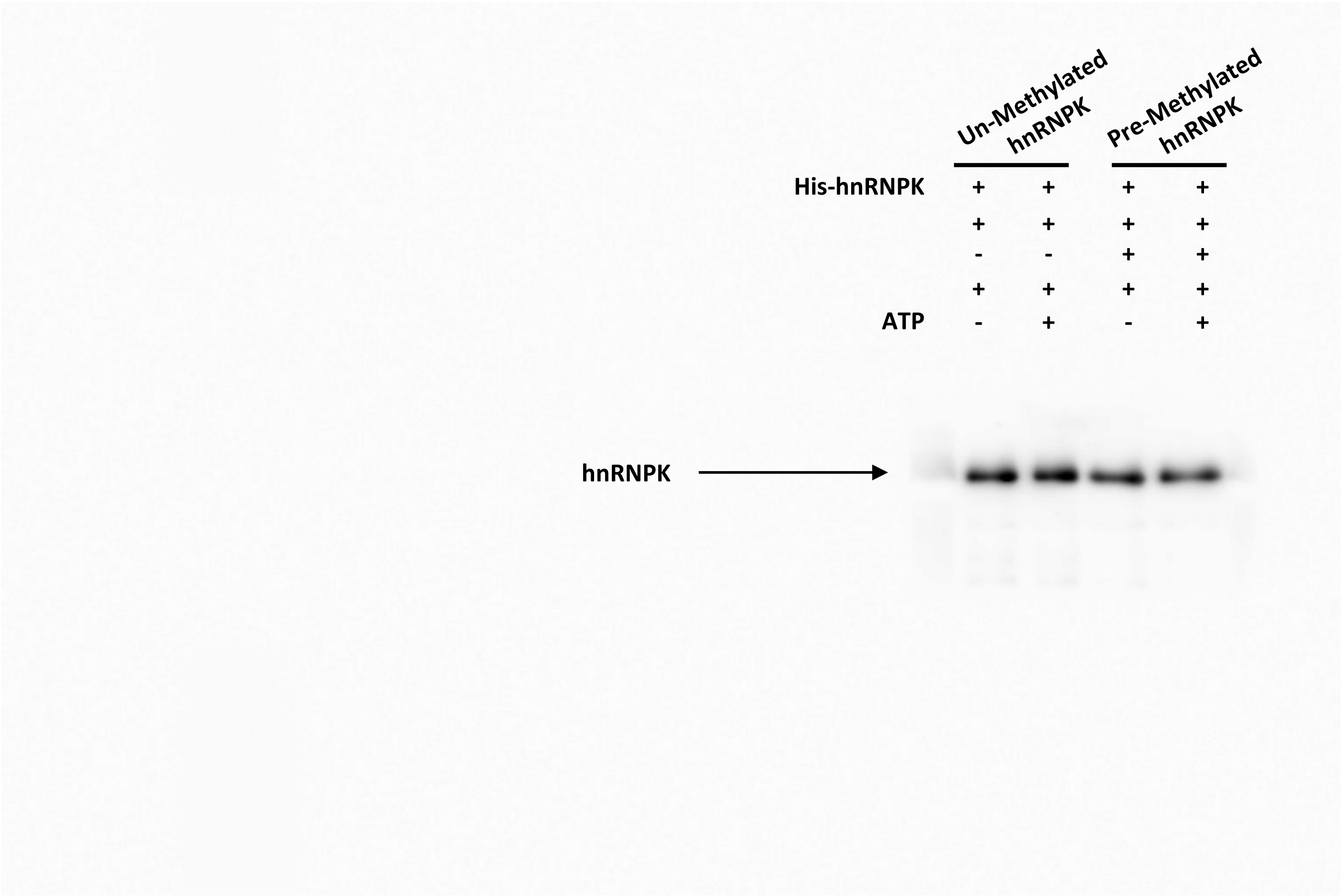

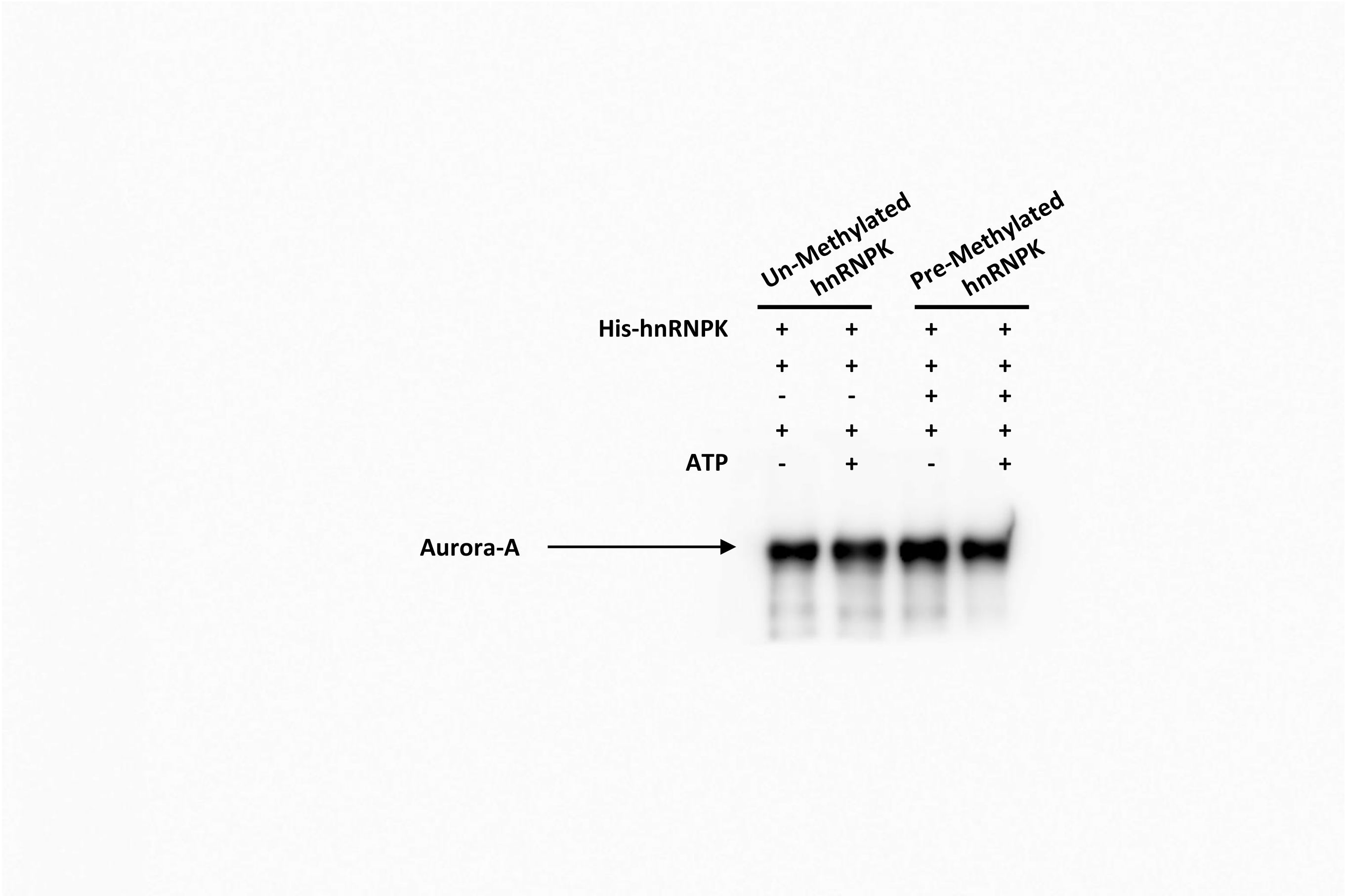

Supplementary figure 7. Continued.

For Figure 4c

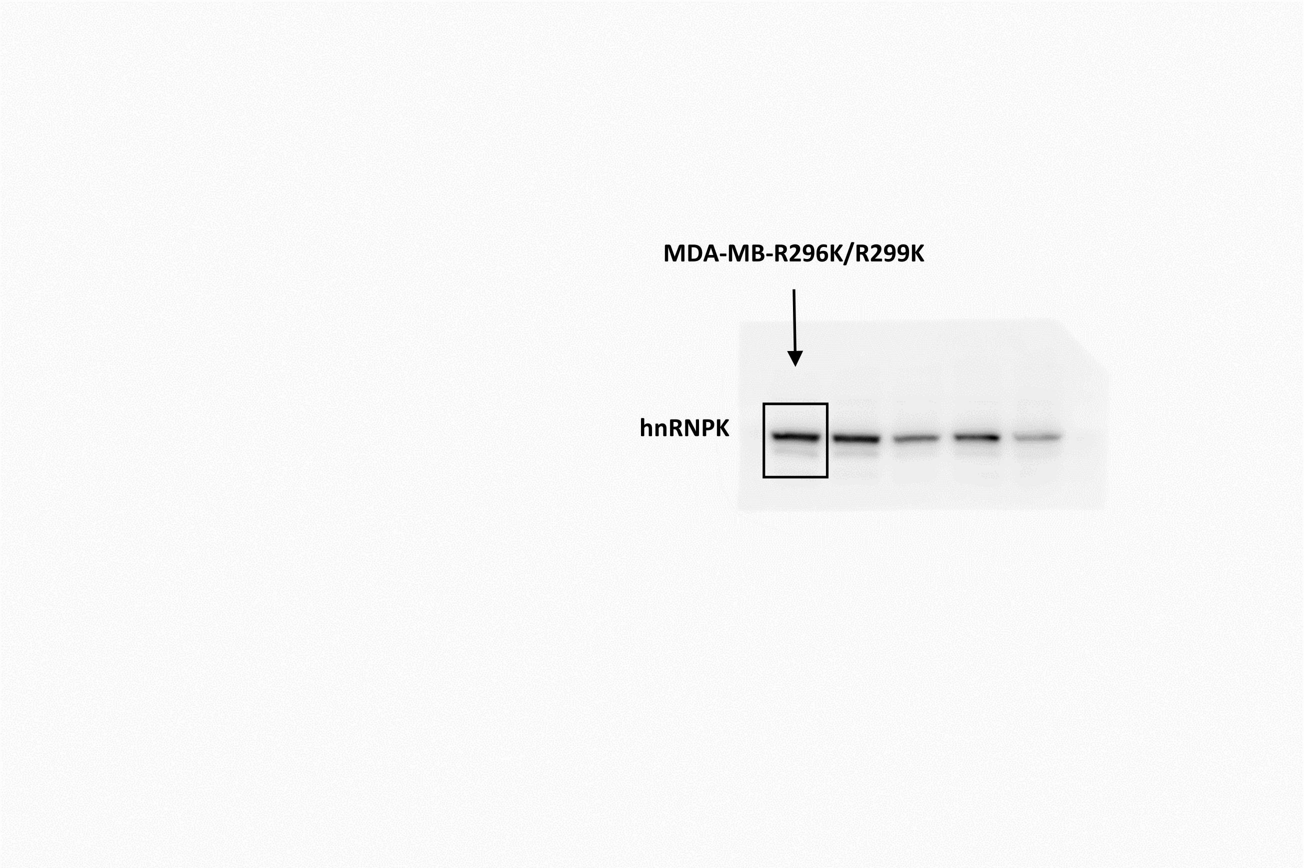

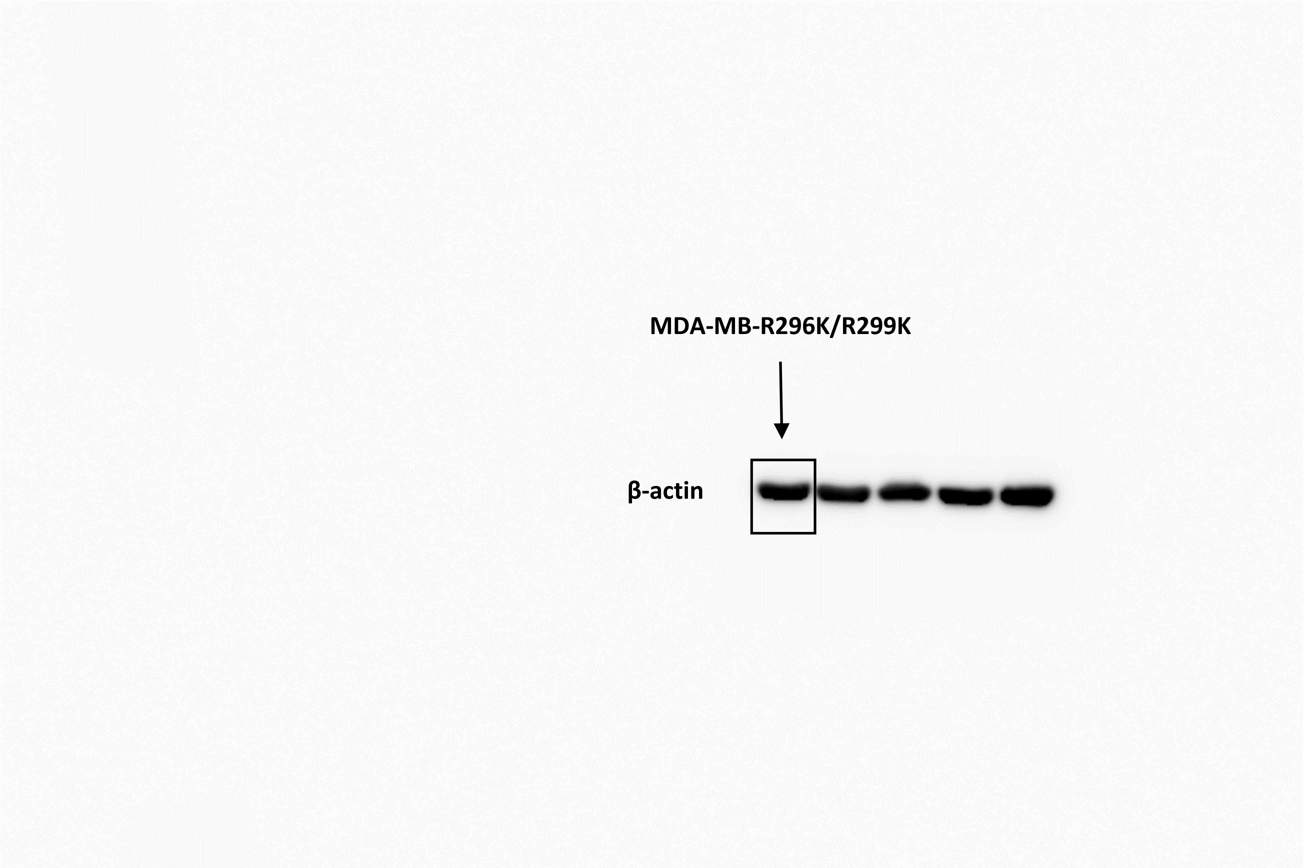

Supplementary figure 7. Continued.

For Figure 4d

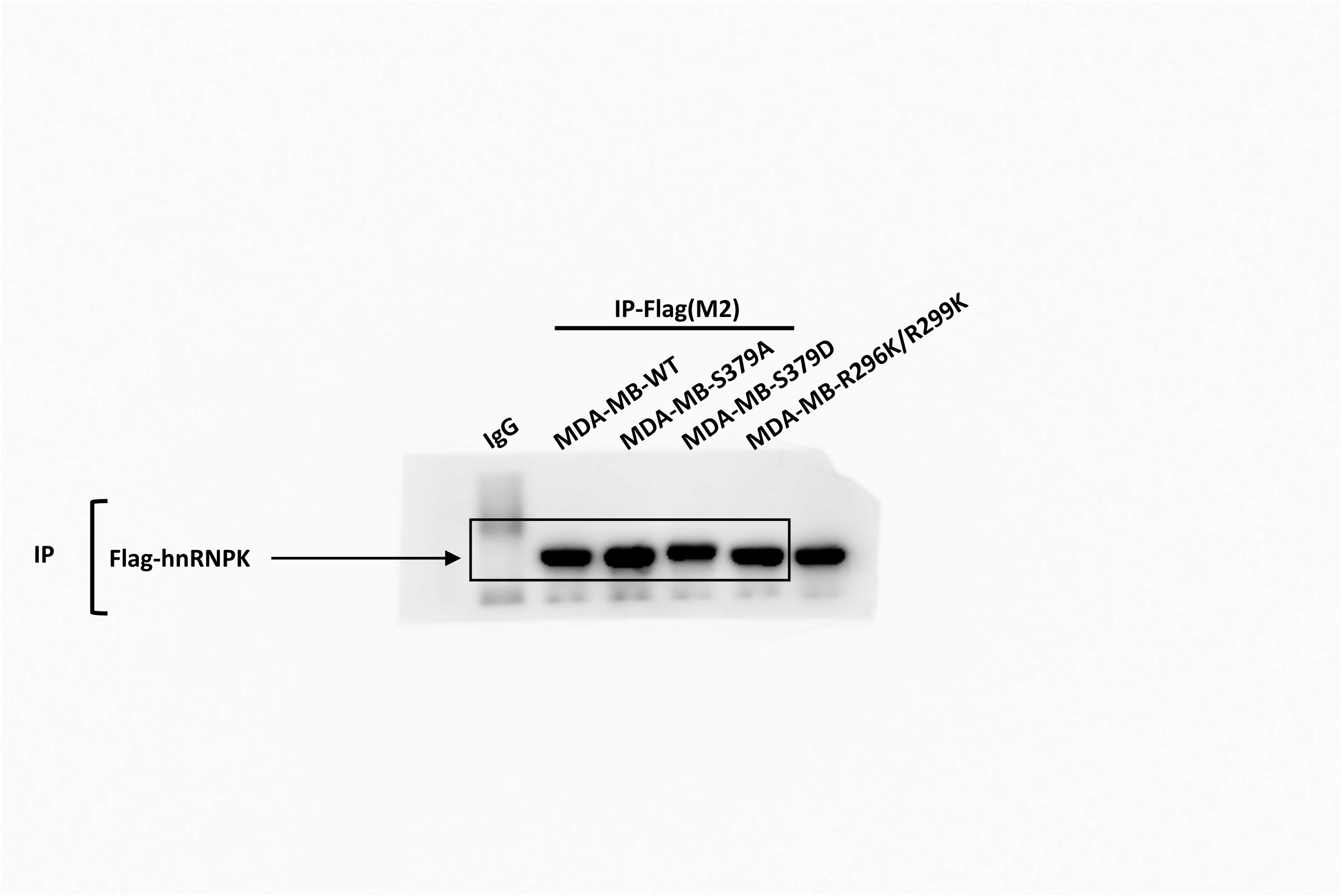

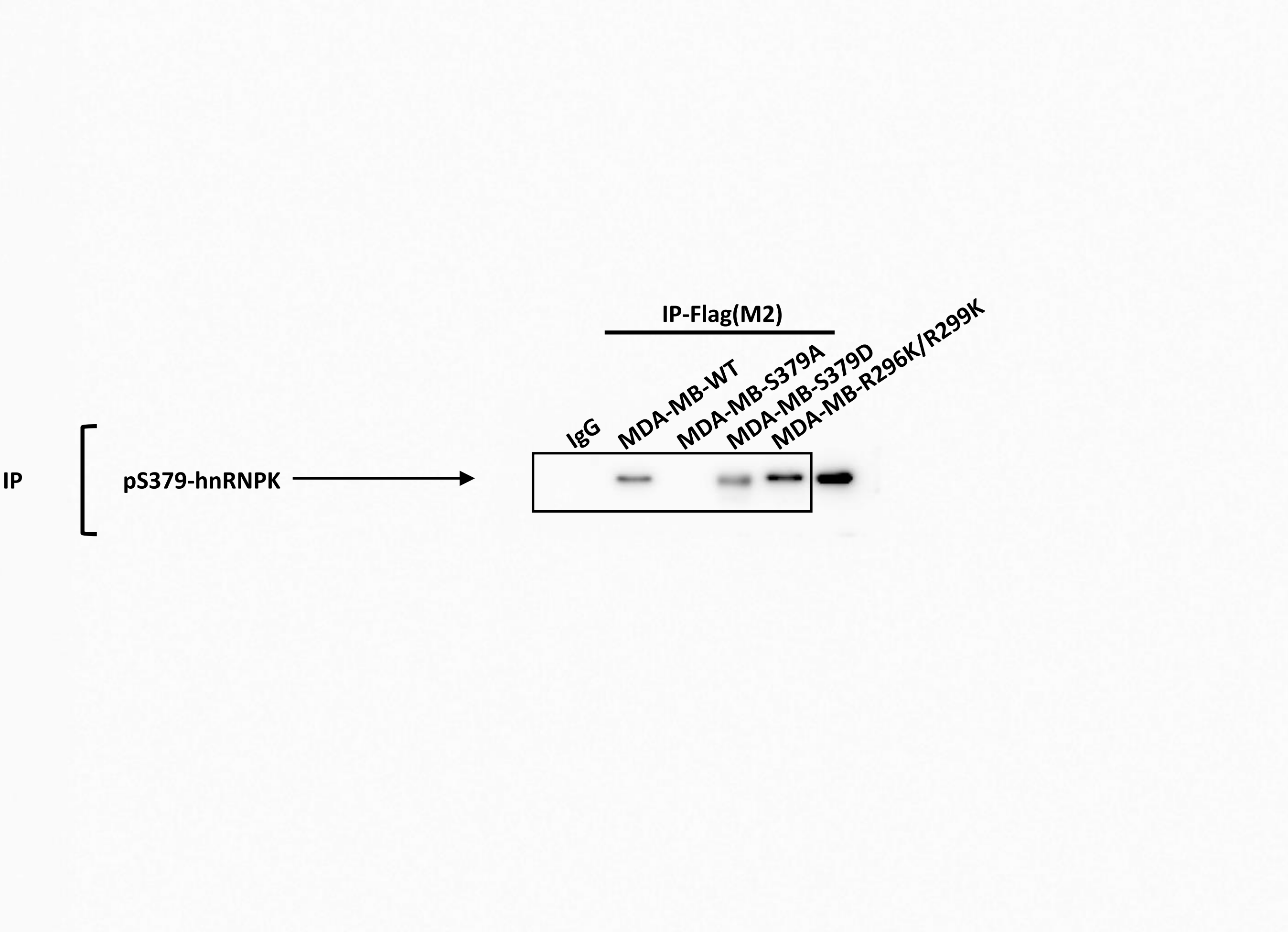

Supplementary figure 7. Continued.

For Figure 4d

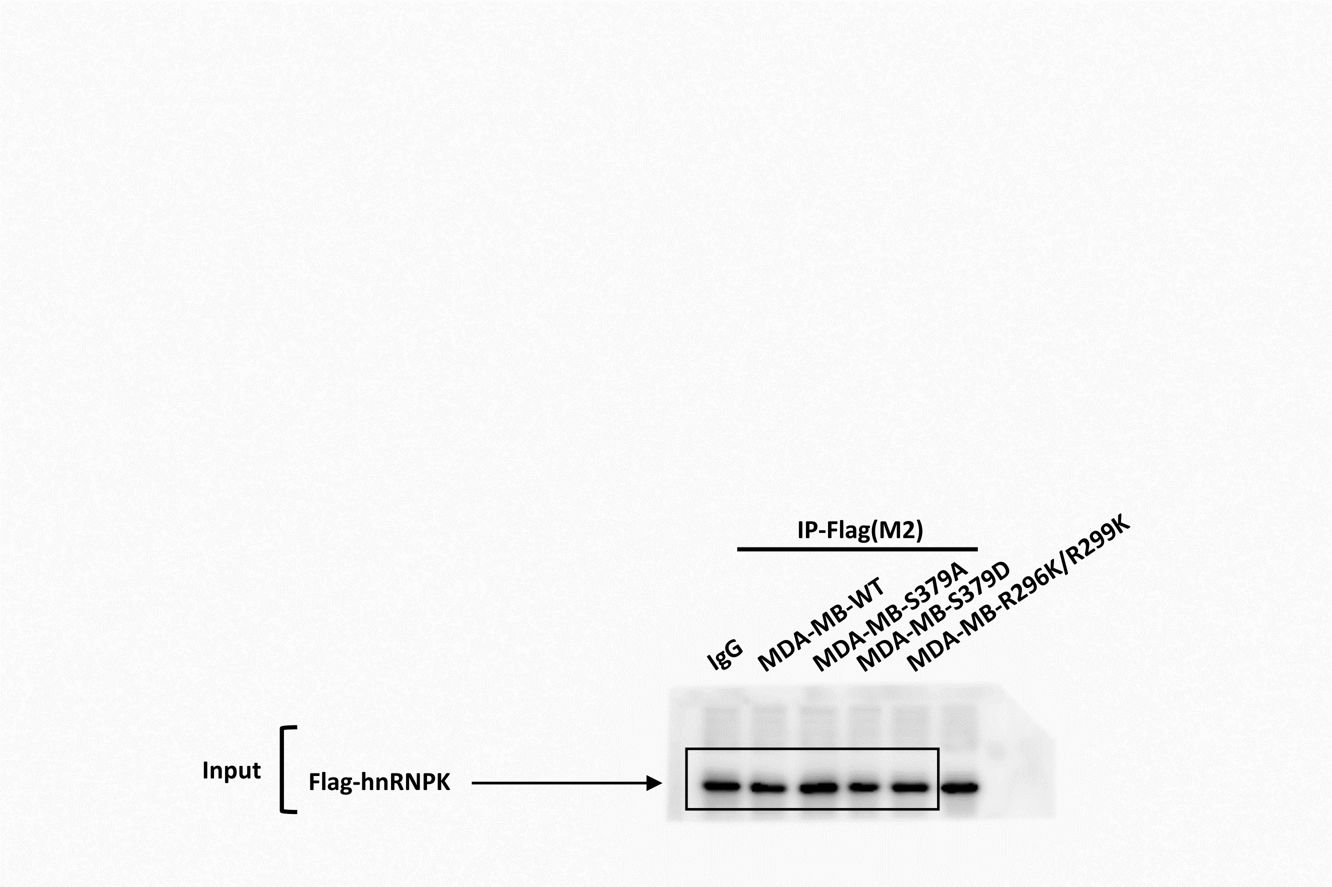

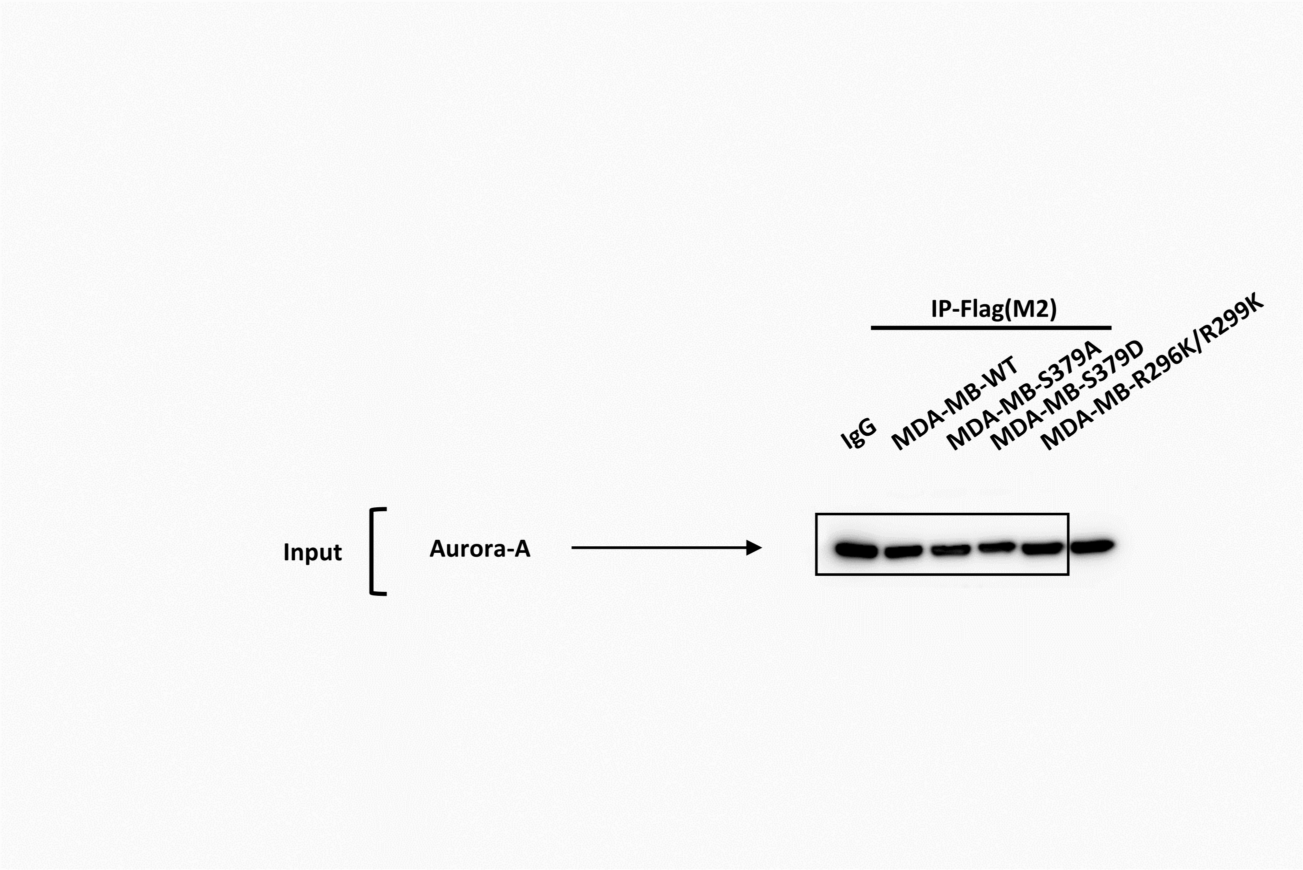

Supplementary figure 7. Continued.

For Figure 4d

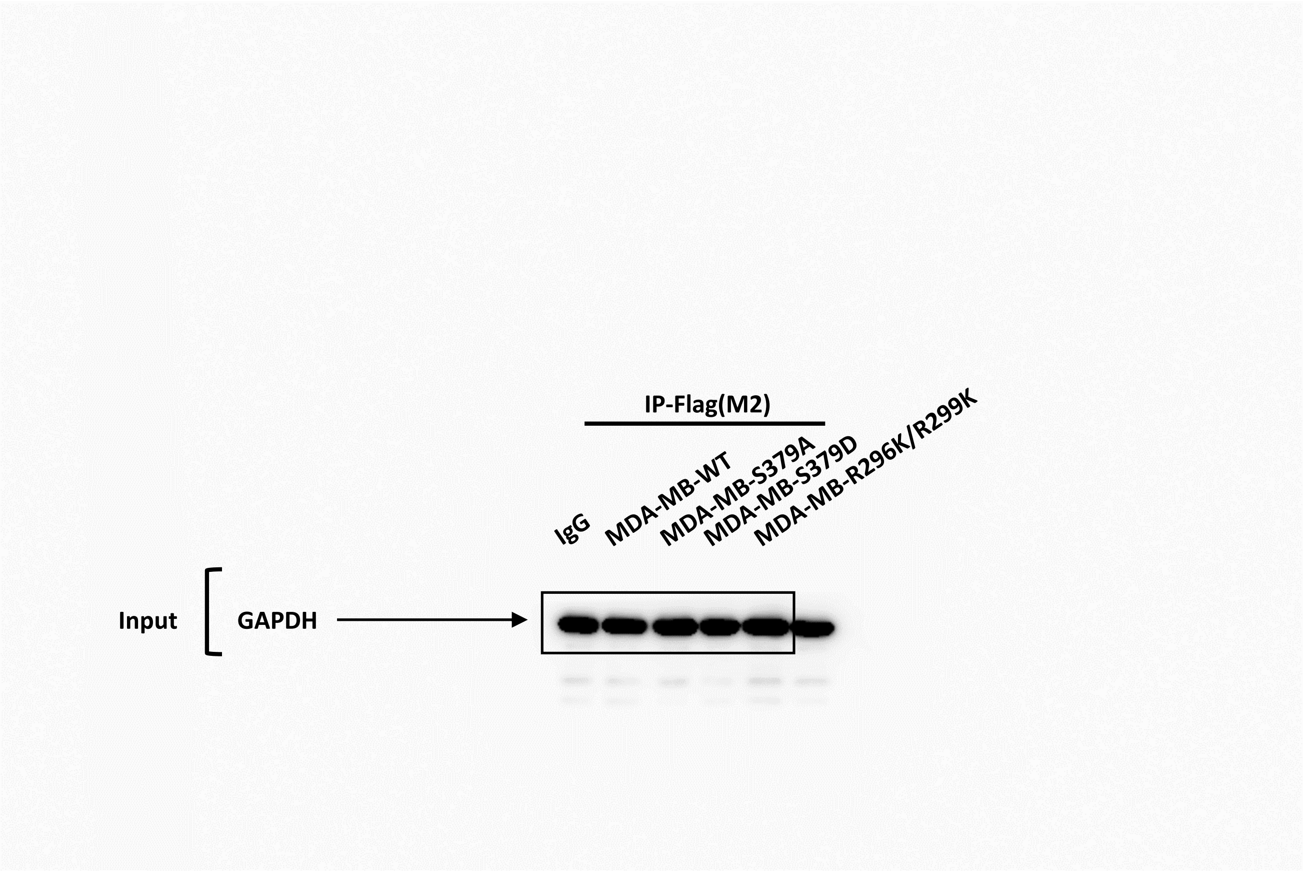

Supplementary figure 7. Continued.

For figure 5b

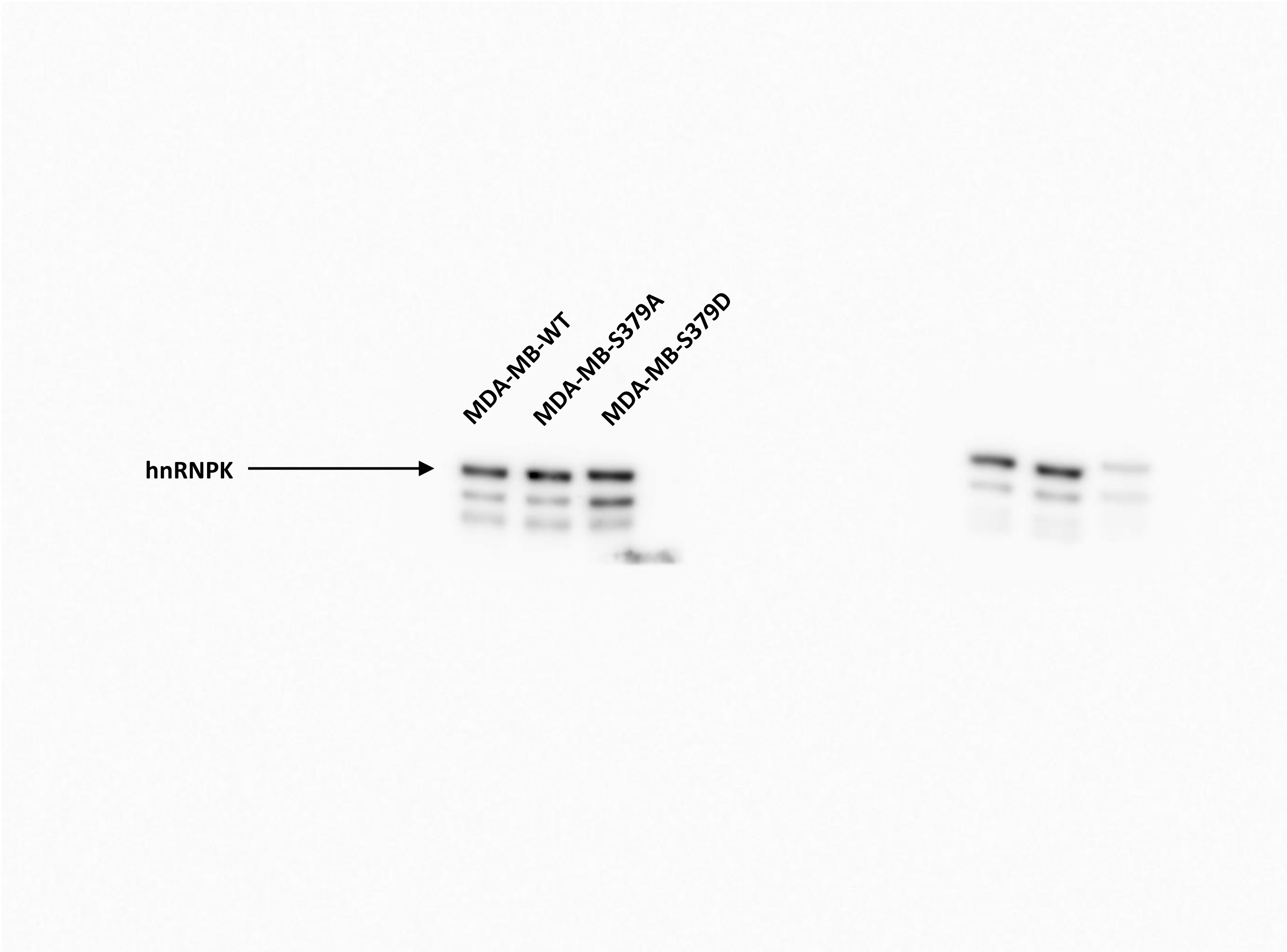

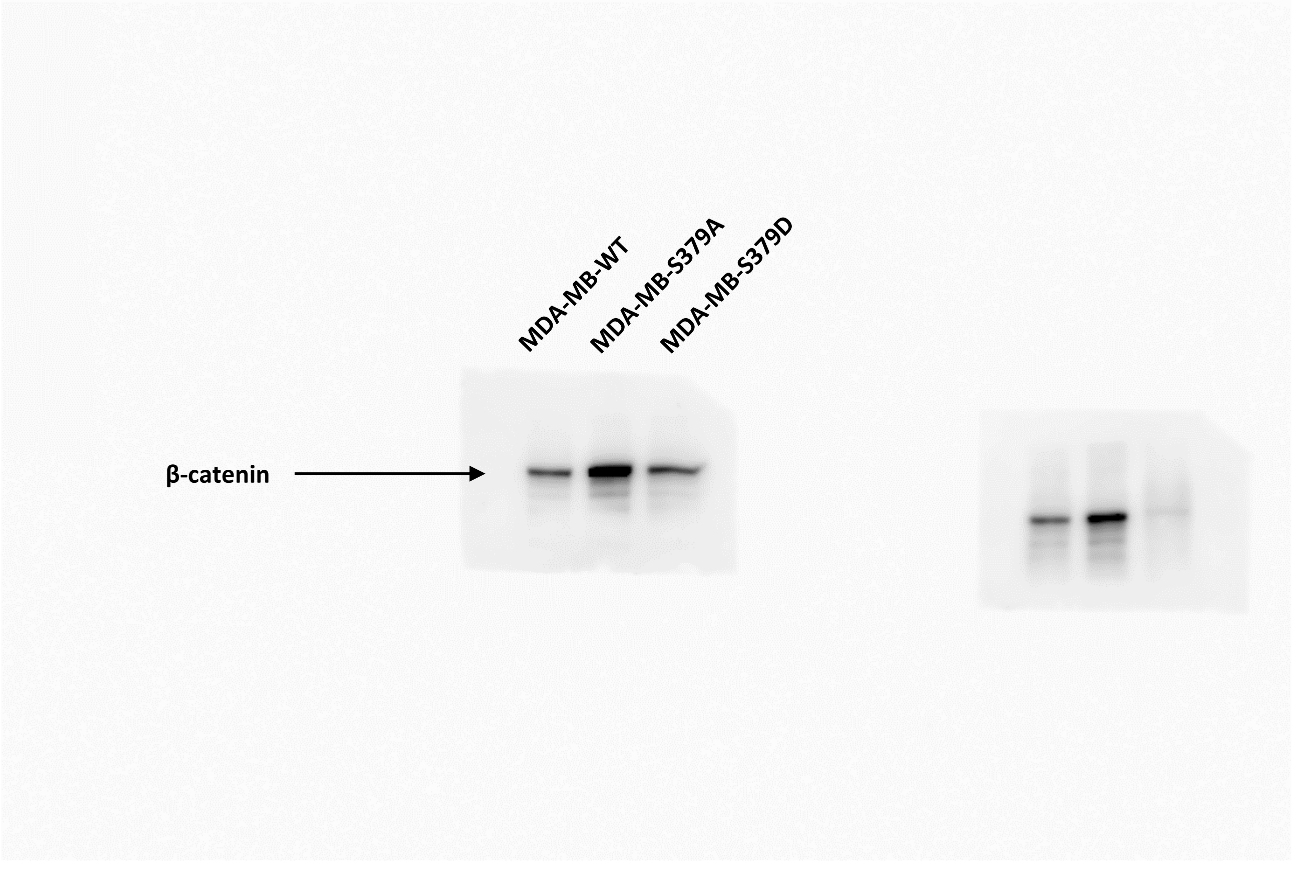

Supplementary figure 8. Raw images of the cropped blots presented in the main figure 5.

For figure 5b

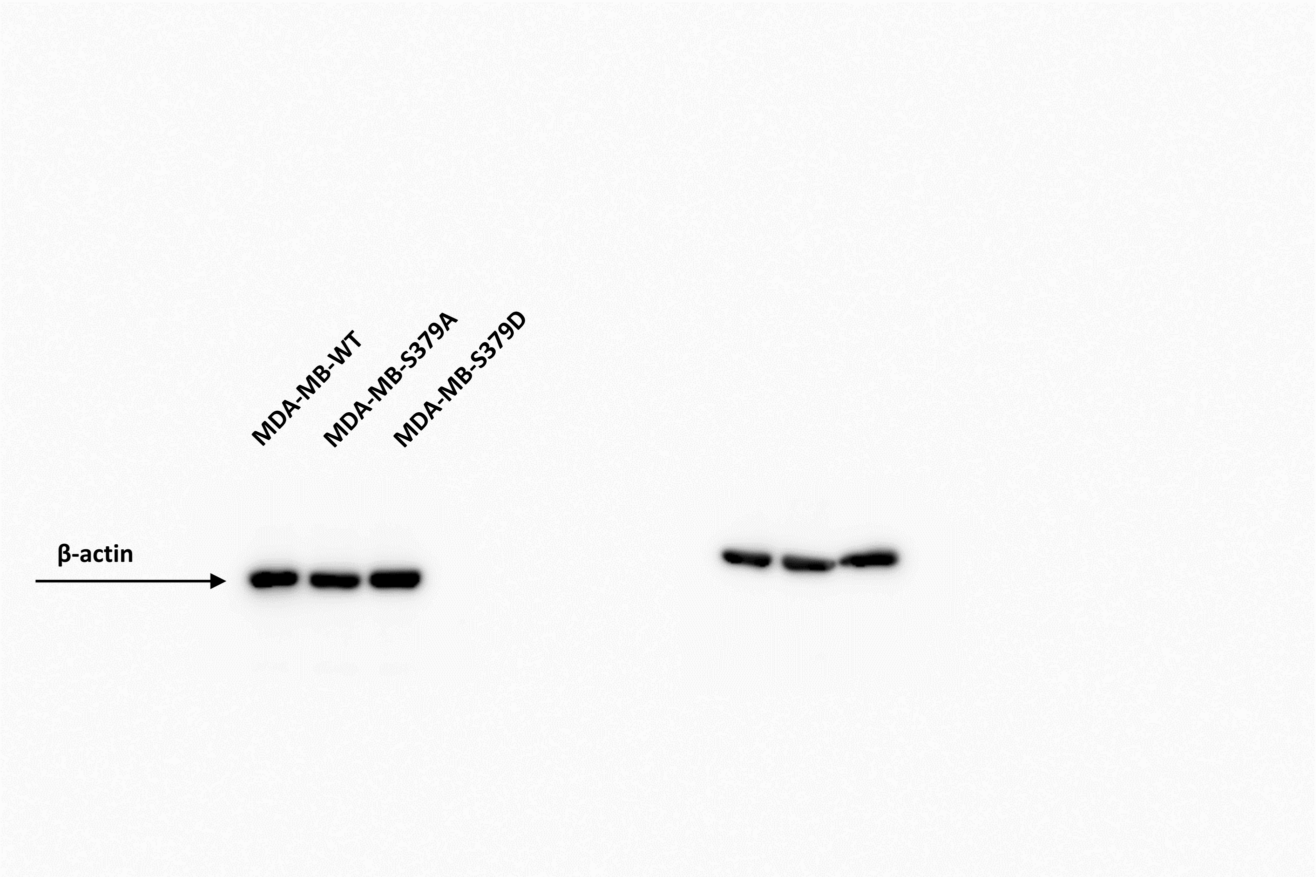

Supplementary figure 8. Continued.

For figure 5b

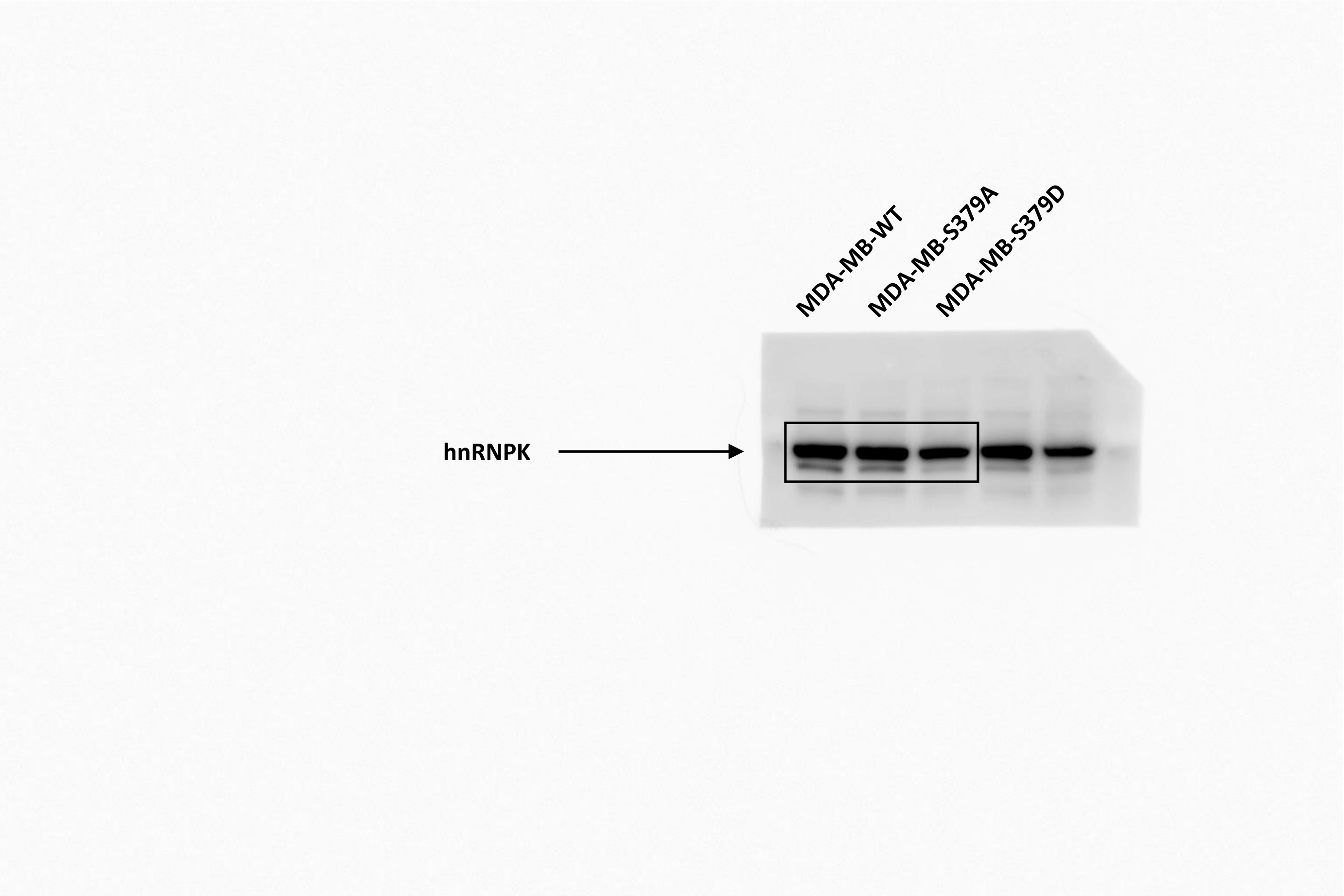

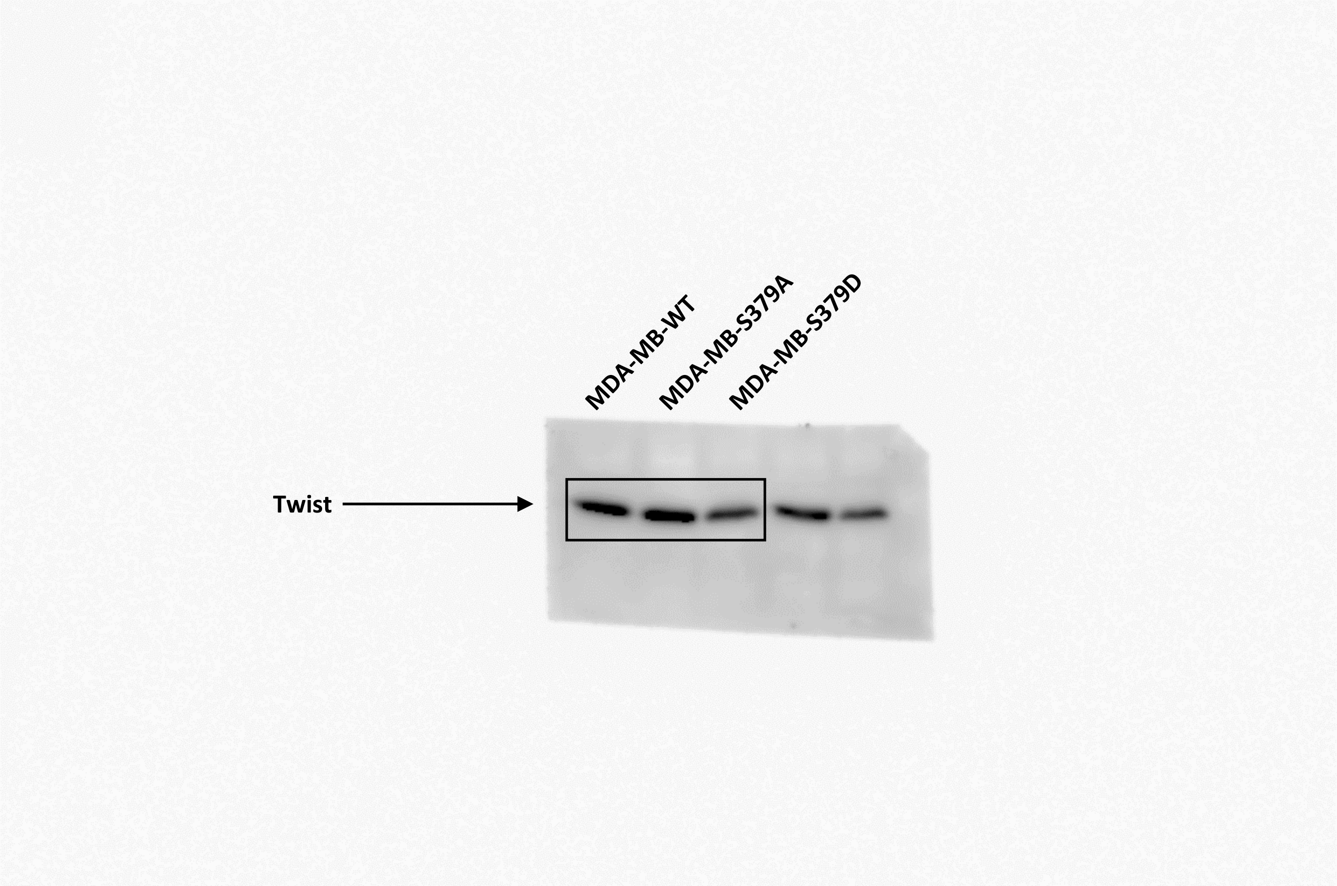

Supplementary figure 8. Continued.

For figure 5b

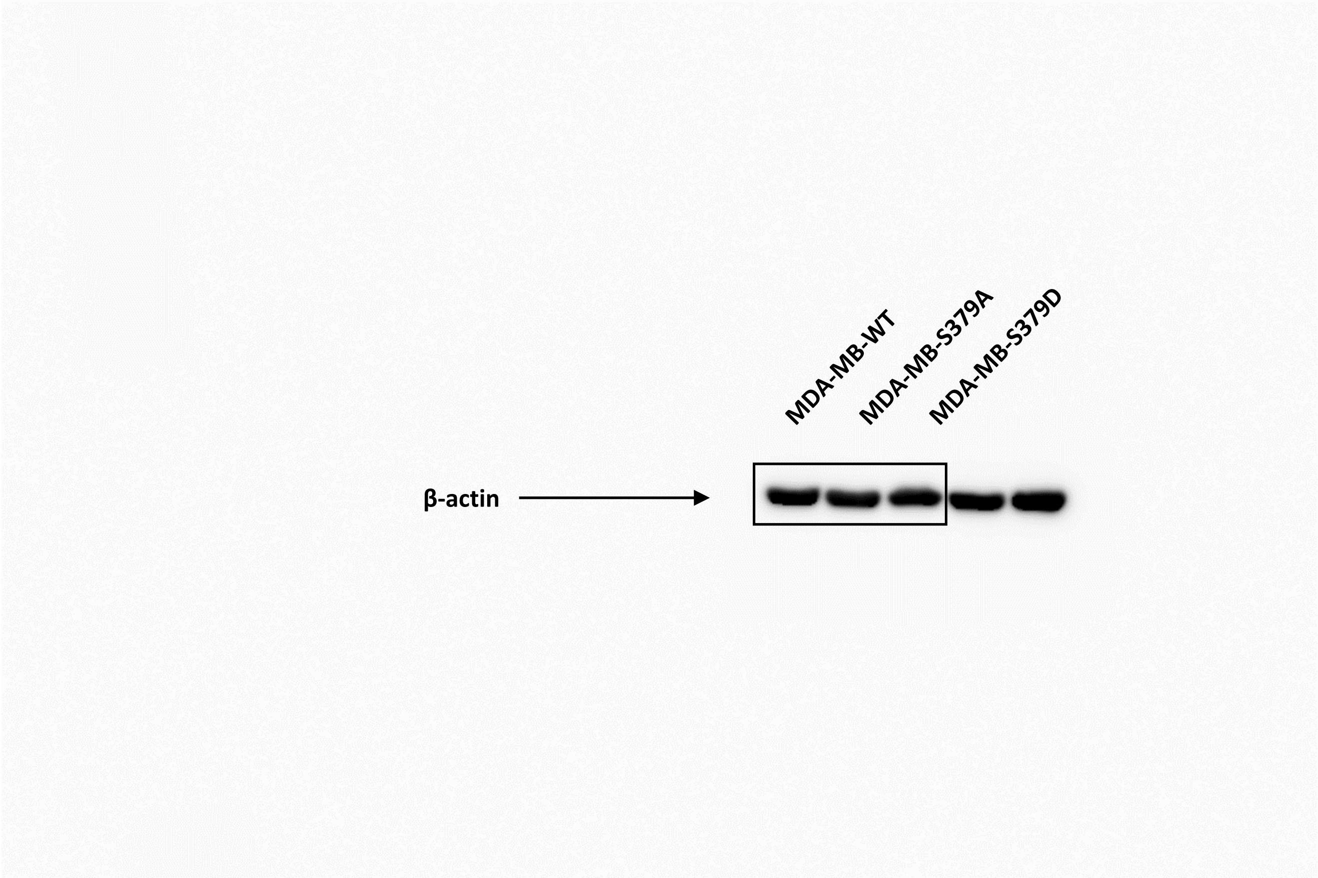

Supplementary figure 8. Continued.

For figure 5f

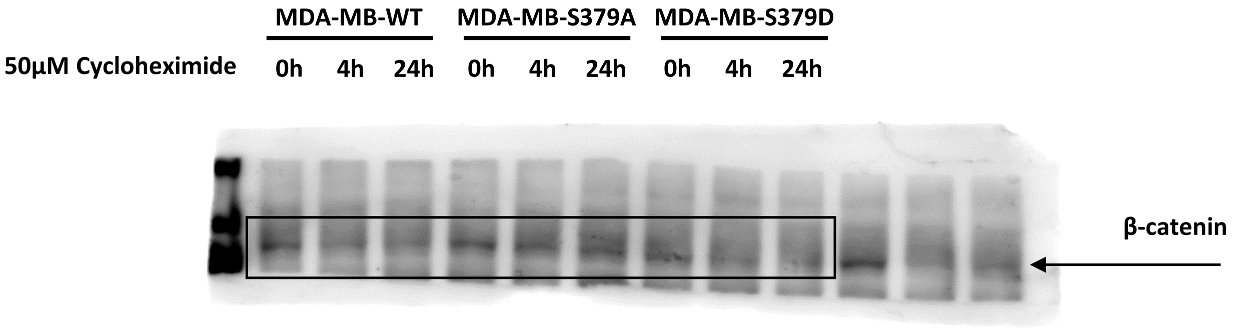

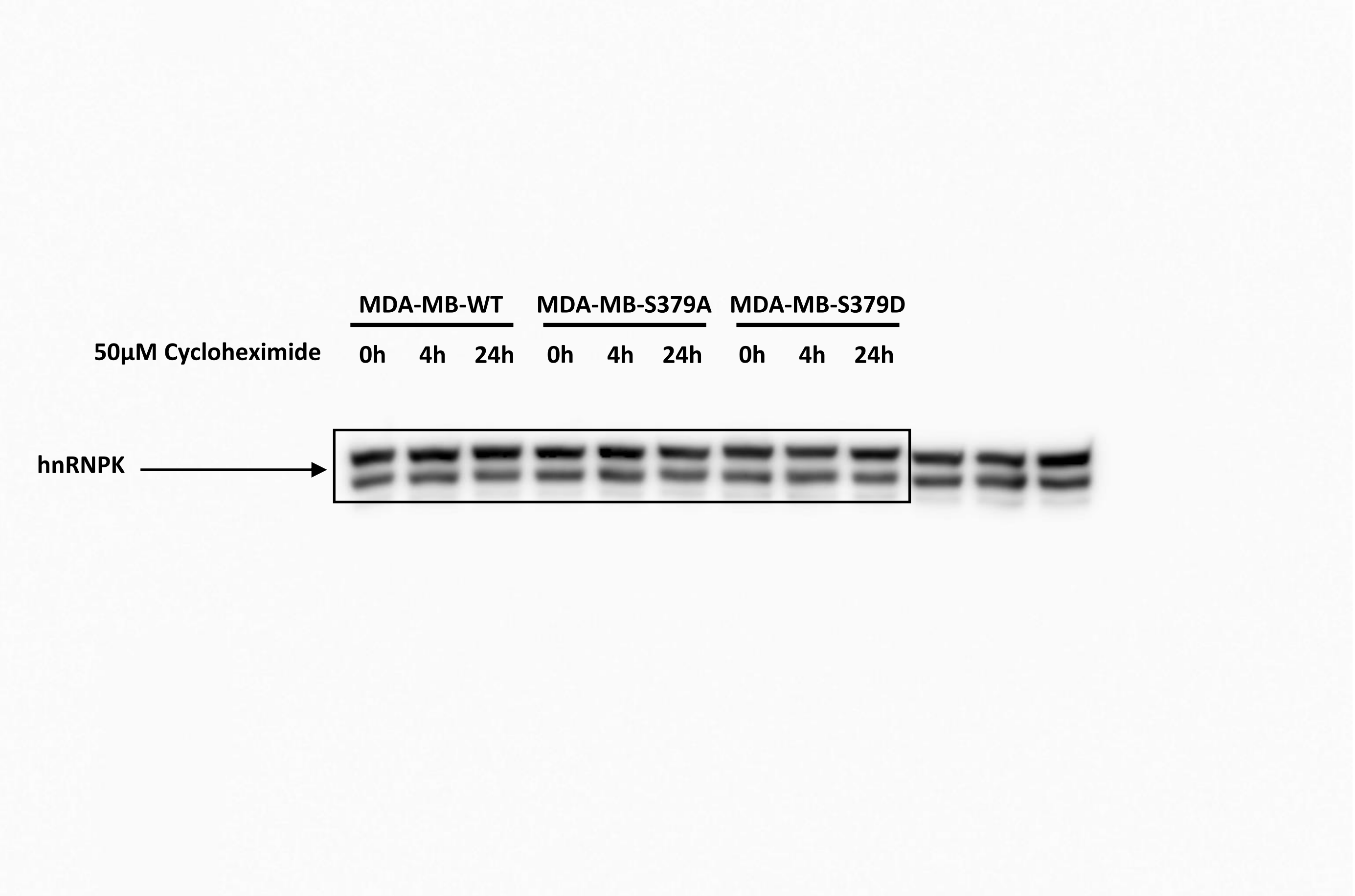

Supplementary figure 8. Continued.

For figure 5f

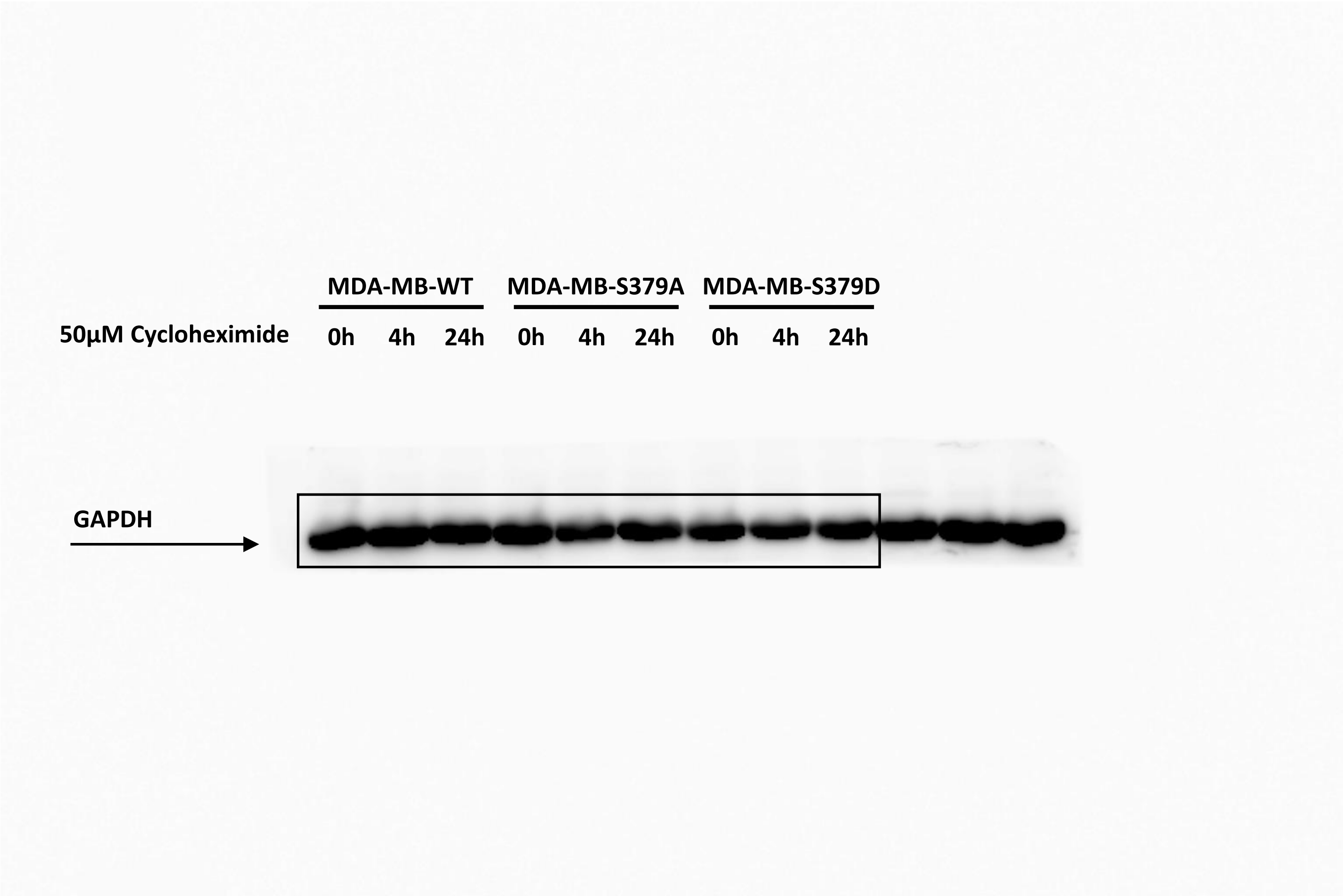

Supplementary figure 8. Continued.

For figure 6c

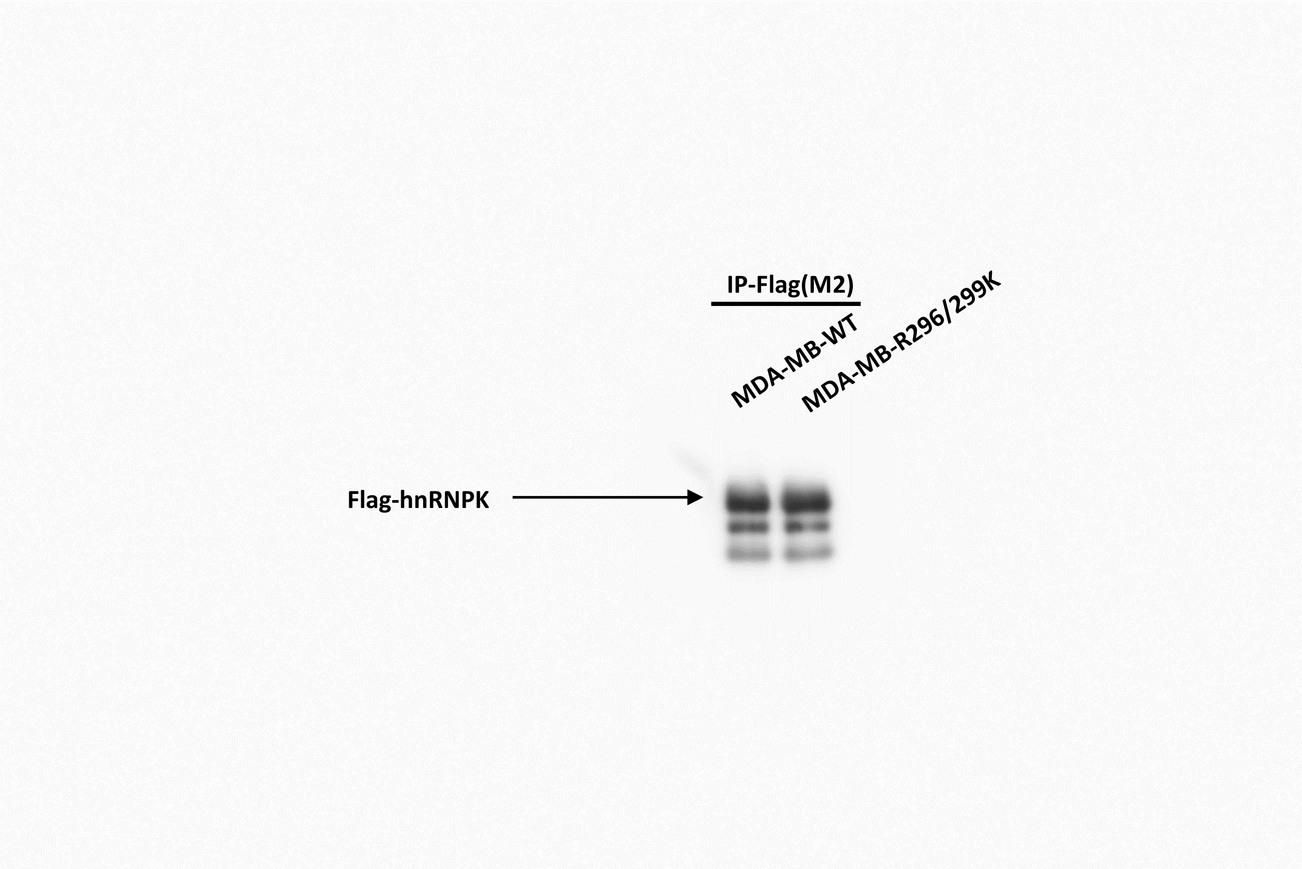

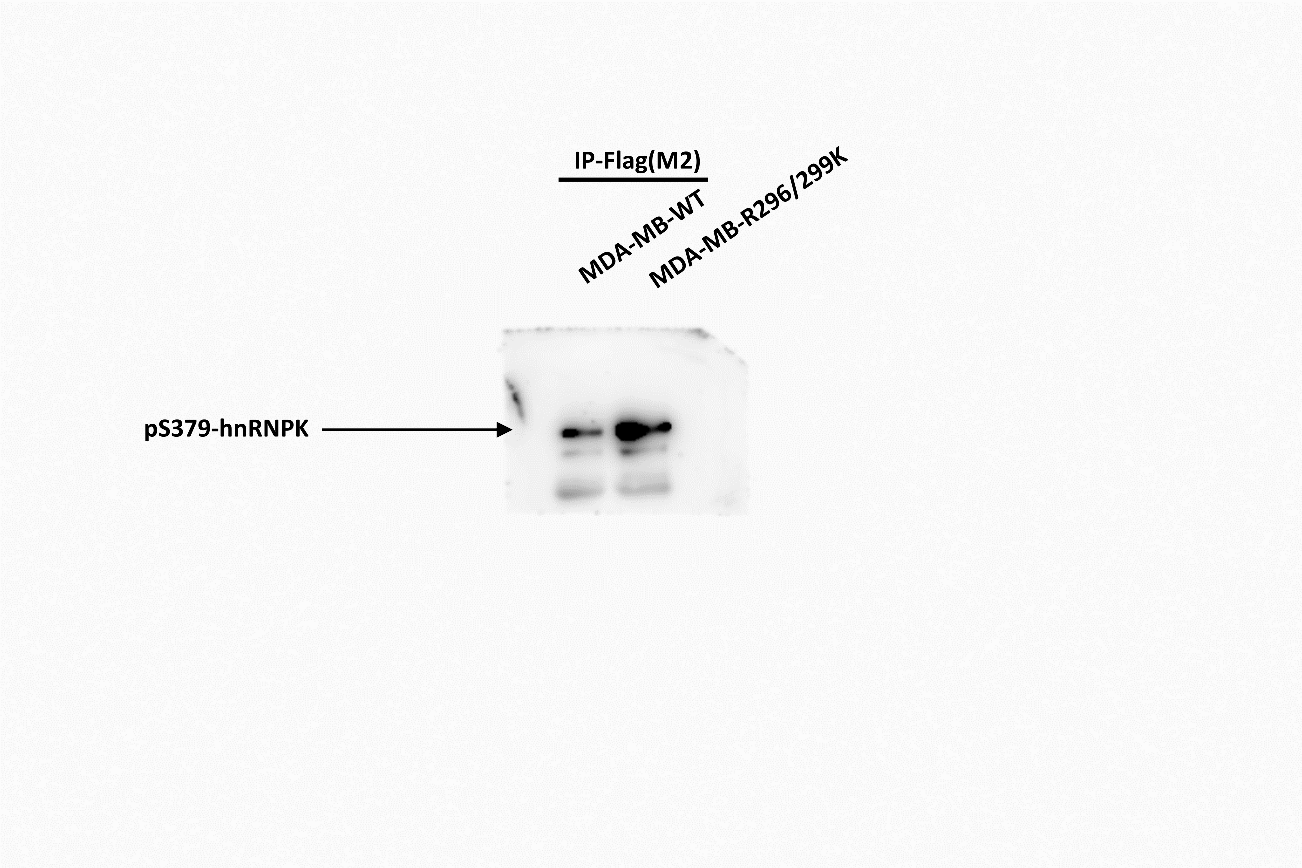

Supplementary figure 9. Raw images of the cropped blots presented in the main figure 6.

For figure 6c

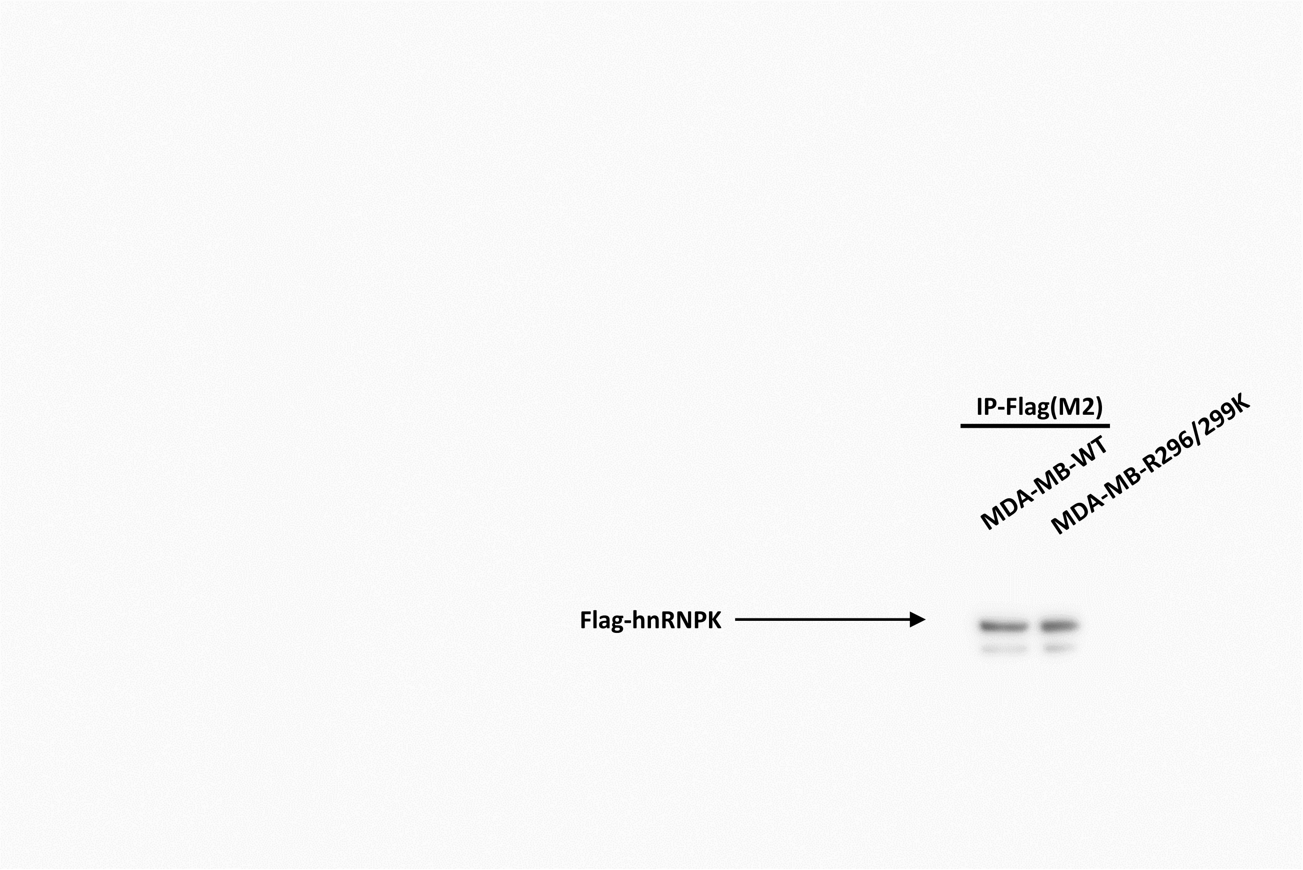

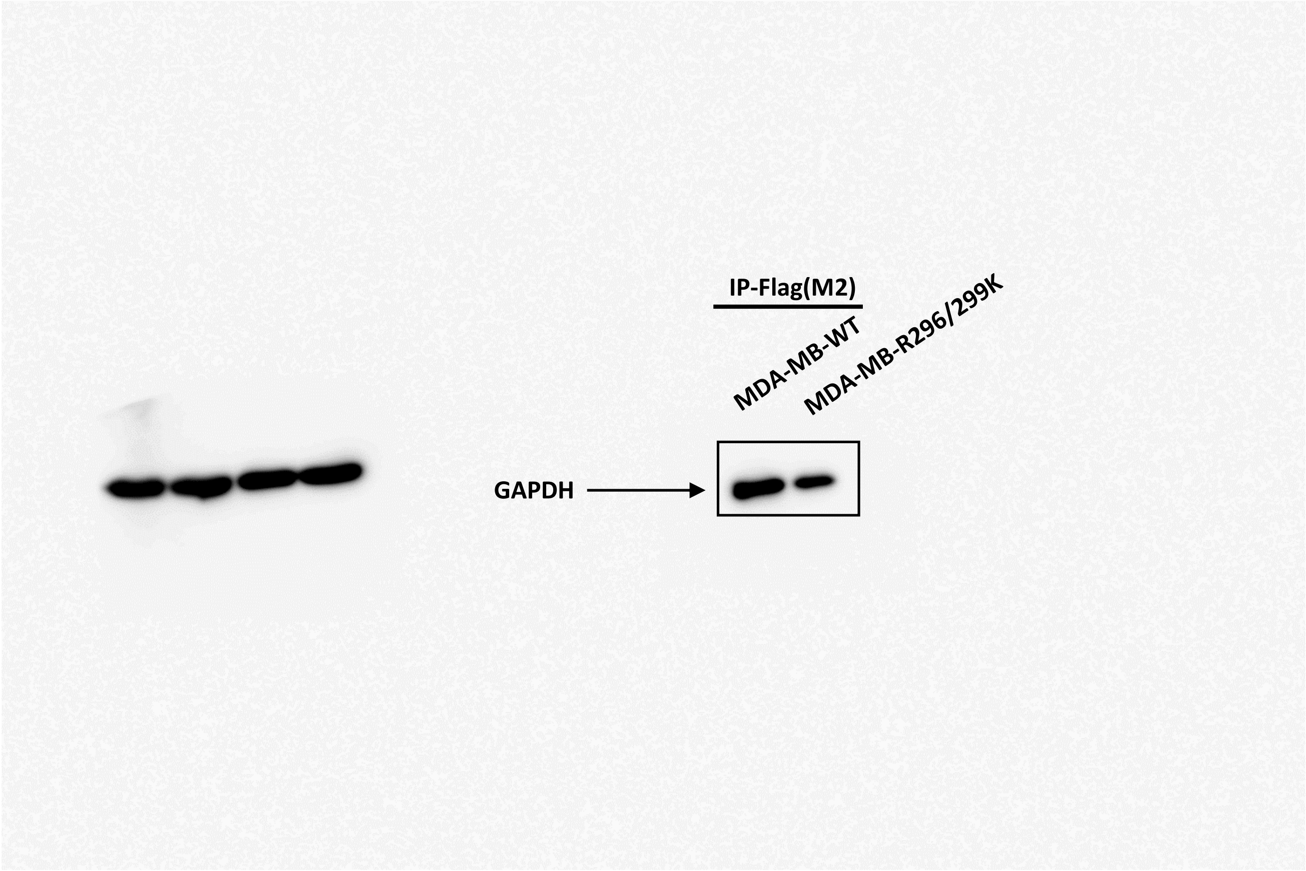

Supplementary figure 9. Continued.

For supplementary figure 1b

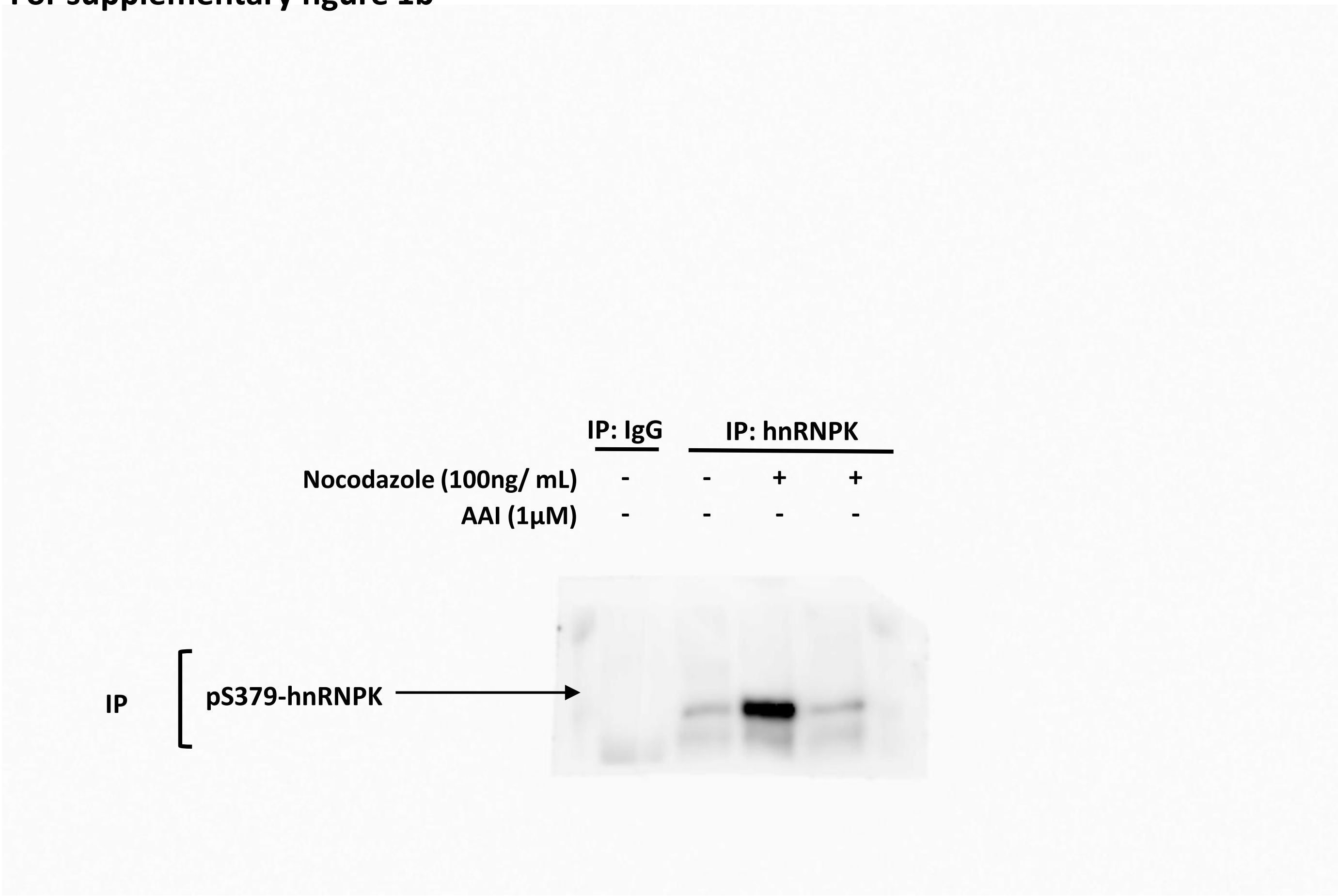

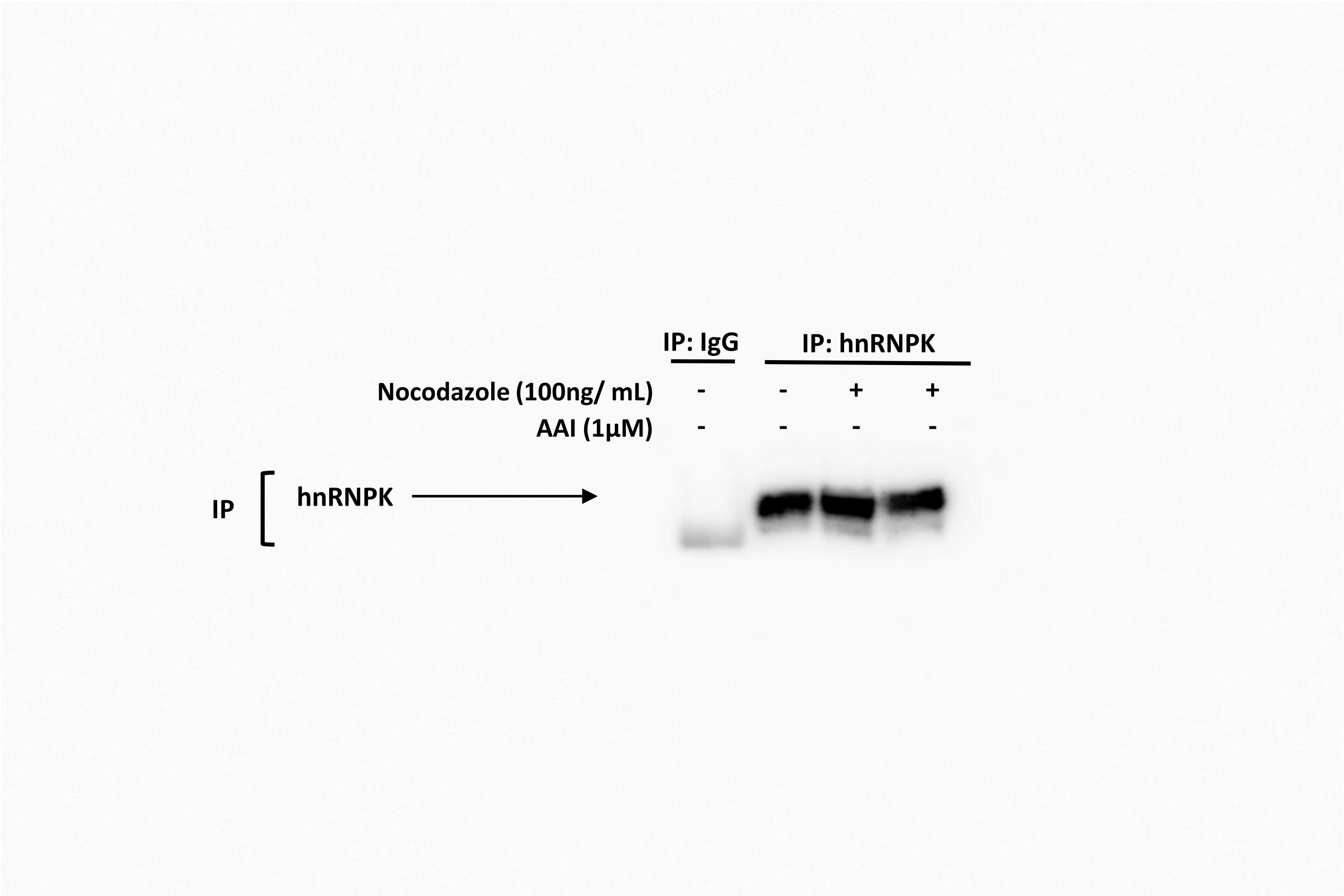

Supplementary figure 10. Raw images of the cropped blots presented in the supplementary figure 1.

For supplementary figure 1b

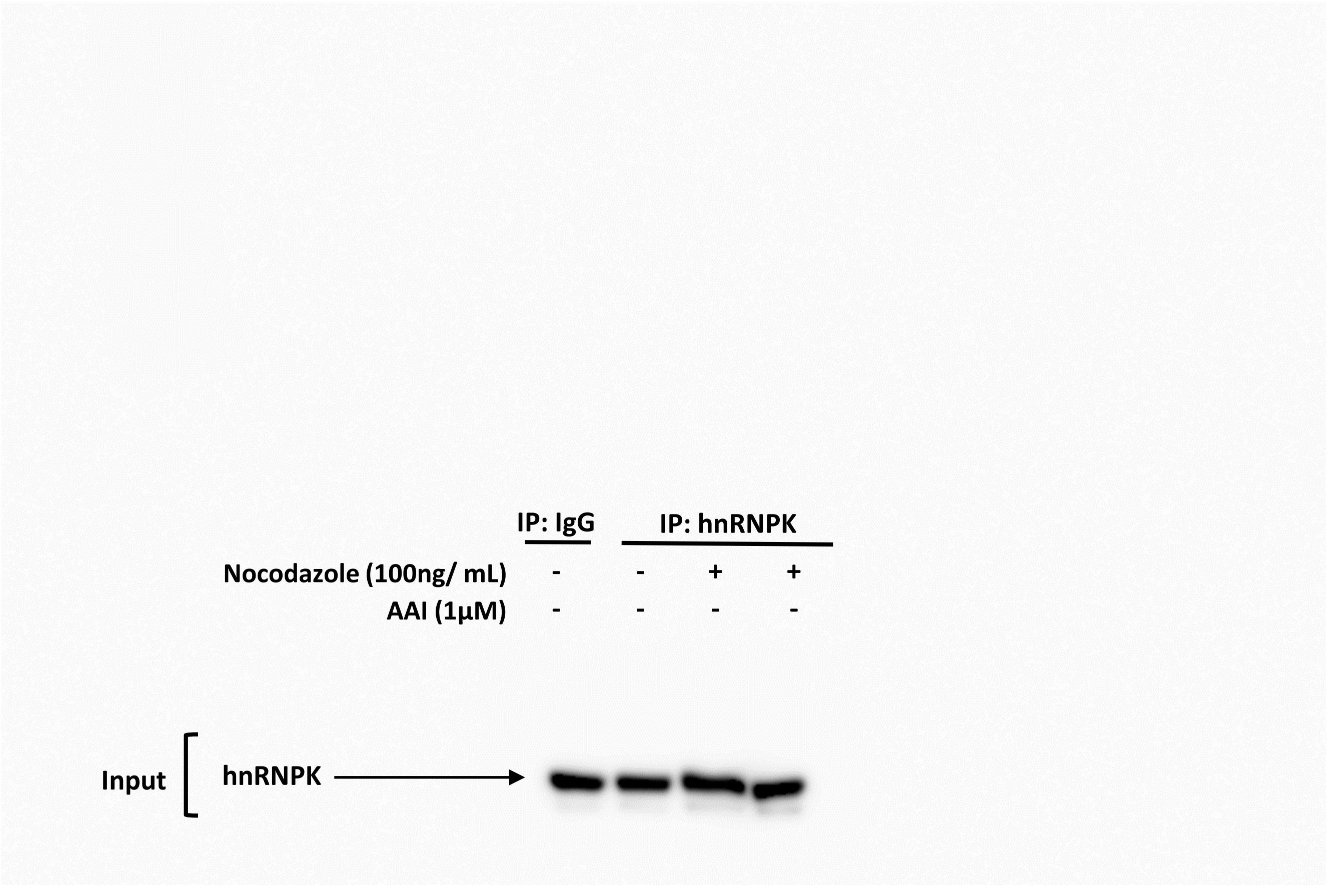

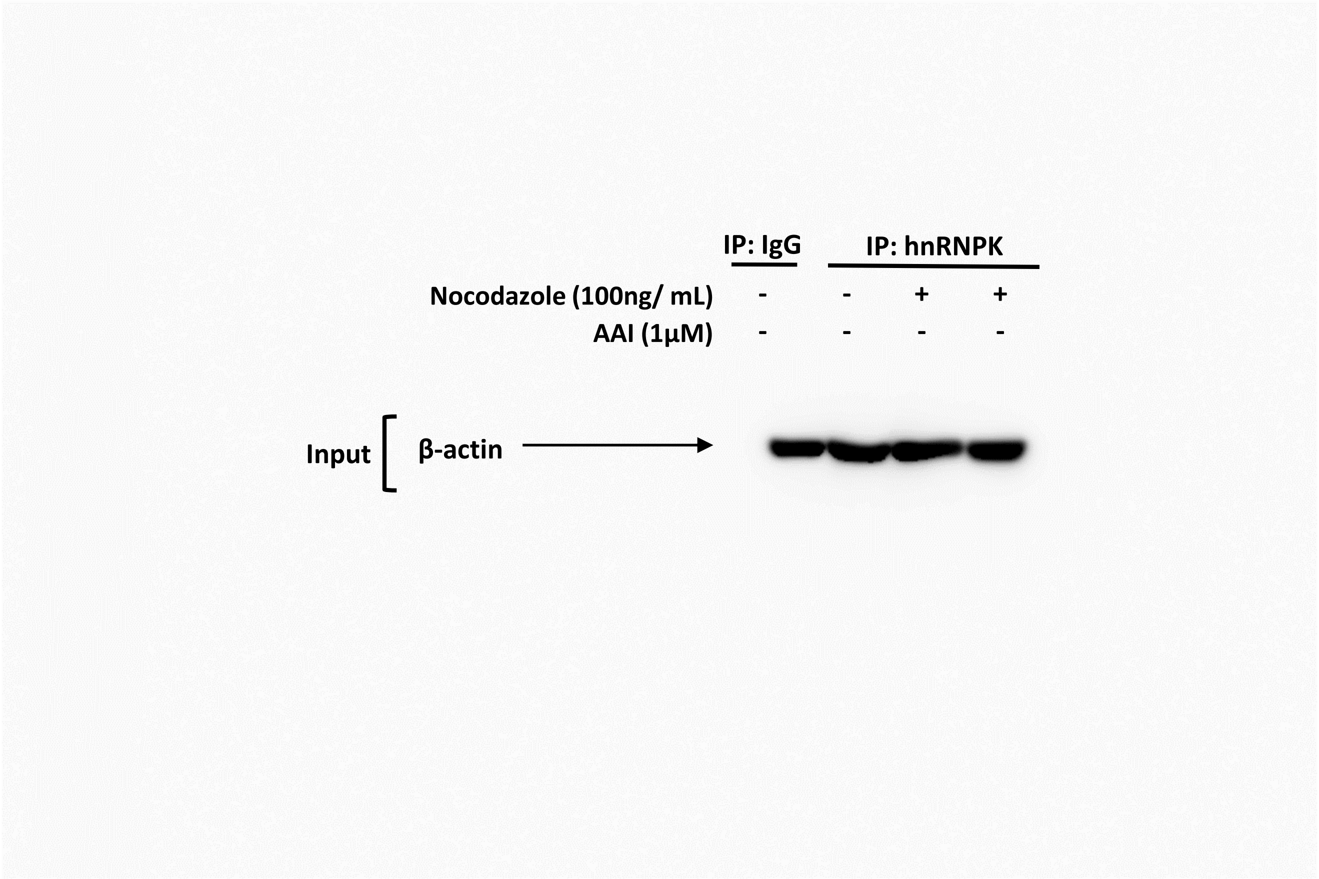

Supplementary figure 10. Continued.

For supplementary figure 3

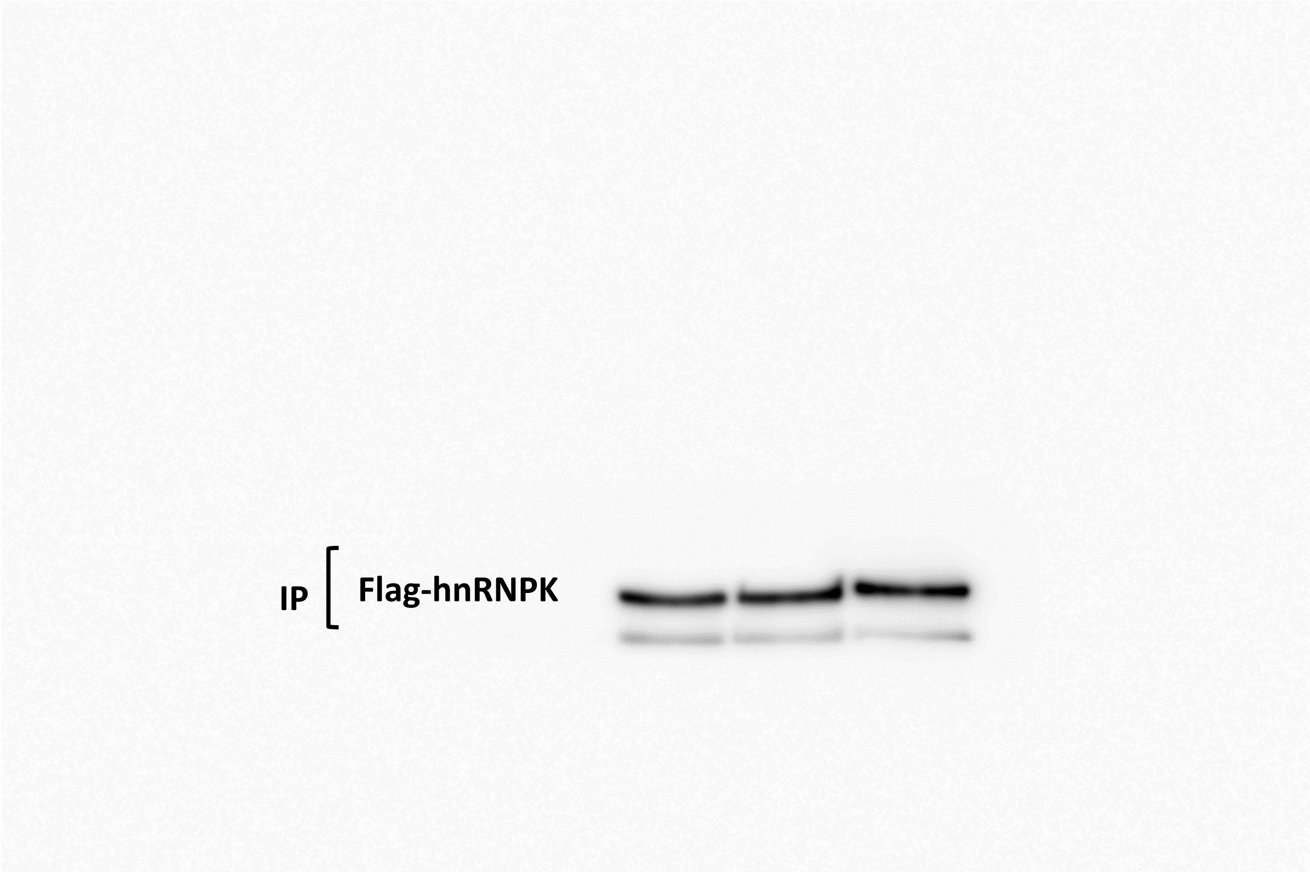

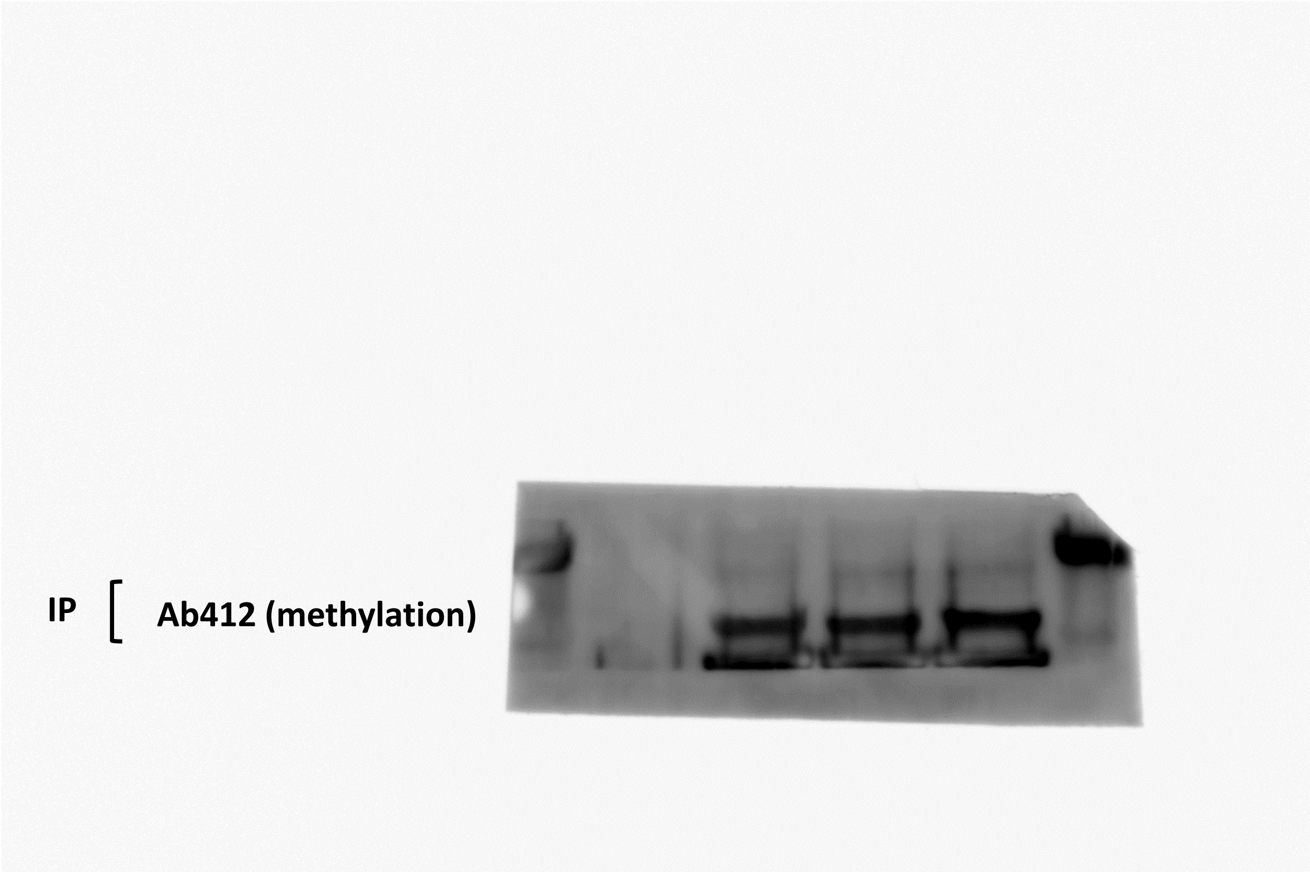

Supplementary figure 11. Raw images of the cropped blots presented in the supplementary figure 3.

For supplementary figure 3

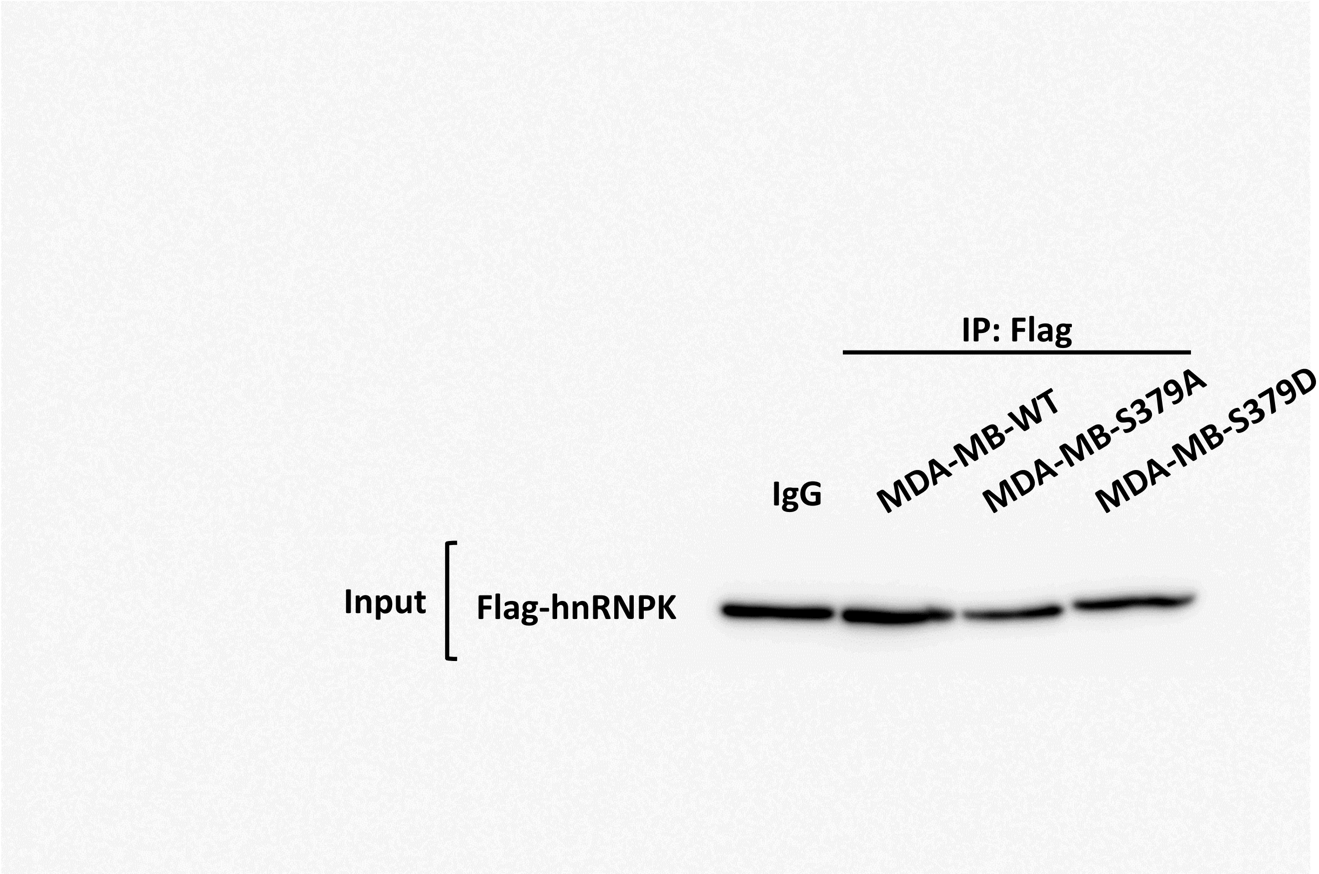

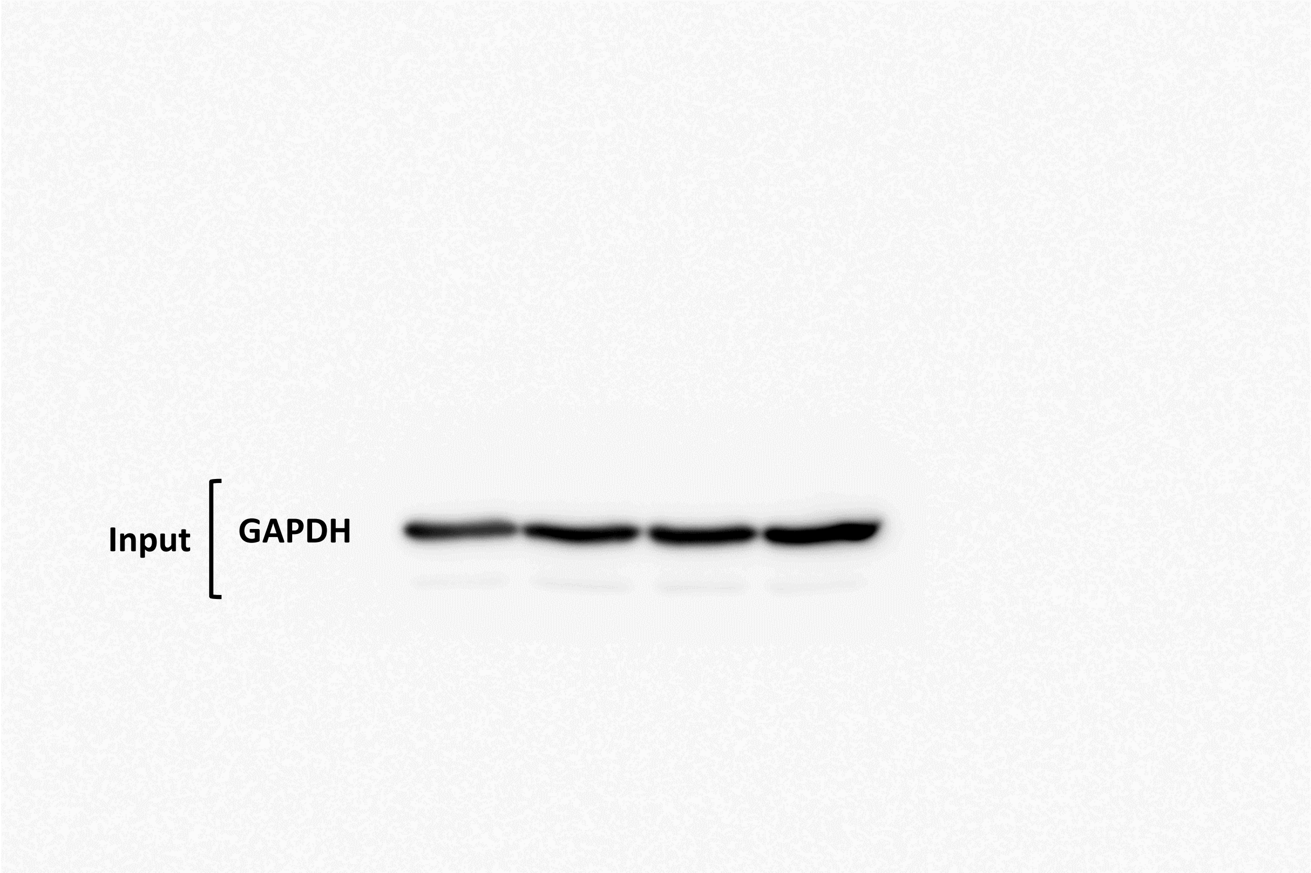

Supplementary figure 11. Continued.

For supplementary figure 4b

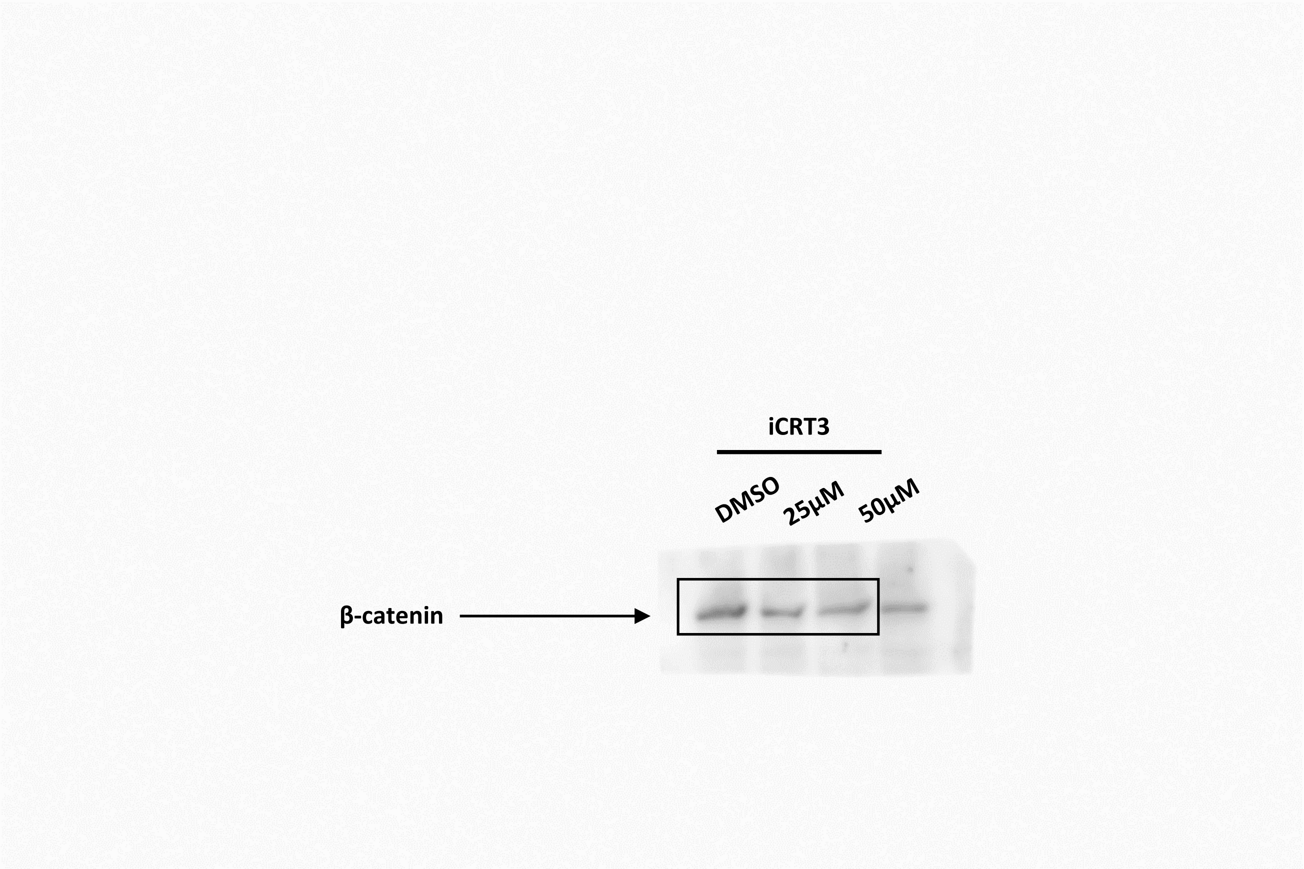

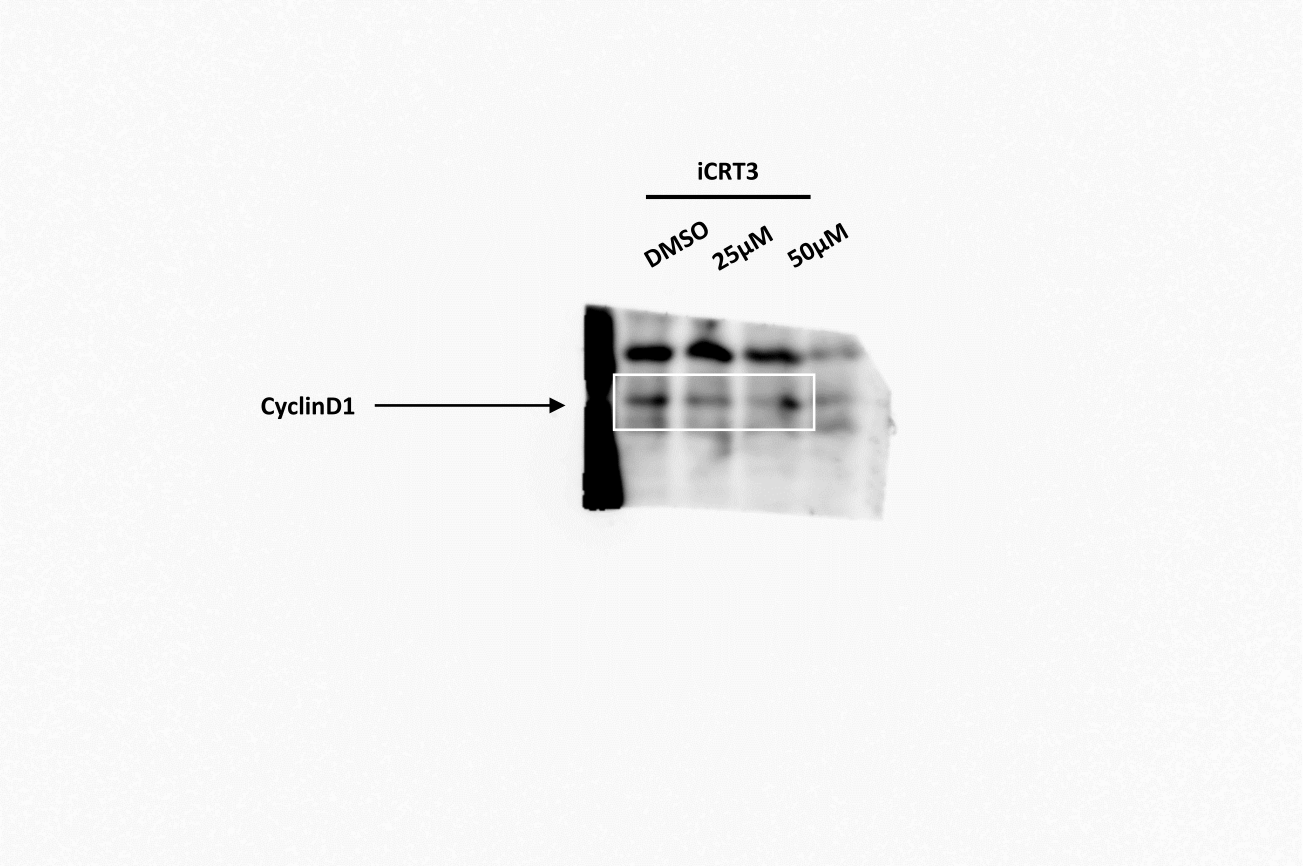

Supplementary figure 12. Raw images of the cropped blots presented in the supplementary figure 4.

For supplementary figure 4b

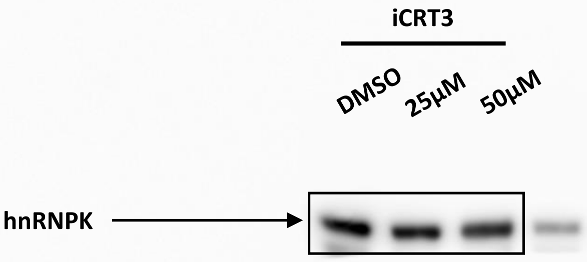

For supplementary figure S2b

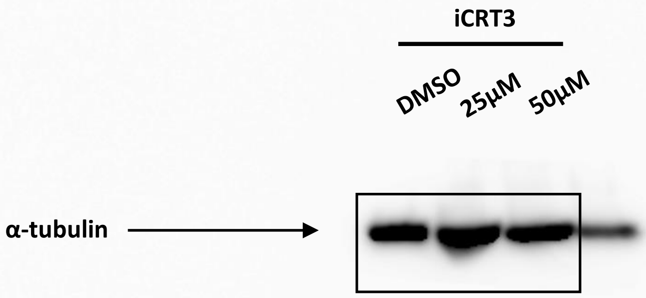

Supplementary figure 12. Continued.
